# A new domestic cat genome assembly based on long sequence reads empowers feline genomic medicine and identifies a novel gene for dwarfism

**DOI:** 10.1371/journal.pgen.1008926

**Published:** 2020-10-22

**Authors:** Reuben M. Buckley, Brian W. Davis, Wesley A. Brashear, Fabiana H. G. Farias, Kei Kuroki, Tina Graves, LaDeana W. Hillier, Milinn Kremitzki, Gang Li, Rondo P. Middleton, Patrick Minx, Chad Tomlinson, Leslie A. Lyons, William J. Murphy, Wesley C. Warren

**Affiliations:** 1 Department of Veterinary Medicine and Surgery, College of Veterinary Medicine, University of Missouri, Columbia, Missouri, United States of America; 2 Department of Veterinary Integrative Biosciences, Interdisciplinary Program in Genetics, College of Veterinary Medicine, Texas A&M University, College Station, Texas, United States of America; 3 Department of Psychiatry, Washington University, St. Louis, Missouri, United States of America; 4 NeuroGenomics and Informatics, Washington University, St. Louis, Missouri, United States of America; 5 Veterinary Medical Diagnostic Laboratory, College of Veterinary Medicine, University of Missouri, Columbia, Missouri, United States of America; 6 McDonnell Genome Institute, Washington University School of Medicine, St Louis, Missouri, United States of America; 7 Nestlé Purina Research, Saint Louis, Missouri, United States of America; 8 Donald Danforth Plant Science, St Louis, Missouri, United States of America; 9 Division of Animal Sciences, School of Medicine, University of Missouri, Columbia, Missouri, United States of America; HudsonAlpha Institute for Biotechnology, UNITED STATES

## Abstract

The domestic cat (*Felis catus*) numbers over 94 million in the USA alone, occupies households as a companion animal, and, like humans, suffers from cancer and common and rare diseases. However, genome-wide sequence variant information is limited for this species. To empower trait analyses, a new cat genome reference assembly was developed from PacBio long sequence reads that significantly improve sequence representation and assembly contiguity. The whole genome sequences of 54 domestic cats were aligned to the reference to identify single nucleotide variants (SNVs) and structural variants (SVs). Across all cats, 16 SNVs predicted to have deleterious impacts and in a singleton state were identified as high priority candidates for causative mutations. One candidate was a stop gain in the tumor suppressor *FBXW7*. The SNV is found in cats segregating for feline mediastinal lymphoma and is a candidate for inherited cancer susceptibility. SV analysis revealed a complex deletion coupled with a nearby potential duplication event that was shared privately across three unrelated cats with dwarfism and is found within a known dwarfism associated region on cat chromosome B1. This SV interrupted *UDP-glucose 6-dehydrogenase (UGDH)*, a gene involved in the biosynthesis of glycosaminoglycans. Importantly, *UGDH* has not yet been associated with human dwarfism and should be screened in undiagnosed patients. The new high-quality cat genome reference and the compilation of sequence variation demonstrate the importance of these resources when searching for disease causative alleles in the domestic cat and for identification of feline biomedical models.

## Author summary

The practice of genomic medicine is predicated on the availability of a high quality reference genome and an understanding of the impact of genome variation. Such resources have lead to countless discoveries in humans, however by working exclusively within the framework of human genetics, our potential for understanding diseases biology is limited, as similar analyses in other species have often lead to novel insights. The generation of Felis_catus_9.0, a new high quality reference genome for the domestic cat, helps facilitate the expansion of genomic medicine into the *Felis* lineage. Using Felis_catus_9.0 we analyze the landscape of genomic variation from a collection of 54 cats within the context of human gene constraint. The distribution of variant impacts in cats is correlated with patterns of gene constraint in humans, indicating the utility of this reference for identifying novel mutations that cause phenotypes relevant to human and cat health. Moreover, structural variant analysis revealed a novel variant for feline dwarfism in *UGDH*, a gene that has not been associated with dwarfism in any other species, suggesting a role for *UGDH* in cases of undiagnosed dwarfism in humans.

## Background

In the veterinary clinic, the practice of genomic medicine is impending [1]. With actionable genetic information in hand, companion animal therapeutic interventions are feasible, including treatment of animal patients prior to, or to prevent the appearance of, more severe symptoms and allow therapeutic administration of drugs with higher efficacy and fewer side effects. Genomic information can also alert veterinarians to imminent disease risks for diagnostic consideration. Each of these applications could significantly enhance veterinary medicine, however, none are currently in practice. As in human medicine, formidable challenges exist for the implementation of genomic-based medicine, including accurate annotation of the genome and databases of genetic variation from well-phenotyped individuals that include both single nucleotide variant (SNV) and structural variant (SV) discovery and annotation [2–4]. Targeted individual companion animal genome information is becoming more readily available, cost effective, and tentatively linked to the actionable phenotypes via direct-to-consumer DNA testing. Thus correct interpretation of DNA variants is of the utmost importance for communicating findings to clinicians practicing companion animal genomic medicine [5].

Companion animals suffer from many of the same diseases as humans, with over 600 different phenotypes identified as comparative models for human physiology, biology, development, and disease [6, 7]. In domestic cats, at least 70 genes are shown to harbor single and multiple DNA variants that are associated with disease [8] with many more discoveries expected as health care improves. The genetic and clinical manifestations of most of these known variants are described in the Online Mendelian Inheritance in Animals [6]. Examples include common human diseases such as cardiomyopathy [9], retinal degenerations [10], and polycystic kidney disease [11]. Veterinarians, geneticists and other researchers are actively banking DNA from companion animals and attempting to implement genomic medicine [1]. Once coupled with the quickly advancing sequencing technology and exploitable results, genomic medicine in companion animals promises to expand the comparative knowledge of mechanisms of action across species. Despite the continuing successful discovery of feline disease variants using both candidate gene and whole genome sequencing (WGS) approaches [1, 12–15], the understanding of normal and disease sequence variation in the domestic cat and interrogation of gene structure and function is limited by an incomplete genome assembly.

A fundamental hurdle hampering the interpretation of feline disease variant data is the availability of a high-quality, gapless reference genome. The previous domestic cat reference, Felis_catus_8.0, contains over 300,000 gaps, compromising its utility for identifying all types of sequence variation [16], in particular SVs. In conjunction with various mapping technologies, such as optical resolved physical maps, recent advances in the use of long-read sequencing and assembly technology has produced a more complete genome representation (i.e., fewer gaps) for many species [17–20].

Another hurdle for performing feline genomic medicine is the availability of WGS data from various breeds of cat with sufficient sequencing depth to uncover rare alleles and complex structural variants. Knowledge of variant frequency and uniqueness among domestic cats is very limited and is crucial in the identification of causal alleles. As a result of the paucity of sequence variant data across breeds, the 99 Lives Cat Genome Sequencing Initiative was founded as a centralized resource with genome sequences produced of similar quality and techniques. The resource supports researchers with variant discovery for evolutionary studies and identifying the genetic origin of inherited diseases and can assist in the development of high-density DNA arrays for complex disease studies in domestic cats [1, 21–24].

Here we present a new version of the domestic cat genome reference (Cinnamon, an Abyssinian breed), generated from deep sequence coverage of long-reads and scaffolding from an optical map (BioNano) and a high-density genetic linkage map [16]. Published cat genomes from the 99 Lives Cat Genome Consortium [1, 23] were aligned to the Felis_catus_9.0 reference to discover a plethora of unknown SNVs and SVs (multi-base insertions and deletions), including a newly identified structural variant (SV) for feline disproportionate dwarfism. Our case study of dwarfism demonstrates when disease phenotypes are coupled with revised gene annotation and sequence variation ascertained from diverse breeds, the new cat genome assembly is a powerful resource for trait discovery. This enables the future practice of feline genomic medicine and improved ascertainment of biomedical models relevant to human health.

## Results

### Genome assembly

A female Abyssinian cat (Cinnamon) was sequenced to high-depth (72-fold coverage) using real-time (SMRT; PacBio) sequence data and all sequence reads were used to generate a *de novo* assembly. Two PacBio instruments were used to produce average read insert lengths of 12 kb (RSII) and 9 kb (Sequel). The ungapped assembly size was 2.48 Gb and is comparable in size to other assembled carnivores (**Table 1**). There were 4,909 total contigs compared to 367,672 contigs in Felis_catus_8.0 showing a significant reduction in sequence gaps. The assembly contiguity metric of N50 contig and scaffold lengths were 42 and 84 Mb, respectively (**Table 1**). The N50 contig length of other PacBio sequenced carnivore assemblies are less contiguous, ranging from 3.13 Mb to 20.91 Mb (**Table 1**). Across carnivores, RepeatMasker showed consistent measures of total interspersed repeat content, (with 43% in Felis_catus_9.0; **S1 Table**) [25]. Due to repetitive and other genome architecture features, 1.8% (46 Mb) of all assembled sequences remained unassigned to any chromosome position. These sequences had an N50 scaffold length of 12,618 bp, demonstrating the assembly challenge of some repeat types in diploid genome assemblies, even of an inbred individual.

**Table 1:** Representative assembly metrics for various chromosome level assembled carnivore genomes^1^.

Assembly	Species	Breed	Isolate	Release date (MM/DD/YY)	Sequencing technology	Genome coverage	Total ungapped length (Gb)	Scaffold N50 (Mb)	Contig N50 (Mb)	Unplaced length (Mb)
Felis_catus_9.0	*Felis catus* (domestic cat)	Abyssinian	Cinnamon	11/20/17	PacBio; Bionano Genomics; 454 Titanium; Illumina; Sanger dideoxy sequencing	72x	2.48	83.97	41.92	46.02
Felis_catus_8.0	*Felis catus* (domestic cat)	Abyssinian	Cinnamon	11/07/14	Sanger; 454 Titanium; Illumina	2x Sanger; 14x 454, 20x Illumina	2.60	18.07	0.05	73.71
mLynCan4_v1.p	*Lynx canadensis* (Canada lynx)	NA	LIC74	07/26/19	PacBio Sequel I; 10X genome; Bionano Genomics; Arima Genomics Hi-C	72x	2.41	146.11	7.50	6.18
PanLeo1.0	*Panthera leo* (lion)	NA	Brooke	10/07/19	Illumina; Oxford Nanopore; 10X Genomics	46x	2.39	136.05	0.29	242.29
ASM864105v1	*Canis lupus familiaris* (dog)	German Shepherd	Nala	09/25/19	PacBio Sequel; Oxford Nanopore PromethION; Illumina (10X Chromium)	30x	2.40	64.35	20.91	22.83
ASM488618v2	*Canis lupus familiaris* (dog)	Basenji	MU ID 185726	08/16/19	Sequel	45x	2.41	61.09	3.13	120.80
UMICH_Zoey_3.1	*Canis lupus familiaris* (dog)	Great Dane	Zoey	05/30/19	PacBio RSII	50x	2.34	64.20	4.72	16.82
CanFam3.1	*Canis lupus familiaris* (dog)	Boxer	Tasha	11/02/11	Sanger	7x plus >90Mb finished sequence	2.39	45.88	0.27	75.10

^1^All species-specific assembly metrics derived from the NCBI assembly archive.

### Sequence accuracy and quality assessment

Illumina whole-genome sequence data from Cinnamon was used to identify reference sequence errors as homozygous SNVs. These numbered 60,449 in total, indicating a high level of sequence accuracy across assembled contigs (>99.9%). Sequence order and orientation was also highly accurate (>98%), as only 1.2% of BAC-end sequence alignments derived from Cinnamon were identified as discordant. Felis_catus_9.0 sequence order and orientation was also supported by high levels of agreement between individual chromosome sequence alignment and ordered sequence markers from the published cat genetic linkage map [16] (**S1 Data**). The raw sequence data, assembled contigs, and sequence order coordinates (AGP) were accessioned and are fully available by searching GCF_000181335.3 in GenBank.

### Gene annotation

The number of annotated protein-coding genes was very similar between the NCBI and Ensembl pipelines at 19,748 and 19,409, respectively. Approximately 376 protein-coding genes (NCBI) were identified as novel with no matching annotations in Felis_catus_8.0 (**S2 Data**). Conversely, 178 genes from Felis_catus_8.0 did not map to Felis_catus_9.0, of which the cause is unknown (**S1 Table**). A large portion of genes changed substantially (8.4%) in Felis_catus_9.0 during NCBI annotation (**S2 Data**). Aligned sequence of known same-species RefSeq transcripts (n = 420) to Felis_catus_9.0 is higher (99.5%) than Felis_catus_8.0 (97.8%) and the mean coverage of these same translated RefSeq proteins is also improved (90.1% versus 88.3% in Felis_catus_8.0). One important consequence of the less fragmented gene annotation is a 2% increase in aggregate sequence alignments of feline RNA-seq datasets to Felis_catus_9.0. These improvements are largely attributed to fewer assembly gaps. The various reported metrics of gene annotation quality conclusively show the protein-coding genes of the domestic cat are of high quality for all trait discovery studies. In addition, the annotation of repetitive elements was also compared across assembly versions using RepeatMasker output. Across the most common repeat classes, Felis_catus_8.0 carried slightly more repeat elements and fragments than Felis_catus_9.0 (**S2 Table**). However, deeper analysis of the two most common repeat classes, LINE/L1 and SINE/tRNA, showed that Felis_catus_9.0 carried far more full length LINE/L1 fragments than Felis_catus_8.0, indicating the superiority of the Felis_catus_9.0 assembly (**S1 Fig**).

### Genetic variation in cats

**Fig 1:**
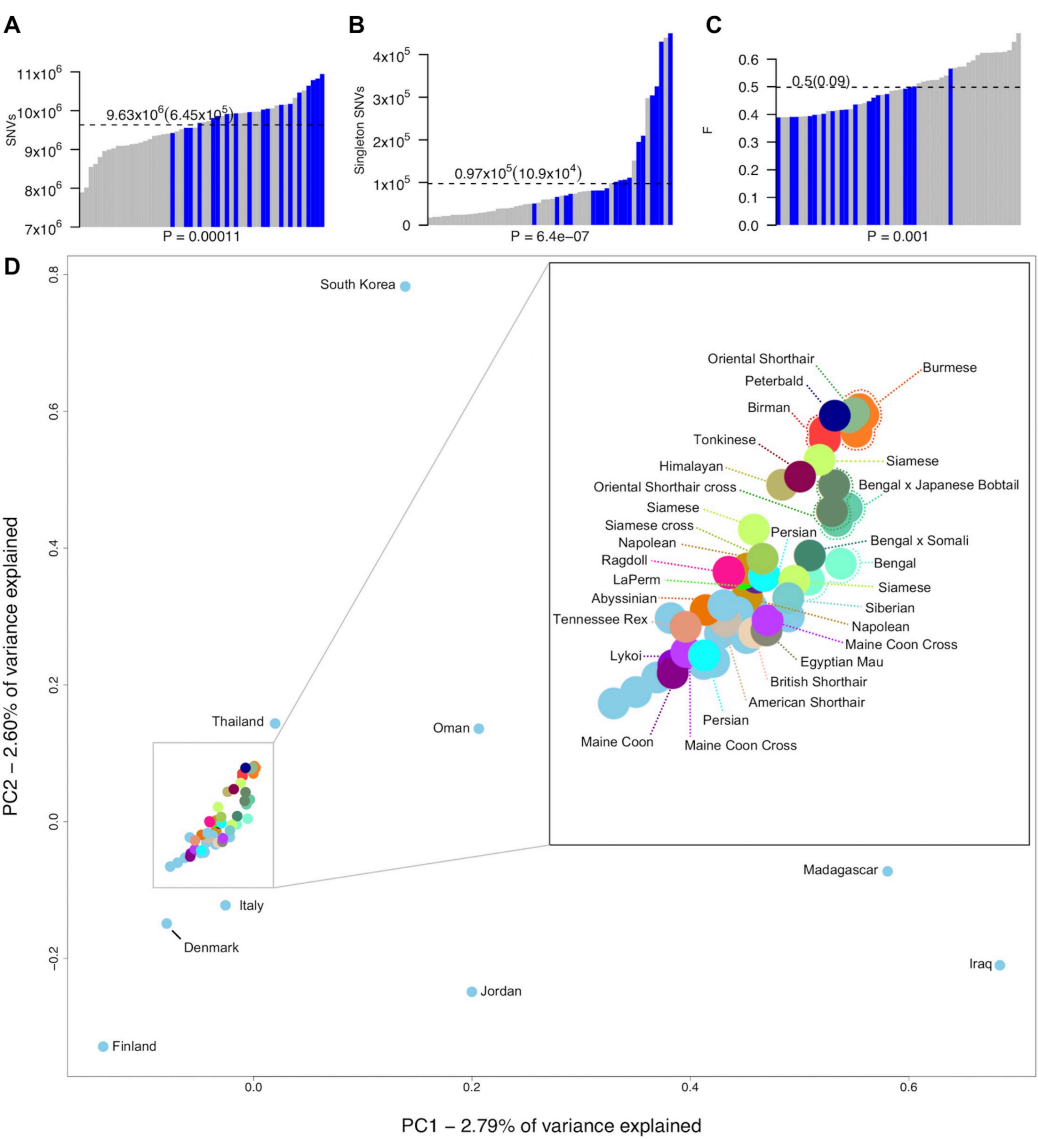
Genetic variation in whole genome sequenced cats. **(a)** Number of SNVs found in each genome, **(b)** singletons per genome, and **(c)** the per sample inbreeding coefficient *F*. Blue bars indicate each individual random bred cat and grey bars indicate each individual breed cat. P-values underneath each x axes were calculated using Wilcoxon rank-sum test and were used to compare breed cats to random bred cats. Dotted lines indicate the mean for each statistic, which is printed above along with standard deviation in braces. **(d)** Population structure of all unrelated cats (including Bengal breeds) estimated using principal components analysis. Random bred cats are colored light blue. Those that were sampled globally for diversity regions are named according to their sampling location. All other cats are named according to their breed or breed cross.

To improve variant knowledge of the domestic cat, variants from a diverse set of 74 resequenced cats from the 99 Lives project were analyzed in depth. The average sequence coverage was 38.5x with a mean of 98% reads mapped per cat (**S3 Data**). Approximately 46,600,527 variants were discovered, 39,043,080 were SNVs with 93% as biallelic displaying a Ts/Tv ratio of 2.44, suggesting a relatively high level of specificity for variant detection (**S3 Table**). In addition, probe sequences from the feline 63K genotyping array were mapped to Felis_catus_9.0 using blast. A total of 97% of these remapped SNV positions were detected as SNVs in the WGS call set (**S4 Table** and **S4 Data**) [26]. Using the variant data to estimate cat relatedness, 13 highly related cats (ϕ > 0.15), two cats with poor read quality, four bengals or bengal crosses, and Cinnamon, the reference, were removed from the sequence dataset to obtain a final set of 54 cats for all subsequent analyses (**S3 Data**). The average number of discovered SNVs per cat was 9.6 million (**Fig 1A**). Differences in SNV numbers varied according to whether cats were from a specific breed or were random bred (P-value < 0.005, Wilcoxon rank sum test), the two cats with the lowest number of SNVs (~8 million) were both Abyssinians, the same breed as Cinnamon, while random bred cats from either the Middle East or Madagascar each carried the highest number (> 10.5 million) (**S5 Data**). Individual singleton frequency and estimated inbreeding coefficients (*F* statistic) showed a similar trend with random bred cats generally having significantly more singletons and higher levels of heterozygosity than breed cats (P-value < 0.005, Wilcoxon rank sum test) (**Fig 1B and 1C**). Breed cats with higher levels of variation and heterozygosity were either from newly established breeds or were outcrossed individuals. For example, Napoleon cats, which all had an *F* statistic at least one standard deviation below the mean (**Fig 1C** and **S5 Data**), are frequently outcrossed as their defining trait dwarfism is likely homozygous lethal *in utero* [27].

PCA analysis showed the expected distribution of genetic relatedness among cats when considering their geographical location and genetic origins (**Fig 1D**). In general, most random bred cats displayed a scattered distribution consistent with previous studies on cat population diversity and origins [26, 28]. Although tightly clustered, breed cats could also be distinguished according to their populations of origin. The Asian-derived breeds, Siamese, Burmese, Birman, and Oriental shorthairs were at one end of the spectrum, clustering closely with random bred cats from Thailand. Conversely, cats derived from western populations, such as Maine Coons and Persians, were at the opposite end of the spectrum grouping with random bred cats from Northern Europe, such as Denmark and Finland.

### Implications of feline genetic variation on human disease genes

**Fig 2:**
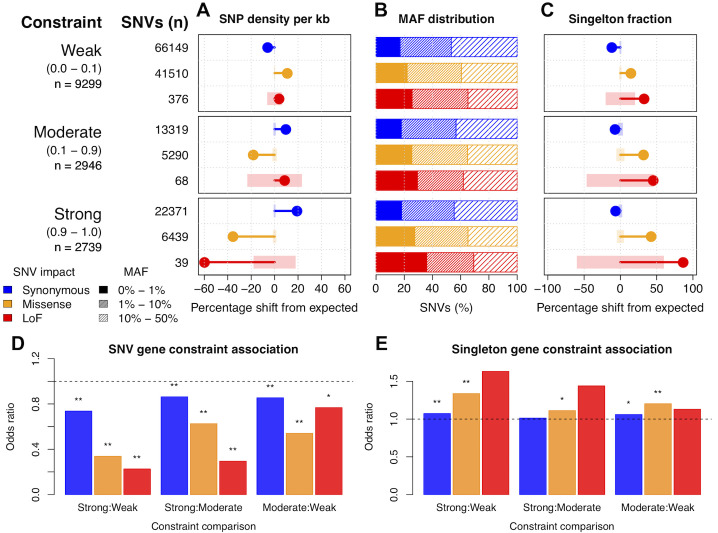
Deleterious SNVs in cats are uncommon and depleted from human constrained genes. To the left of the figure panels, constraint groups are labeled with pLI ranges shown below in braces. Below the pLI ranges are the number of genes found in each constraint group. SNV values show the total number of SNVs of a particular impact that belong to each constraint group. **(a)** Observed percentage differences from expected values for per kb SNV density. Light colored rectangles represent the 95% confidence intervals for the expected values. Confidence intervals were calculated using 10,000 permutations (methods). **(b)** The percentage of SNVs of different impacts and constraint groups across various MAF intervals. **(c)** Observed percentage differences from expected values for the fraction of SNVs in a singleton state (allele count of 1). Light colored rectangles represent the 95% confidence intervals for the expected values, generated from 10,000 permutations. **(d)** Pairwise SNV count contrasts between gene constraint groups and SNVs of the same impact. “*” indicates a *p*-value < 0.05 and “**” indicates a *p*-value < 0.001. **(e)** Pairwise singleton count contrasts between gene constraint groups and SNVs of the same impact. Asterisks above odds ratio values indicate the same values as (d).

To characterize feline genetic variation in disease contexts, variant effect predictor (VEP) was used to identify 128,844 synonymous, 77,662 missense, and 1,179 loss of function (LoF) SNVs, where SNVs causing a stop gain were the largest contributor to the LoF category (**S5 Table**) [29]. In addition to SNP annotation, genes were grouped according to their genetic constraint across human populations, where genetic constraint was expressed as probability of LoF intolerance [30]. In total, 15,962 cat-human orthologs were identified with 14,291 assigned pLI values. Of these, 9,299 were in the weak constraint group (pLI < 0.1), 2,946 were in the moderate constraint group (0.1 < pLI < 0.9), and 2,739 were in the strong constraint group (pLI > 0.9). For genes under weak constraint in humans, feline SNV density within coding sequences, regardless of impact on gene function, was similar to expected SNV densities based on random assignment of SNV impacts. Conversely, the density of SNVs in genes under strong constraint varied significantly according to SNV impact. LoF and missense SNVs, which have potential to deleteriously impact gene function, were depleted by 59.7% and 35.5%, respectively, while synonymous SNVs, which likely have no deleterious impact on gene function, were enriched by 19.2% relative to expected levels (**Fig 2A**). Similar results, while less pronounced, were also observed for synonymous and missense SNVs in genes under moderate constraint.

SNV minor allele frequency (MAF) distributions were compared across each constraint group. MAFs for synonymous SNVs were similarly distributed in all constraint groups. Conversely, the distribution of nonsynonymous SNVs increasingly skewed toward lower MAFs under stronger levels of constraint. For example, 25.5% of LoF SNVs in genes under weak constraint have a MAF < 1%, whereas 35.9% of LoF SNVs in genes under strong constraint have a MAF < 1% (**Fig 2B**). To determine whether these shifts in MAFs were significant, the fraction of SNVs in a singleton state were compared to expected levels based on random assignment of singleton states. Singleton states of SNVs were significantly enriched for nonsynonymous SNVs with enrichment levels increasing under stronger constraint, indicating many SNVs with functional impacts in genes under constraint are likely rare (**Fig 2C**). Together, these results show a significant association between selection in human genes and SNV accumulation in cats, suggesting selection pressure within cats and humans is similar across orthologous genes.

To further characterize the impact of gene constraint on feline genetic variation, SNV counts and singleton counts (**S6 Table**) within different constraint groups were directly contrasted using a fisher test. For SNV count contrasts, across all SNV impacts, SNVs were significantly depleted from genes of relatively increased constraint (**Fig 2D**). In strong to weak and strong to moderate comparisons, LoF SNVs has the lowest odds ratios below zero, indicating they were the most drastically depleted between genes of relatively higher constraint. Missense, SNVs were the next most depleted from genes of increased constraint, further indicating a positive relationship between SNV depletion, gene constraint, and SNV impacts on gene function. A similar relationship was also observed for singleton counts, where enrichment for singletons was associated with increased constraint and SNV impact (**Fig 2E**). However, while LoF SNVs showed the largest association between strong constraint and singleton SNVs, results were not significant below a *p*-value of 0.05. This is likely due to the count of LoF SNVs being too low in constrained genes being too low to reach significance. Alternatively, missense singleton associations with constrained were significant. Together, these results indicate that not only are SNVs in cats distributed non-randomly according to impact and gene constraint, but that differences in SNV distributions between constraint groups is also largely significant.

Overall, 16 LoF singleton SNVs were identified in intolerant orthologs as potential candidate disease causing variants. Since some cats within 99 lives had recorded disease statuses, these SNVs were assessed for their potential role in cat diseases (**S7 Table**). Of the 16 SNVs, four were supported by both Ensembl and NCBI annotations and were in cats segregating for particular diseases (**Table 2**). Potential causes for this discrepancy between annotation sources was investigated by viewing RNA-seq read coverage surrounding each of the 16 LoF singleton positions in NCBI’s genome browser. SNVs where gene annotations were consistent across both platforms generally had high levels of even read coverage across the entire exon (**S2 Fig**). SNVs where gene annotations were not consistent across platforms were either in regions of low RNA-seq coverage or uneven levels of RNA-seq coverage (**S3 Fig**). Many of these regions also had high levels of coverage of intron spanning RNA-seq reads. Of the four SNVs consistent across both annotation sources and in cats segregating for disease, the most notable is a stop gain in the tumor suppressor *F-box and WD repeat domain containing 7* (*FBXW7*) [31], which was only found in a parent and child segregating for feline mediastinal lymphoma. Other LoF SNVs include stop gains found in *Family With Sequence Similarity 13 Member B* (*FAM13B*) in a random bred with ectodermal dysplasia, cytoplasmic FMR1 interacting protein 2 (*CYFIP2*) in an Egyptian Mau with urate stones, and *SH3 And PX Domains 2A* (*SH3PXD2A*) in a random bred cat with feline infectious peritonitis. Most candidates are not likely disease causing, as each cat carried a mean of 10.0 LoF SNVs in strongly constrained genes (**S4 Fig**). However, while most LoF SNVs had MAFs > 10%, the mean number of LoF SNVs with MAF < 1% in strongly constrained genes was 0.26 per cat (**S5 Fig**). These results suggest gene intolerance to mutations may provide as a useful metric for reducing the number of candidate variants for certain diseases.

**Table 2:** High impact singletons in intolerant orthologs for unrelated cats with disease traits.

SNV location^a^	Ref/Alt	Consequence	Gene symbol	pLI	Individual ID	Disease	Status
**chrA1:116653102**	**G/A**	**Stop gained**	***FAM13B***	**0.92**	**felCat.Fcat19194.Pudge**	**Ectodermal dysplasia**^b^	**Affected**
**chrA1:192824838**	**G/A**	**Stop gained**	***CYFIP2***	**1.00**	**felCat.Fcat20406.Gannon**	**Stones**	**Affected**
**chrB1:77305060**	**C/T**	**Stop gained**	***FBXW7***	**1.00**	**felCat.Fcat5012.Colorado**^c^	**Lymphoma**	**Carrier**
**chrD2:63980875**	**G/A**	**Stop gained**	***SH3PXD2A***	**1.00**	**felCat.CR1397.Isabella**	**Infectious peritonitis**^d^	**Affected**

^a ^SNV locations were only reported if they were supported by NCBI annotations.

^b ^A second unrelated cat, felCat.Fcat19197.Kooki, was also affected.

^c^ Affected offspring removed earlier from analysis inherited the same SNV.

^d ^A second unrelated cat, felCat.CR1219.Tamborine, was also affected.

### Structural variant discovery

**Fig 3:**
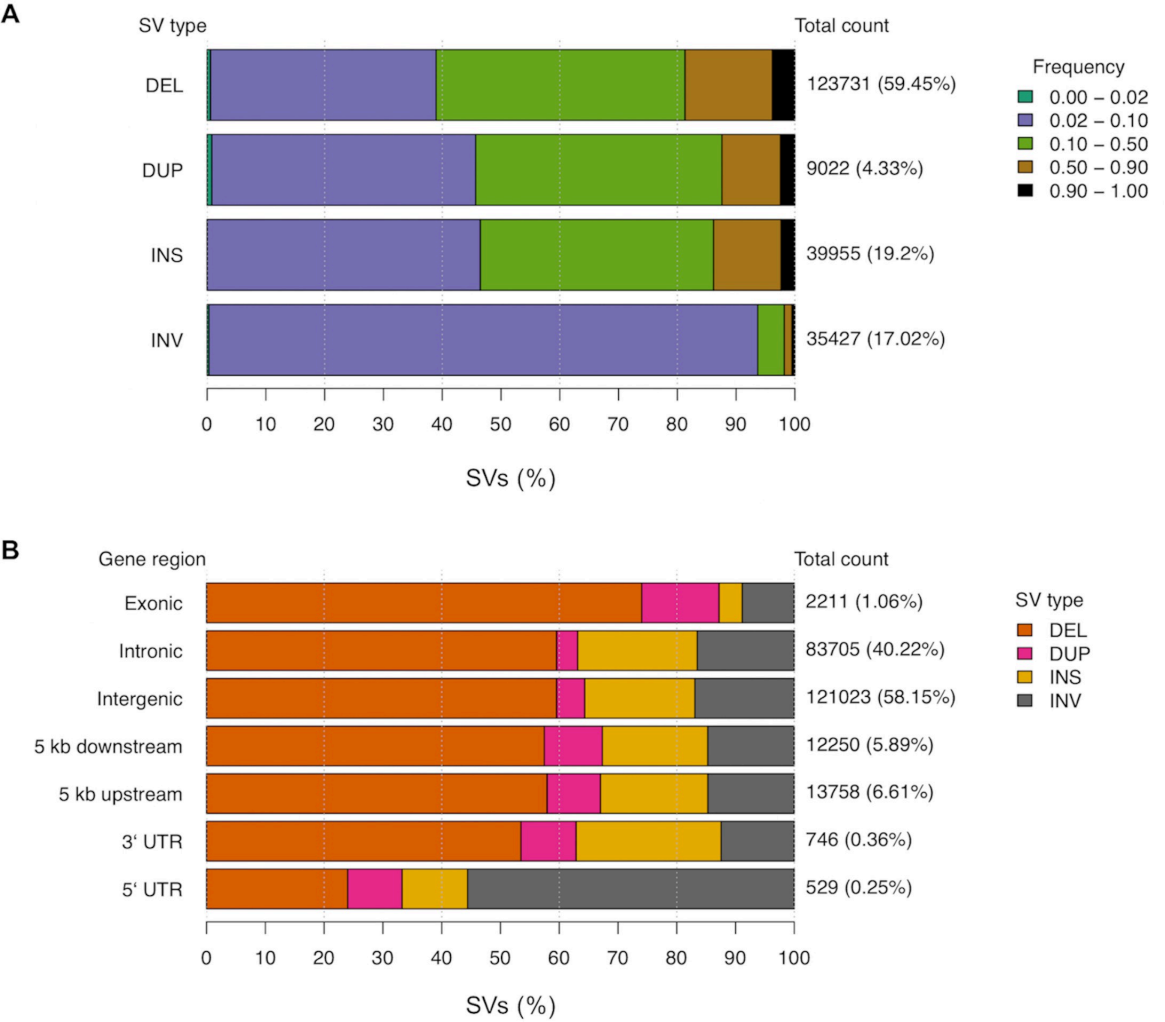
The structural variant landscape of cats. **(a)** Population frequency of each SV type. Colored bars represent the proportion of a given SV type found within various population frequency ranges. Total count values to the right of each bar represent the total number of SVs of each type, while percentage values in braces represent the proportion of all SVs that belong to each type. Frequency ranges shown in the legend are ordered along each bar from left to right. **(b)** The proportion of SVs found in each genomic region. Colored bars represent the proportion of SVs belonging to a particular type found across different genomic regions. Similar to above, the total count values to the right of each bar represent the total number of SVs found in each genomic region. The percentage values in braces represent the genome-wide proportion of all SVs found in each gene region. These values sum greater than 100 percent as a single SV can span multiple types of genomic regions. Structural variants are noted as: deletion (DEL); duplication (DUP); insertion (INS); inversion (INV).

The merging of the two independent SV call sets was performed across all individuals for variants occurring within 50 bp of the independent variant call position, with agreement on variant type and strand, and variant size within 500 bp. Per cat, an average of 44,990 SVs were identified, with variants encompassing 134.3 Mb across all individuals. Deletions averaged 905 bp, duplications 7,497 bp, insertions 30 bp, and inversions 10,993 bp. The breed and breed crosses (n = 36) compared to random bred cats (n = 18) showed comparable SV diversity (t-test p = 0.6) (**S6 Fig**). In total, 208,135 SVs were discovered, of which 123,731 (60%) were deletions (**Fig 3A**). SV population frequencies were similar across SV types, except for inversions. For deletions, duplications and insertions, 38% to 48% of each SV type was found at population frequencies of 0.02–0.10 and 0.10–0.50. Meanwhile, > 90% of inversions are found at a population frequency of 0.02–0.10 (**Fig 3A**). The majority of SVs identified are common across cats, suggesting their impacts are mostly tolerated.

SV density across autosomes was relatively constant with chromosome E1 carrying the largest SV burden at 96.95 SVs per Mb (**S7 Fig)**. Approximately 6,096 SVs (3%, 10.1 Mb) were observed in >90% of the cat genomes (**S6 Data**), indicating the cat used for the Felis_catus_9.0 assembly, Cinnamon, an Abyssinian, likely carries a minor allele at these positions. SV annotation showed that SV counts per region were consistent with the fraction of the genome occupied by each region type. For example, 58.15% of SVs were intergenic, 40.22% of SVs were intronic, and 1.06% of SVs were exonic, potentially impacting 217 different protein coding genes (**Fig 3B** and **S7 Data**). Conversely, the proportion of some SV types found in certain gene regions varied from their genome-wide averages. For example, in regions 5 kb upstream and downstream of genes, duplications were increased approximately two-fold. For exonic regions, 74% of SVs were deletions, an increase form the genome wide level of 59.45%. For 5`UTRs, the majority of SVs were inversions, which only represent 17.02% of total SVs. These results suggest an interaction between the impact of SV types and the potential function of the gene regions they are found in.

### Genetics of feline dwarfism

**Fig 4:**
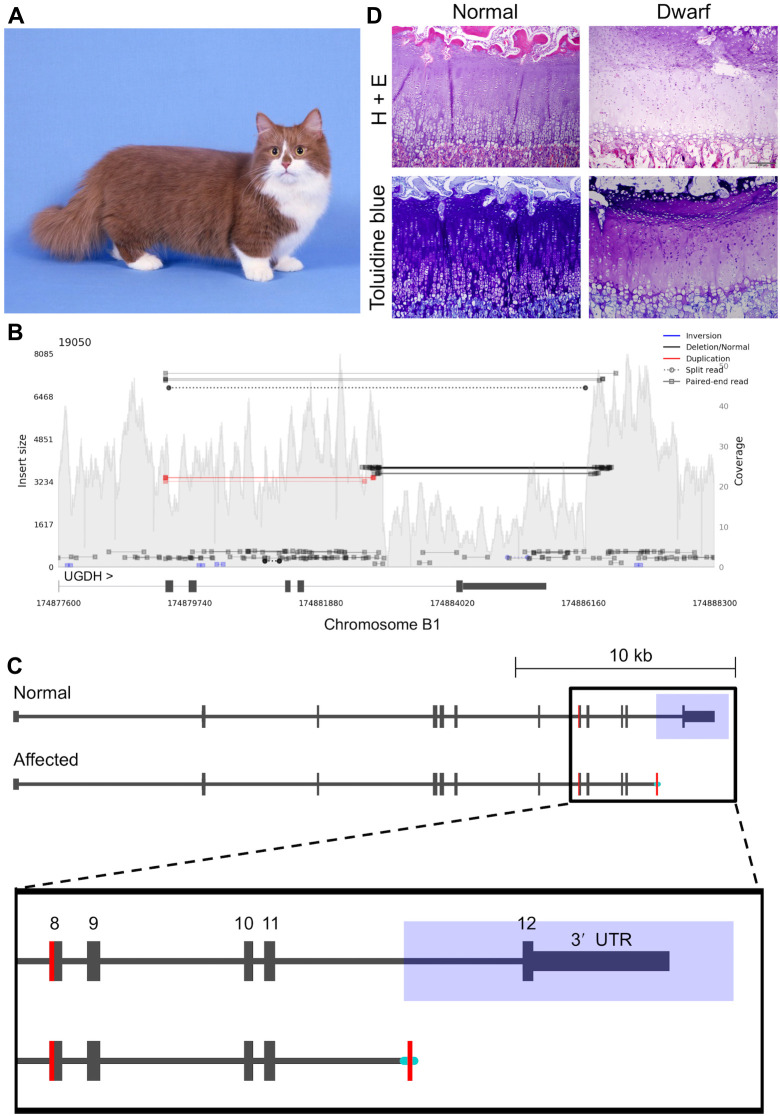
A complex structural variant is associated with feline dwarfism. **(a)** Image of dwarf cat from the Munchkin breed. Notice that limbs are short while torso remains normal size. Image was donated courtesy of Terri Harris. **(b)** Samplot output of discordant reads within an affected cat. Decreased coverage over the final exon of *UGDH* represents a deletion. Discordant reads spanning beyond the deleted region show newly inserted sequence sharing homology with *UGDH* exon 8. **(c)** Schematic illustrating the candidate structural variant for dwarfism. Blue rectangle overlaps deleted region, shaded portion of exon 8 shows potential duplication in the affected, and cyan lines indicate inserted sequence of unknown origin. **(d)** Hematoxylin and eosin and toluidine blue histologic samples of the distal radius epiphyseal cartilage plate from a neonatal kitten and an age-matched dwarf kitten. The normal kitten showed regular columnar arrangement of chondrocytes with abundant proteoglycan as determined by strong metachromasia with toluidine blue stain. The dwarf kitten showed disorganized columnar arrangement of chondrocytes with proteoglycan depletion as determined by weak metachromasia with toluidine blue stain.

Dwarfism in cats is the defining feature of the Munchkin breed and is characterized by shortened limbs and normal sized torso (**Fig 4A**) [32]. Similar to analyses with cat assemblies Felis_catus-6.2 and Felis_catus_8.0, previous investigations for SNVs in the new Felis_catus_9.0 assembly did not identify any high priority candidate variants for disproportionate dwarfism [27]. However, SV analysis within the critical region previously identified by linkage and GWAS on chromosome B1:170,786,914–175,975,857 [27] revealed a 3.3 kb deletion at position chrB1:174,882,897–174,886,198, overlapping the final exon of *UDP-glucose 6-dehydrogenase (UGDH)* (**Fig 4B**). Upon manual inspection of this SV, a 49 bp segment from exon 8 appeared to be duplicated and inserted 3.5 kb downstream, replacing the deleted sequence. This potentially duplicated segment was flanked by a 37 bp sequence at the 5`end and a 20 bp sequence at the 3’ end, both of unknown origin (**Fig 4C**). Discordant reads consistent with the SV were private to all three unrelated WGS dwarf samples (**S8 Fig**). The breakpoints surrounding the deletion were validated in WGS affected cats with Sanger sequencing. PCR-based genotyping of the 3.3 kb deletion breakpoints was conducted in a total of 109 cats including, 41 normal and 68 affected dwarf cats (**S9 Fig**). Expected amplicon sizes and phenotypes were concordant across all cats, except for a “Munchkin; non-standard (normal legs); Selkirk mix”, which appeared to carry the mutant allele, suggesting an alternate causal gene or sampling error (**S8 Data**). No individuals homozygous for the SV were observed and genotypes were not in Hardy-Weinberg equilibrium (*X*^2^ = 23.4, *p*-value < 0.001). Moreover, out of 11 individuals with two heterozygous affected parents, 7 cats were heterozygous for the SV and 4 cats were homozygous reference, which is consistent with the expected ratio of genotypes for a recessive lethal allele.

As *UGDH* has known roles in proteoglycan synthesis in chondrocytes [33, 34], growth plates in two healthy and seven dwarf neonatal kittens were histologically examined for structural irregularities and proteoglycan concentrations (Methods). The articular cartilage or bone in the dwarf specimens had no significant histopathologic changes. However, the epiphyseal plate, while present in all specimens, exhibited a disorganized columnar arrangement in dwarf specimens. In addition, dwarf specimens also showed proteoglycan depletion as determined by toluidine blue stain (Figs **4D** and **S10**). Conversely, in the two normal kittens, chondrocytes in the epiphyseal plate exhibited a regular columnar arrangement with organization into a zone of reserve cells, a zone of proliferation, a zone of hypertrophy and a zone of provisional calcification (Figs **4D** and **S10**). Moreover, the articular-epiphyseal cartilage complex and the epiphyseal plate in the distal radius from these two normal kittens contained abundant proteoglycan.

## Discussion

Studies of the domestic cat show the amazing features of this obligate carnivore, including their incomplete domestication, wide range of coat color variation, and use as biomedical models [11, 35–37]. Using a combination of long-reads, combined with long-range scaffolding methods, Felis_catus_9.0 was generated as a new cat genome resource, with an N50 contig length (42 Mb) surpassing all other carnivore genome assemblies. This sequence contiguity represents a 1000-fold increase of ungapped sequence (contigs) length over Felis_catus_8.0, as well as a 40% reduction in the amount of unplaced sequence. All measures of sequence base and order accuracy, such as the low number of discordant BAC end sequence alignments, suggest this reference will strongly support future resequencing studies in cats. Equally important are the observed improvements in gene annotation including the annotation of new genes, overall more complete gene models and improvements to transcript mappability. Only 178 genes were missing in Felis_catus_9.0 compared to Felis_catus_8.0, which will require further investigation. However, 376 predicted genes are novel to Felis_catus_9.0, i.e. not found in Felis_catus_8.0.

Using this Felis_catus_9.0 assembly, a vast new repertoire of SNVs and SVs were discovered for the domestic cat. The total number of variants discovered across our diverse collection of domestic cats was substantially higher than previous studies in other mammals, such as, cow [38], dog [39], rat [40–42], sheep [43, 44], pig [45], and horse [46]. Even rhesus macaques, with twice as many variants as human, do not approach the same levels of cat SNV variation [47, 48]. The cats had 36.6 million biallelic SNVs with individuals carrying ~9.6 million SNVs each. Conversely, humans roughly carry 4 to 5 million SNVs per individual [49]. One possible explanation for this discrepancy may be the unique process cats underwent for domestication. Rather than undergoing strong selective breeding leading to a severe population bottleneck, cats were instead “self” domesticated, never losing some ancestral traits such as hunting behavior [50–53]. The practice of strong selective breeding in cats only began in recent history and is based almost exclusively on aesthetic traits. Consistent with previous analyses, our evaluation of breeds and random bred cats showed tight clustering between cats from different breeds that suggest cat breeds were likely initiated from local random bred cat populations [28, 54]. In regards to per sample numbers of variants, most breed cats had fewer SNVs than random bred cats. Likewise, random bred cats also had higher numbers of singletons and a lower inbreeding coefficient than breed cats, suggesting breed cats may share a common genetic signature distinct from random bred populations.

For genetic discovery applications, animals with lower levels of genetic diversity are often more desirable as they reduce experimental variability linked to variation in genetic architecture [55]. However, with decreasing costs of genome or exome sequencing leading to exponential growth in variant discovery, animals with higher genetic diversity, such as cats, could enhance discovery of tolerable loss of function or pathogenic missense variants. Similar to cats, rhesus macaques from research colonies, exhibited per sample SNV rates more than two-fold greater than human [48]. Importantly, macaque genetic diversity has been useful for characterizing non-deleterious missense variation in humans [56].

A major premise of our feline genomics analysis is that increased discovery of genetic variants in cats will help improve benign/pathogenic variant classification and reveal similarities between human and feline genetic disease phenotypes, aiding disease interpretation in both species. To gain insights into the burden of segregating variants with potentially deleterious impacts on protein function, SNV impacts were classified across 54 unrelated domestic cats. Overall, cat genes identified as being under strong constraint in humans were depleted of nonsynonymous SNVs and enriched for potentially rare variants, showing both species harbor similar landscapes of genetic constraint and indicating the utility of Felis_catus_9.0 in modeling human genetic disease. An important limitation on the analysis was the available sample size. As there were only 54 cats, true rare variants, defined as variants with allele frequency of at least 1% in the population [57], could not be distinguished from common variants that appear in only one of 54 randomly sampled cats by chance. Instead, singleton SNVs were focused on as candidates for rare variants. Altogether, 18.6% of all feline SNVs could be considered candidates for rare variants. Alternatively, larger analyses in human reveal a much higher fraction of low frequency variants [58, 59]. For example, 36% of variants in an Icelandic population of over 2000 individuals had a minor allele frequency below 0.1% [59]. Similarly sized analyses in other mammals reported ~20% of variants had allele frequencies < 1% [44, 46, 48]. As the number of cats sequenced increases, the resolution to detect rare variants, as well as the total fraction of low frequency variants, will continue to grow linearly as each individual will contribute a small number of previously undiscovered variants [60].

Focusing specifically on potentially rare variants with high impacts in constrained genes identified a potential cause for early onset feline mediastinal lymphoma, a stop gain in tumor suppressor gene *FBXW7* [31]. Feline mediastinal lymphoma is distinct from other feline lymphomas in its early onset and prevalence in Siamese cats and Oriental Shorthairs [61], suggesting a genetic cause for lymphoma susceptibility specific to Siamese related cat breeds [62, 63]. The stop gain was initially observed in a heterozygous state in a single cat identified as a carrier in the unrelated set of cats. Subsequent analysis of the full set of cats revealed the SNV had been inherited in an affected offspring. Despite discordance between the presence of this mutant allele and the affection status of the cats it was found in, the variant still fits the profile as a susceptibility allele for mediastinal lymphoma. For example, homozygous knockout of *Fbxw7* is embryonic lethal in mice [64, 65], while heterozygous knockout mice develop normally [65]. However, irradiation experiments of *Fbxw7*^+/−^ mice and *Fbxw7*^+/-^*p53*^+/-^ crosses identify *Fbwx7* as a haploinsufficient tumor suppressor gene that requires mutations in other cancer related genes for tumorgenesis [66], a finding supported in subsequent mouse studies [67, 68]. Similarly, in humans, germline variants in *FBXW7* are strongly associated with predisposition to early onset cancers, such as Wilms tumors and Hodgkin’s lymphoma [69, 70]. Screening of Siamese cats and other related breeds will validate the *FBWX7* stop gain as a causative mutation for lymphoma susceptibility and may eventually aid in the development of a feline cancer model.

An important concern with using human constraint metrics for identifying causative variants in cats is the potential for false positives. For example, out of 16 SNVs initially identified as potential feline disease candidates, many belonged to cats with no recorded disease status (**S7 Table**). However, since a large fraction of healthy humans also carried SNVs matching similar disease causing criteria as used in cats [58], evolutionary distance between humans and cats is unlikely to be a significant contributing factor to false positive candidate disease variant identification. Instead, the frequency of high impact variants in constrained genes is likely due to the limited resolution provided by analyses at the gene level. Recent strategies in humans have confronted this problem by focusing on constraint at the level of gene region. These analyses identified many genes with low pLI values that contained highly constrained gene regions that were also enriched for disease causing variants [71]. Alternatively, another strategy for further refining constrained regions could involve combining genomic variation from multiple species. Many variants observed in cats, along with their impact on genes, are likely unique to cats and could potentially be applied to variant prioritization workflows in humans.

Presented here is the first comprehensive genome-wide SV analysis for Felis_catus_9.0. Previous genome-wide SV analyses in the cat were performed in Felis_catus-6.2 and focused solely on copy number variation (CNV) [24]. Approximately 39,955 insertions, 123,731 deletions, 35,427 inversions, and 9,022 duplications were identified, far exceeding previous CNV calculations of 521 losses and 68 gains. This large discrepancy is likely due to a number of reasons including filtering stringency, sensitivity, and reference contiguity, where increased numbers of assembly gaps in previous reference genome assemblies hindered detection of larger SV events. An important difference regarding filtering stringency and sensitivity is that the average CNV length was 37.4 kb as compared to 2.7 kb for SVs in Felis_catus_9.0. Since CNV detection depends on read-depth [72, 73], only larger CNVs can be accurately identified, as coverage at small window sizes can be highly variable. The use of both split-read and read-pair information [74, 75], allowed the identification of SV events at much finer resolution than read-depth based tools [76].

Improved sensitivity for smaller SV events, while helpful for finding new disease causing mutations, may also lead to increased false positive SV detection. In three human trios sequenced with Illumina technology, LUMPY and Delly were both used to identify 12,067 and 5,307 SVs respectively, contributing to a unified call set, along with several other tools, of 10,884 SVs per human on average [77]. In cats, the average number of SVs per individual was 4 times higher than in humans, with 44,990 SVs per cat, suggesting the total number of SVs in cats is likely inflated. However, the majority of the SVs were at population frequencies below 0.5, ruling out poor reference assembly as a contributing factor. Instead, the difference in SV count between cats and humans is likely due to sample specific factors. For example, the majority of samples were sequenced using two separate libraries of 350 bp and 550 bp insert sizes (**S2 Data**). Despite the potentially high number of false positive SVs, increased SV sensitivity was useful for trait discovery. The deletion associated with dwarfism was only found in the Delly2 call set. If SV filtering were more stringent, such as requiring SVs to be called by both callers, the feline dwarfism SV may not have otherwise been detected. Ultimately, these results highlight the importance of high sensitivity for initial SV discovery and the use of highly specific molecular techniques for downstream validation of candidate causative SVs.

The improved contiguity of Felis_catus_9.0 was particularly beneficial for identifying a causative SV for feline dwarfism. In humans, approximately 70% of cases are caused by spontaneous mutations in fibroblast growth factor 3 resulting in achondroplasia or a milder form of the condition known as hypochondroplasia [78, 79]. The domestic cat is one of the few species with an autosomal dominant mode of inheritance for dwarfism that does not have other syndromic features. It can therefore provide as a strong model for hypochondroplasia. Previous GWAS and linkage analyses suggested a critical region of association for feline disproportionate dwarfism on cat chromosome B1 that spanned 5.2 Mb [27]. Within the critical region, SV analysis identified a 3.3 kb deletion that had removed the final exon of *UGDH*, which was replaced by a 106 bp insertion with partial homology to *UGDH* exon 8, suggesting a potential duplication event. Importantly, *UGDH* likely plays a role in proteoglycan synthesis in articular chondrocytes, as osteoarthritic human and rat cartilage samples have revealed reduced UGDH protein expression was associated with a disease state [33]. Similarly, in dwarf cat samples, histology of the distal radius showed irregularity of the chondrocyte organization and proteoglycan depletion. Collectively, results suggest a disease model of reduced proteoglycan synthesis in dwarf cat chondrocytes caused by loss of function of *UGDH* resulting in abnormal growth in the long bones of dwarf cats.

In humans and model organisms, *UGDH* mutants have led to various alternate developmental outcomes. One example, is a homozygous missense variant, NM_003359.4(**UDGH**):c.950G>A (p.Arg317Gln), which was observed in humans as a causative variant for a patient experiencing a range of distinct clinical features including global developmental delay, axial hypotonia, bilateral undescended testis, and subtle dysmorphic features [80]. The mutation was located in exon 8 and belongs to the same exon 8 region in cats that appeared to be duplicated as part of the feline dwarfism SV, a region of the protein known as the central domain. Other more severe LoF mutations have also been identified in *UGDH* in humans, causing recessive developmental epileptic encephalopathy [81]. Almost all of these cases were compound heterozygotes with healthy parents, indicating a single functional copy of *UGDH* is sufficient for healthy development in humans. In other species, homozygous knockouts are embryonic lethal [82, 83], which seems to be consistent with observed inheritance patterns in cats. The ultimate impact of the *UGDH* SV in dwarf cats and its specific role in causing the dwarfism phenotype is difficult to determine. Clearly, the activity from the wildtype allele in cats is sufficient to prevent the onset of severe developmental delay and encephalopathy observed in humans, however, in other species, heterozygous LoF mutations have not been associated with similar phenotypes as observed in cats. Two potential outcomes from the analysis are 1) the SV itself causes a gain of function for *UGDH* with activity specific to growth of long bones, or 2) feline *UGDH* plays a unique feline specific role in bone growth, where loss of function of this gene causes feline dwarfism. Since the feline *UGDH* SV contains a complete deletion of the final exon, the coding sequence of the mutant transcript is difficult to predict, making it impossible to infer any potential functional impacts. However, successful sequencing of the cDNA of the mutant allele should help overcome this obstacle and lead to better inference of the functional impacts of the feline mutant allele. In addition, as a novel gene association with dwarfism, *UGDH* should also be screened for variants in undiagnosed human dwarf patients.

High-quality genomes are a prerequisite for unhindered computational experimentation. Felis_catus_9.0 is currently the most contiguous genome of a companion animal, with high accuracy and improved gene annotation that serves as a reference point for the discovery of genetic variation associated with many traits. This new genomic resource will provide a foundation for the future practice of genomic medicine in cats and for comparative analyses with other species.

## Methods

### Whole genome sequencing

The same genome reference inbred domestic cat, Cinnamon, the Abyssinian, was used for the long-read sequencing [84, 35]. High molecular weight DNA was isolated using a MagAttract HMW-DNA Kit (Qiagen, Germantown, MD) from cultured fibroblast cells according to the manufacturer’s protocol. Single molecule real-time (SMRT) sequencing was completed on the RSII and Sequel instruments (Pacific Biosciences, Menlo Park, CA).

### Genome assembly

All sequences (~72x total sequence coverage) were assembled with the fuzzy Bruijn graph algorithm, WTDBG (https://github.com/ruanjue/wtdbg), followed by collective raw read alignment using MINIMAP to the error-prone primary contigs [85]. As a result, contig coverage and graph topology were used to detect base errors that deviated from the majority haplotype branches that are due to long-read error (i.e. chimeric reads) or erroneous graph trajectories (i.e. repeats) as opposed to allelic structural variation, in which case, the sequence of one of the alleles is incorporated into the final consensus bases. As a final step to improve the consensus base quality of the assembly, from the same source DNA (Cinammon), short read sequences (150 bp) were generated from 400 bp fragment size TruSeq libraries to ~60X coverage on the Illumina HiSeqX instrument, which was then used to correct homozygous insertion, deletion and single base differences using PILON [86].

### Assembly scaffolding

To generate the first iteration of scaffolds from assembled contigs, the BioNano Irys technology was used to define order and orientation, as well as, detect chimeric contigs for automated breaks [87]. HMW-DNA in agar plugs was prepared from the same cultured fibroblast cell line using the BioNano recommended protocol for soft tissues, where using the IrysPrep Reagent Kit, a series of enzymatic reactions lysed cells, degraded protein and RNA, and added fluorescent labels to nicked sites. The nicked DNA fragments were labeled with ALEXA Fluor 546 dye and the DNA molecules were counter-stained with YOYO-1 dye. After which, the labeled DNA fragments were electrophoretically elongated and sized on a single IrysChip, with subsequent imaging and data processing to determine the size of each DNA fragment. Finally, a *de novo* assembly was performed by using all labeled fragments >150 kb to construct a whole-genome optical map with defined overlap patterns. Individual maps were clustered, scored for pairwise similarity, and Euclidian distance matrices were built. Manual refinements were then performed as previously described [87].

### Assembly QC

The scaffolded assembly was aligned to the latest cat linkage map [16] to detect incorrect linkage between and within scaffolds, as well as discontinuous translocation events that suggest contig chimerism. Following a genome-wide review of interchromosomal scaffold discrepancies with the linkage map, the sequence breakpoints were manually determined and the incorrect sequence linkages were separated. Also, to assess the assembly of expanded heterozygous loci ‘insertions’, the same reference DNA sequences (Illumina short read inserts 300 bp) were aligned to the chromosomes to detect homozygous deletions in the read alignments using Manta [88], a structural variant detection algorithm. In addition, the contigs sequences were aligned to the Felis_catus_8.0 using BLAT [89] at 99% identity and scored alignment insertion length at 0.5 to 50 kb length to further refine putatively falsely assembled heterozygous loci that when intersected with repeat tracks suggested either error in the assembly or inability to correctly delineate the repetitive copy.

### Chromosome builds

Upon correction and completion of the scaffold assembly, the genetic linkage map [16] was used to first order and orient all possible scaffolds by using the Chromonomer tool similarly to the previously reported default assembly parameter settings [90]. A final manual breakage of any remaining incorrect scaffold structure was made considering various alignment discordance metrics, including to the prior reference Felis_catus_8.0 that defined unexpected interchromosomal translocations and lastly paired end size discordance using alignments of BAC end sequences from the Cinnamon DNA source (http://ampliconexpress.com/bac-libraries/ite).

### Gene annotation

The Felis_catus_9.0 assembly was annotated using previously described NCBI [91, 92] and Ensembl [93] pipelines, that included masking of repeats prior to *ab initio* gene predictions and evidence-supported gene model building using RNA sequencing data [94]. RNA sequencing data of varied tissue types (https://www.ncbi.nlm.nih.gov/genome/annotation_euk/Felis_catus/104) was used to further improve gene model accuracy by alignment to nascent gene models that are necessary to delineate boundaries of untranslated regions as well as to identify genes not found through interspecific similarity evidence from other species.

### Characterizing feline sequence variation

Seventy-four cat WGSs from the 99 Lives Cat Genome Project were downloaded from the NCBI short read archive (SRA) affiliated with NCBI biosample and bioproject numbers (**S2 Data**). All sequences were produced with Illumina technology, on either an Illumina HiSeq 2500 or X Ten instrument using PCR-free libraries with insert lengths ranging from 350 bp to 550 bp, producing 100–150 bp paired-end reads. WGS data was processed using the Genome analysis toolkit (GATK) version 3.8 [95, 96]. BWA-MEM from Burrows-Wheeler Aligner version 0.7.17 was used to map reads to Felis_catus_9.0 (GCF_000181335.3) [97]. Picard tools version 2.1.1 (http://broadinstitute.github.io/picard/) was used to mark duplicate reads, and samtools version 1.7 [98] was used to sort, merge and index reads. Tools used from GATK 3.8 consisted of IndelRealigner and RealignerTargetCreator for indel realignment, BaseRecalibrator for base quality score recalibration (BQSR) [99], and HaplotypeCaller and GenotypeGVCFs for genotyping [100]. The variant database used for BQSR was built by first genotyping non-recalibrated BAMs and applying a strict set of filters to isolate high confidence variants. To determine the final variant call set, post BQSR, a less-strict GATK recommended set of filters, was used. All filtering options are outlined in supplementary material (**S8 Table**). The set of unrelated cats was determined using vcftools' relatedness2 function on SNP genotypes to estimate the kinship coefficient, ϕ, for each pair of cats [101]. First, related cats were identified as sharing potential sibling and parent-child relationships if ϕ > 0.15. Next, cats with the highest number of relatives were removed in an iterative fashion until no relationships with ϕ > 0.15 remained. To detect population structure among the sequenced cats, principal component analysis was conducted using SNPRelate version 1.16.0 in the R Statistical Software package. The SNV set generated after the appropriate quality control measures were used was further filtered for non-biallelic SNVs and sites displaying linkage disequilibrium (r2 threshold = 0.2) as implemented in the SNPRelate package.

### Measuring coding variant impacts on human disease genes

VCF summary statistics and allele counts were computed using various functions from vcftools [101] and vcflib (https://github.com/vcflib/vcflib). SNV impacts of synonymous, missense, and LoF were determined using Ensembl’s variant effect predictor (VEP) with annotations from Ensembl release 97 [29, 93]. Cat and human orthologs were identified using reciprocal best hits blast, where Ensembl 98 protein fasta sequences were used as queries. Using pLI for human genes obtained from gnomAD [58, 102], genes were assigned to constraint groups based on weak constraint (pLI < 0.1), moderate constraint (pLI > 0.1 and pLI < 0.9), and strong constraint (pLI > 0.9). The observed per kb SNV density, *Y*^*D*^, for each constraint group and SNV impact was calculated as, $Y_{GI}^{D}=X_{GI}/C_{G}\times 1000bp$, where *C* is the total length of the coding sequence and *X* is the number of SNVs within *C*. The subscript *I* refers to SNV impacts from the subset of all SNVs, *X*. The subscript *G* refers to the subset of either *C* or *X* that are found within a particular constraint group. For example, when *G* represents genes under weak constraint and *I* represents LoF SNVs, *X*_*GI*_ would be all LoF SNVs within the coding sequence of genes under weak constraint. The expected per kb SNV density, *E*^*D*^, for each constraint group and impact was calculated as, $E_{GI}^{D}=\frac{X_{G}(X_{I}÷X)}{C_{G}}\times 1000bp$. The 95% confidence intervals surrounding the expected per kb SNV densities were calculated from a random distribution generated by 10,000 permutations, where SNV impacts were shuffled randomly across variant sites. The observed singleton fraction, *Y*^*F*^, for each constraint group and impact was calculated as, $Y_{GI}^{F}=X_{GIP}/X_{GI}$, where the subscript *P* refers to SNVs identified as singletons. The expected singleton fraction was calculated as, $E_{B}^{F}=X_{P}/X$, where the 95% confidence intervals surrounding the expected singleton fractions were also calculated from a random distribution generated by 10,000 permutations. For each permutation, SNV MAFs were shuffled so variants were randomly assigned “singleton” status. Contrasts of SNV counts and singleton counts between constraint groups were captured using a Fisher test. For SNV counts, a contingency table was built for each SNV impact and pair of constraint groups. The table consisted of the number of nucleotides that belonged to a particular impact and the total number of nucleotides in the coding region that did not belong to that same impact type. For singleton counts, the contingency table consisted of the number of SNVs of a particular impact type that were singletons and the number of SNVs of that same impact type that were not. Fisher tests were performed using R.

### Structural variant identification and analysis

To discover SVs in the size range of <100 kb, aligned reads from all cats to Felis_catus_9.0 were used as input for the LUMPY [75] and Delly2 [74] SV callers. For LUMPY, the empirical insert size was determined using samtools and pairend_distro.py for each BAM. Discordant and split-reads extracted from paired-end data using SpeedSeq [103] were used as input along with each aligned BAM, minimum mapping threshold of 20, and empirical mean and standard deviation of insert size. Samples were called independently. SVTyper [103] was used to genotype each SV before merging all resulting VCFs using BCFtools [98]. For Delly2 [74], all SVs were called for individual BAMs independently, then merged into a single BCF. For each sample, variants were then re-called using the merged results from all samples. The individual re-called VCFs were then merged into a single file using BCFtools. Given the poor resolution of LUMPY for small insertions, only the Delly2 calls were considered and variants were required to be found in more than 2 individuals. A convergence of calls was determined by reciprocal overlap of 50% of the defined breakpoint in each caller as our final set. SVs were annotated using SnpEff [104], which was used to count gene region intersects. SVs were considered exonic if they were annotated as exon_region, frameshift, start_lost, or stop_gained.

### Disease variant discovery for dwarfism

To discover causal variants associated with dwarfism, three unrelated affected cats with disproportionate dwarfism from the 99 Lives genome dataset were examined for SVs. Identified SVs were considered causal candidates if they were, 1) concordant with affection status and an autosomal dominant inheritance pattern, 2) and located within the ~5.2 Mb dwarfism critical region located on cat chromosome B1:170,786,914–175,975,857 [27]. After initial identification, candidate variants were prioritized according to their predicted impact on protein coding genes. High priority candidate SVs were further characterized manually in affected individuals using the integrated genomics viewer (IGV) [105]. STIX (structural variant index) was used to validate candidate SVs by searching BAM files for discordant read-pairs that overlapped candidate SV regions (https://github.com/ryanlayer/stix). After manual characterization, SV breakpoints were validated with PCR amplification and Sanger sequencing. For further genotyping of candidate SVs, all sample collection and cat studies were conducted in accordance with an approved University of California, Davis Institutional Animal Care and Use protocols 11977, 15117, and 16691 and University of Missouri protocols 7808 and 8292. DNA samples from dwarf and normal cats were genotyped for the candidate SV identified in the three sequenced dwarfism cats [27]. PCR primers were designed using the known SV sequence breakpoints (**S9A Fig**). For validation, PCR amplification products were sanger sequenced and compared against Felis_catus_9.0. For screening, all samples from previous linkage and GWAS studies [27] were genotyped using the primers, UGDH_mid_F, UGDH_del_R, and UGDH_dn_R (**S9 Table**) in a single reaction. PCR products were separated by gel electrophoresis (80V, 90 minutes) in 1.25% (w/v) agarose in 1X TAE. A 622 bp amplicon was expected from the normal allele and a 481 bp amplicon from the affected allele (**S6 Fig**).

### Histological characterization of dwarf cat growth plates

Cat owners voluntarily submitted cadavers of stillborn dwarf and musculoskeletally normal kittens (7 and 2 respectively, dying from natural causes) via overnight shipment on ice. Tissues were not in a suitable state for RNA-seq analysis. Distal radius including physis (epiphyseal plate) were collected and fixed in 10% neutral buffered formalin. These tissues were decalcified in 10% EDTA solution. After complete decalcification the tissues were dehydrated with gradually increasing concentrations of ethanol and embedded in paraffin. Frontal sections of distal radial tissues were cut to 6 μm and mounted onto microscope slides. The samples were then dewaxed, rehydrated, and stained with hematoxylin and eosin to evaluate the tissue structure and cell morphology. The sections were also stained with toluidine blue to determine the distribution and quantity of proteoglycans. These samples were subjectively assessed for chondrocyte and tissue morphology and growth plate architecture by a pathologist (KK) who was blinded to the sample information.

## Declarations

### Ethics approval and consent to participate

All sample collection and cat studies were conducted in accordance with an approved University of California, Davis Institutional Animal Care and Use protocols 11977, 15117, and 16691 and University of Missouri protocols 7808 and 8292.

## Supporting information

S1 Table Representative annotation measures for assembled carnivore genomes. (DOCX)

S2 Table Repeat masking of 10 largest repeat classes in Felis_catus_9.0. (DOCX)

S3 Table Variant calling summary statistics. (DOCX)

S4 Table Truth sensitivity of SNV call set. (DOCX)

S5 Table SNV classification by minor allele frequency in domestic cats. (DOCX)

S6 Table Values used for pairwise fisher tests of association with gene constraint. (DOCX)

S7 Table Feline LoF singletons in human genes under strong constraint. (DOCX)

S8 Table GATK variant filtering criteria. (DOCX)

S9 Table PCR Primers for the genotyping of feline disproportionate dwarfism. (DOCX)

S1 Fig Repeat coverage and fragment length distribution of LINE/L1 elements and SINE/tRNA elemetns. Inset focusses on full length L1 fragments, which only make up a small fraction of all L1s.
(TIFF)

S2 Fig RNA-seq read coverage of singleton LoF SNVs with overlapping Ensembl and NCBI gene annotations. The SNV position is highlighted in each figure panel in blue. Images were generated using NCBI’s graphics option through the Nucleotide database.
(TIFF)

S3 Fig RNA-seq read coverage of singleton LoF SNVs with non-overlapping Ensembl and NCBI gene annotations. The SNV position is highlighted in each figure panel in blue. Images were generated using NCBI’s graphics option through the Nucleotide database.
(TIFF)

S4 Fig LoF SNVs per individual in all genes and strong constrained genes. Dotted line shows the mean value, which is also stated above along with standard deviation in braces.
(TIFF)

S5 Fig Mean LoF SNVs per individual grouped by minor allele frequency in all genes and strong constrained genes. Error bars represent 1 standard deviation.
(TIFF)

S6 Fig Number of SVs found in breed cats and random bred cats. (TIFF)

S7 Fig SV frequency per chromosome. (TIFF)

S8 Fig Discordant reads overlapping *UGDH* are unique to unrelated affected cats. Unrelated affected cats are felCat.19050.Mouse, felCat.19060.Gwenivere, and felCat.19067.Princess. felCat.17799.Cali is an unaffected normal control cat. Coverage across the control cat is relatively uniform, while the affected cats show decreased coverage over the final exon of UGDH marking a heterozygous deletion. Discordant reads that span beyond the deletion show sequence into the deleted region shares homology with *UGDH* exon 8.
(TIFF)

S9 Fig Breakpoints validated for dwarfism SV in *UGDH*. **(a)** Arrows represent individual primers and predicted band sizes. Gel photos show no template control (C), normal sample (N), affected sample (A), and 100 bp PLUS™ DNA Ladder (Gold Biotechnology, Inc., St. Louis, MO) (L). Ladder sizes are shown to the right of each gel image in bp. Above each gel image is the primers that were used to generate the band in each sample. Band sizes were consistent with predicted breakpoint lengths. Hatched square is 49 bp segment that shares homology with exon 8, it is consistent with a duplication and insertion into deleted region. Yellow boxes represent sequence of unknown origin found in affected allele. The deletion is absent from the affected allele, allowing primer four to produce an amplicon with primer 2. All dwarf samples analyzed were heterozygous for the affected allele. Primers 1 –UDGH_mid_R, 2 –UDGH_mid_F, 3 –UDGH_del_R, 4 –UDGH_down_R, 5 –UDGH_up_F. **(b)** Full gel image for primer combinations described in A.
(TIFF)

S10 Fig Histology of control and dwarf cartilage plates. H&E and toluidine blue histologic samples of the distal radius epiphyseal cartilage plate from a normal neonatal kitten and age-matched dwarf kittens. For normal control kitten, H&E shows chondrocytes in the growth plate exhibit a regular columnar arrangement and are organized into a zone of reserve cells, a zone of proliferation and a zone of hypertrophy and a zone of provisional calcification. For the same kitten toluidine blue stain shows physeal cartilage contains abundant proteoglycan as shown by its metachromasia. In dwarf samples H&E staining consistently shows chondrocytes in the growth plate exhibit an irregular columnar arrangement. For four of the six dwarf samples, toluidine blue staining shows by its metachromasia that dwarf cat physeal cartilage contains lessor amounts of proteoglycans.
(TIFF)

S1 Data Felis_catus_9.0 chromosomal order and orientation compared to linkage map generated by Li *et al* (2016). (PDF)

S2 Data Summary of NCBI gene annotation changes between Felis_catus_9.0 and Felis_catus_8.0. Each row consists of a pair of current (Felis_catus 9.0) and previous (Felis_catus 8.0) features that were categorized based on these scores: reciprocal best matches, and changes in attributes, such as completeness. Worksheet 1 is the report of all genes, Worksheet 2 is only protein-coding genes and Worksheet 3 is protein-coding genes with duplicate annotations removed. This cumulative report of gene annotation quality is provided by NCBI at: https://www.ncbi.nlm.nih.gov/genome/annotation_euk/Felis_catus/104/#AnnotationComparisonStats.
(XLSX)

S3 Data Sample specific information and sequencing statistics for all cats used in the analysis. (XLSX)

S4 Data SNV positions from the feline 63K genotyping array mapped to Felis_catus_9.0. (XLSX)

S5 Data Sample specific SNV calls and statistics. (XLSX)

S6 Data Structural variant calls. “L1” is for SVs found only using LUMPY, “D1” is for SVs found only using Delly2, “2” is SVs found using both callers, and “0” is for when no SV is found using either caller.
(CSV)

S7 Data Structural variant impact on protein coding genes. (XLSX)

S8 Data Genotyping results for dwarfism *UGDH* structural variant. “Non-standard” is the breed terminology used to represent individuals with normal sized limbs, while “Standard” is used to represent dwarf cats. Genotype “ref” is for individuals whose genotype is the same as the reference, while “het” is used for individuals heterozygous for the candidate SV. No individuals homozygous for the SV were observed.
(CSV)

## References

[1] Mauler DA, Gandolfi B, Reinero CR, O'Brien DP, Spooner JL, Lyons LA, et al Precision Medicine in Cats: Novel Niemann-Pick Type C1 Diagnosed by Whole-Genome Sequencing. J Vet Intern Med. 2017;31(2):539–44. 10.1111/jvim.14599 28233346 PMC5354023

[2] Fang H, Wu Y, Yang H, Yoon M, Jiménez-Barrón LT, Mittelman D, et al Whole genome sequencing of one complex pedigree illustrates challenges with genomic medicine. BMC medical genomics. 2017;10(1):10 10.1186/s12920-017-0246-5 28228131 PMC5322674

[3] Wise AL, Manolio TA, Mensah GA, Peterson JF, Roden DM, Tamburro C, et al Genomic medicine for undiagnosed diseases. The Lancet. 2019. 10.1016/S0140-6736(19)31274-7 PMC6709871 31395441

[4] Shendure J, Findlay GM, Snyder MW. Genomic medicine–progress, pitfalls, and promise. Cell. 2019;177(1):45–57. 10.1016/j.cell.2019.02.003 30901547 PMC6531313

[5] Moses L, Niemi S, Karlsson E. Pet genomics medicine runs wild. Nature Publishing Group; 2018. 10.1038/d41586-018-05771-0 30046086

[6] Nicholas FW. Online Mendelian Inheritance in Animals (OMIA): a comparative knowledgebase of genetic disorders and other familial traits in non-laboratory animals. Nucleic acids research. 2003;31(1):275–7. 10.1093/nar/gkg074 12520001 PMC165521

[7] Online Mendelian Inheritance in Animals (OMIA). Sydney School of Veterinary Science, 03/12/2019. World Wide Web URL: http://omia.org/. Available from: http://omia.org/.

[8] Lyons LA. DNA mutations of the cat: the good, the bad and the ugly. J Feline Med Surg. 2015;17(3):203–19. Epub 2015/02/24. 10.1177/1098612X15571878 . 25701860 PMC11148888

[9] Kittleson MD, Meurs KM, Harris SP. The genetic basis of hypertrophic cardiomyopathy in cats and humans. J Vet Cardiol. 2015;17 Suppl 1:S53–73. Epub 2016/01/19. 10.1016/j.jvc.2015.03.001 26776594 PMC5909964

[10] Menotti-Raymond M, David VA, Schäffer AA, Stephens R, Wells D, Kumar-Singh R, et al Mutation in CEP290 discovered for cat model of human retinal degeneration. Journal of Heredity. 2007;98(3):211–20. 10.1093/jhered/esm019 17507457

[11] Lyons LA, Biller DS, Erdman CA, Lipinski MJ, Young AE, Roe BA, et al Feline polycystic kidney disease mutation identified in PKD1. J Am Soc Nephrol. 2004;15(10):2548–55. Epub 2004/10/07. 10.1097/01.ASN.0000141776.38527.BB . 15466259

[12] Wang P, Mazrier H, Caverly Rae J, Raj K, Giger U. A GNPTAB nonsense variant is associated with feline mucolipidosis II (I-cell disease). BMC Vet Res. 2018;14(1):416 Epub 2018/12/29. 10.1186/s12917-018-1728-1 30591066 PMC6307278

[13] Spycher M, Bauer A, Jagannathan V, Frizzi M, De Lucia M, Leeb T. A frameshift variant in the COL5A1 gene in a cat with Ehlers-Danlos syndrome. Anim Genet. 2018;49(6):641–4. Epub 2018/09/25. 10.1111/age.12727 . 30246406

[14] Jaffey JA, Reading NS, Giger U, Abdulmalik O, Buckley RM, Johnstone S, et al Clinical, metabolic, and genetic characterization of hereditary methemoglobinemia caused by cytochrome b5 reductase deficiency in cats. Journal of veterinary internal medicine. 2019. 10.1111/jvim.15637 PMC6872605 31650629

[15] Hug P, Kern P, Jagannathan V, Leeb T. A TAC3 Missense Variant in a Domestic Shorthair Cat with Testicular Hypoplasia and Persistent Primary Dentition. Genes. 2019;10(10):806. 10.3390/genes10100806 PMC6826659 31615056

[16] Li G, Hillier LW, Grahn RA, Zimin AV, David VA, Menotti-Raymond M, et al A High-Resolution SNP Array-Based Linkage Map Anchors a New Domestic Cat Draft Genome Assembly and Provides Detailed Patterns of Recombination. G3 (Bethesda). 2016;6(6):1607–16. 10.1534/g3.116.028746 27172201 PMC4889657

[17] Low WY, Tearle R, Bickhart DM, Rosen BD, Kingan SB, Swale T, et al Chromosome-level assembly of the water buffalo genome surpasses human and goat genomes in sequence contiguity. Nat Commun. 2019;10(1):260 Epub 2019/01/18. 10.1038/s41467-018-08260-0 30651564 PMC6335429

[18] Ananthasayanam S, Kothandaraman H., Nayee N, Saha S., Baghel D.S., Gopalakrishnan K., Peddamma S., Singh R.B., Schatz M. First near complete haplotype phased genome assembly of River buffalo (Bubalus bubalis). bioRxiv. 2019;(April 26).

[19] Gordon D, Huddleston J, Chaisson MJ, Hill CM, Kronenberg ZN, Munson KM, et al Long-read sequence assembly of the gorilla genome. Science. 2016;352(6281):aae0344. Epub 2016/04/02. 10.1126/science.aae0344 27034376 PMC4920363

[20] Bickhart DM, Rosen BD, Koren S, Sayre BL, Hastie AR, Chan S, et al Single-molecule sequencing and chromatin conformation capture enable de novo reference assembly of the domestic goat genome. Nat Genet. 2017;49(4):643–50. Epub 2017/03/07. 10.1038/ng.3802 28263316 PMC5909822

[21] Ontiveros ES, Ueda Y, Harris SP, Stern JA, 99 Lives Consortium. Precision medicine validation: identifying the MYBPC 3 A31P variant with whole-genome sequencing in two Maine Coon cats with hypertrophic cardiomyopathy. Journal of feline medicine and surgery. 2018:1098612X18816460. 10.1177/1098612X18816460 PMC10814263 30558461

[22] Oh A, Pearce JW, Gandolfi B, Creighton EK, Suedmeyer WK, Selig M, et al Early-onset progressive retinal atrophy associated with an IQCB1 variant in African black-footed cats (Felis nigripes). Scientific reports. 2017;7:43918 10.1038/srep43918 28322220 PMC5359545

[23] Aberdein D, Munday JS, Gandolfi B, Dittmer KE, Malik R, Garrick DJ, et al A FAS-ligand variant associated with autoimmune lymphoproliferative syndrome in cats. Mamm Genome. 2017;28(1–2):47–55. Epub 2016/10/23. 10.1007/s00335-016-9668-1 . 27770190

[24] Genova F, Longeri M, Lyons LA, Bagnato A, 99 Lives Consortium, Strillacci MG. First genome-wide CNV mapping in FELIS CATUS using next generation sequencing data. BMC Genomics. 2018;19(1):895 Epub 2018/12/12. 10.1186/s12864-018-5297-2 30526495 PMC6288940

[25] Smit A, Hubley R, Green P. 2013–2015. RepeatMasker Open-4.0. 2013.

[26] Gandolfi B, Alhaddad H, Abdi M, Bach LH, Creighton EK, Davis BW, et al Applications and efficiencies of the first cat 63K DNA array. Sci Rep. 2018;8(1):7024 Epub 2018/05/08. 10.1038/s41598-018-25438-0 29728693 PMC5935720

[27] Lyons LA, Fox DB, Chesney KL, Britt LG, Buckley RM, Coates JR, et al Localization of a feline autosomal dominant dwarfism locus: a novel model of chondrodysplasia. bioRxiv. 2019:687210.

[28] Lipinski MJ, Froenicke L, Baysac KC, Billings NC, Leutenegger CM, Levy AM, et al The ascent of cat breeds: genetic evaluations of breeds and worldwide random-bred populations. Genomics. 2008;91(1):12–21. Epub 2007/12/07. 10.1016/j.ygeno.2007.10.009 18060738 PMC2267438

[29] McLaren W, Gil L, Hunt SE, Riat HS, Ritchie GR, Thormann A, et al The Ensembl Variant Effect Predictor. Genome Biol. 2016;17(1):122 Epub 2016/06/09. 10.1186/s13059-016-0974-4 27268795 PMC4893825

[30] Dewey FE, Murray MF, Overton JD, Habegger L, Leader JB, Fetterolf SN, et al Distribution and clinical impact of functional variants in 50,726 whole-exome sequences from the DiscovEHR study. Science. 2016;354(6319):aaf6814. 10.1126/science.aaf6814 28008009

[31] Yeh CH, Bellon M, Nicot C. FBXW7: a critical tumor suppressor of human cancers. Mol Cancer. 2018;17(1):115 Epub 2018/08/09. 10.1186/s12943-018-0857-2 30086763 PMC6081812

[32] TICA. Munchkin 2015 [October 26, 2015].

[33] Wen Y, Li J, Wang L, Tie K, Magdalou J, Chen L, et al UDP-glucose dehydrogenase modulates proteoglycan synthesis in articular chondrocytes: its possible involvement and regulation in osteoarthritis. Arthritis Res Ther. 2014;16(6):484 Epub 2014/12/04. 10.1186/s13075-014-0484-2 25465897 PMC4298080

[34] Clarkin CE, Allen S, Kuiper NJ, Wheeler BT, Wheeler-Jones CP, Pitsillides AA. Regulation of UDP-glucose dehydrogenase is sufficient to modulate hyaluronan production and release, control sulfated GAG synthesis, and promote chondrogenesis. J Cell Physiol. 2011;226(3):749–61. Epub 2010/08/19. 10.1002/jcp.22393 . 20717929

[35] Montague MJ, Li G, Gandolfi B, Khan R, Aken BL, Searle SM, et al Comparative analysis of the domestic cat genome reveals genetic signatures underlying feline biology and domestication. Proc Natl Acad Sci U S A. 2014;111(48):17230–5. 10.1073/pnas.1410083111 25385592 PMC4260561

[36] Yu Y, Grahn RA, Lyons LA. Mocha tyrosinase variant: a new flavour of cat coat coloration. Anim Genet. 2019;50(2):182–6. Epub 2019/02/05. 10.1111/age.12765 . 30716167 PMC6590430

[37] Yu Y, Shumway KL, Matheson JS, Edwards ME, Kline TL, Lyons LA. Kidney and cystic volume imaging for disease presentation and progression in the cat autosomal dominant polycystic kidney disease large animal model. BMC Nephrology. 2019;20(1):259 10.1186/s12882-019-1448-1 31299928 PMC6625046

[38] Daetwyler HD, Capitan A, Pausch H, Stothard P, van Binsbergen R, Brondum RF, et al Whole-genome sequencing of 234 bulls facilitates mapping of monogenic and complex traits in cattle. Nat Genet. 2014;46(8):858–65. Epub 2014/07/16. 10.1038/ng.3034 . 25017103

[39] Bai B, Zhao WM, Tang BX, Wang YQ, Wang L, Zhang Z, et al DoGSD: the dog and wolf genome SNP database. Nucleic Acids Res. 2015;43(Database issue):D777–83. Epub 2014/11/19. 10.1093/nar/gku1174 25404132 PMC4383968

[40] Atanur SS, Diaz AG, Maratou K, Sarkis A, Rotival M, Game L, et al Genome sequencing reveals loci under artificial selection that underlie disease phenotypes in the laboratory rat. Cell. 2013;154(3):691–703. Epub 2013/07/31. 10.1016/j.cell.2013.06.040 23890820 PMC3732391

[41] Hermsen R, de Ligt J, Spee W, Blokzijl F, Schafer S, Adami E, et al Genomic landscape of rat strain and substrain variation. BMC Genomics. 2015;16:357 Epub 2015/05/07. 10.1186/s12864-015-1594-1 25943489 PMC4422378

[42] Teng H, Zhang Y, Shi C, Mao F, Cai W, Lu L, et al Population Genomics Reveals Speciation and Introgression between Brown Norway Rats and Their Sibling Species. Mol Biol Evol. 2017;34(9):2214–28. Epub 2017/05/10. 10.1093/molbev/msx157 28482038 PMC5850741

[43] Yang J, Li WR, Lv FH, He SG, Tian SL, Peng WF, et al Whole-Genome Sequencing of Native Sheep Provides Insights into Rapid Adaptations to Extreme Environments. Mol Biol Evol. 2016;33(10):2576–92. Epub 2016/07/13. 10.1093/molbev/msw129 27401233 PMC5026255

[44] Chen ZH, Zhang M, Lv FH, Ren X, Li WR, Liu MJ, et al Contrasting Patterns of Genomic Diversity Reveal Accelerated Genetic Drift but Reduced Directional Selection on X-Chromosome in Wild and Domestic Sheep Species. Genome Biol Evol. 2018;10(5):1282–97. Epub 2018/05/24. 10.1093/gbe/evy085 29790980 PMC5963296

[45] Choi JW, Chung WH, Lee KT, Cho ES, Lee SW, Choi BH, et al Whole-genome resequencing analyses of five pig breeds, including Korean wild and native, and three European origin breeds. DNA Res. 2015;22(4):259–67. Epub 2015/06/29. 10.1093/dnares/dsv011 26117497 PMC4535618

[46] Jagannathan V, Gerber V, Rieder S, Tetens J, Thaller G, Drogemuller C, et al Comprehensive characterization of horse genome variation by whole-genome sequencing of 88 horses. Anim Genet. 2019;50(1):74–7. Epub 2018/12/14. 10.1111/age.12753 . 30525216

[47] Bimber BN, Ramakrishnan R, Cervera-Juanes R, Madhira R, Peterson SM, Norgren RB Jr, et al Whole genome sequencing predicts novel human disease models in rhesus macaques. Genomics. 2017;109(3–4):214–20. 10.1016/j.ygeno.2017.04.001 28438488 PMC5513488

[48] Xue C, Raveendran M, Harris RA, Fawcett GL, Liu X, White S, et al The population genomics of rhesus macaques (Macaca mulatta) based on whole-genome sequences. Genome Res. 2016;26(12):1651–62. Epub 2016/12/10. 10.1101/gr.204255.116 27934697 PMC5131817

[49] The 1000 Genomes Project Consortium, Auton A, Brooks LD, Durbin RM, Garrison EP, Kang HM, et al A global reference for human genetic variation. Nature. 2015;526(7571):68–74. Epub 2015/10/04. 10.1038/nature15393 26432245 PMC4750478

[50] Ottoni C, Van Neer W, De Cupere B, Daligault J, Guimaraes S, Peters J, et al The palaeogenetics of cat dispersal in the ancient world. Nature Ecology & Evolution. 2017;1(7):0139.

[51] Vigne J-D, Guilaine J, Debue K, Haye L, Gérard P. Early taming of the cat in Cyprus. Science. 2004;304(5668):259-. 10.1126/science.1095335 15073370

[52] Van Neer W, Linseele V, Friedman R, De Cupere B. More evidence for cat taming at the Predynastic elite cemetery of Hierakonpolis (Upper Egypt). Journal of Archaeological Science. 2014;45:103–11.

[53] Driscoll CA, Menotti-Raymond M, Roca AL, Hupe K, Johnson WE, Geffen E, et al The Near Eastern origin of cat domestication. Science. 2007;317(5837):519–23. Epub 2007/06/30. 10.1126/science.1139518 17600185 PMC5612713

[54] Kurushima JD, Lipinski MJ, Gandolfi B, Froenicke L, Grahn JC, Grahn RA, et al Variation of cats under domestication: genetic assignment of domestic cats to breeds and worldwide random-bred populations. Anim Genet. 2013;44(3):311–24. Epub 2012/11/23. 10.1111/age.12008 23171373 PMC3594446

[55] Festing MF. Inbred strains should replace outbred stocks in toxicology, safety testing, and drug development. Toxicologic pathology. 2010;38(5):681–90. 10.1177/0192623310373776 20562325

[56] Sundaram L, Gao H, Padigepati SR, McRae JF, Li Y, Kosmicki JA, et al Predicting the clinical impact of human mutation with deep neural networks. Nat Genet. 2018;50(8):1161–70. Epub 2018/07/25. 10.1038/s41588-018-0167-z 30038395 PMC6237276

[57] Frazer KA, Murray SS, Schork NJ, Topol EJ. Human genetic variation and its contribution to complex traits. Nature Reviews Genetics. 2009;10(4):241 10.1038/nrg2554 19293820

[58] Lek M, Karczewski KJ, Minikel EV, Samocha KE, Banks E, Fennell T, et al Analysis of protein-coding genetic variation in 60,706 humans. Nature. 2016;536(7616):285–91. Epub 2016/08/19. 10.1038/nature19057 27535533 PMC5018207

[59] Gudbjartsson DF, Helgason H, Gudjonsson SA, Zink F, Oddson A, Gylfason A, et al Large-scale whole-genome sequencing of the Icelandic population. Nat Genet. 2015;47(5):435–44. Epub 2015/03/26. 10.1038/ng.3247 . 25807286

[60] The 1000 Genomes Project Consortium, Abecasis GR, Altshuler D, Auton A, Brooks LD, Durbin RM, et al A map of human genome variation from population-scale sequencing. Nature. 2010;467(7319):1061–73. Epub 2010/10/29. 10.1038/nature09534 20981092 PMC3042601

[61] Gabor L, Malik R, Canfield P. Clinical and anatomical features of lymphosarcoma in 118 cats. Australian Veterinary Journal. 1998;76(11):725–32. 10.1111/j.1751-0813.1998.tb12300.x 9862061

[62] Louwerens M, London CA, Pedersen NC, Lyons LA. Feline lymphoma in the post-feline leukemia virus era. J Vet Intern Med. 2005;19(3):329–35. Epub 2005/06/16. 10.1892/0891-6640(2005)19[329:flitpl]2.0.co;2 . 15954547

[63] Fabrizio F, Calam AE, Dobson JM, Middleton SA, Murphy S, Taylor SS, et al Feline mediastinal lymphoma: a retrospective study of signalment, retroviral status, response to chemotherapy and prognostic indicators. J Feline Med Surg. 2014;16(8):637–44. Epub 2013/12/25. 10.1177/1098612X13516621 . 24366846 PMC11164164

[64] Tetzlaff MT, Yu W, Li M, Zhang P, Finegold M, Mahon K, et al Defective cardiovascular development and elevated cyclin E and Notch proteins in mice lacking the Fbw7 F-box protein. Proc Natl Acad Sci U S A. 2004;101(10):3338–45. Epub 2004/02/10. 10.1073/pnas.0307875101 14766969 PMC373463

[65] Tsunematsu R, Nakayama K, Oike Y, Nishiyama M, Ishida N, Hatakeyama S, et al Mouse Fbw7/Sel-10/Cdc4 is required for notch degradation during vascular development. J Biol Chem. 2004;279(10):9417–23. Epub 2003/12/16. 10.1074/jbc.M312337200 . 14672936

[66] Mao JH, Perez-Losada J, Wu D, Delrosario R, Tsunematsu R, Nakayama KI, et al Fbxw7/Cdc4 is a p53-dependent, haploinsufficient tumour suppressor gene. Nature. 2004;432(7018):775–9. Epub 2004/12/14. 10.1038/nature03155 . 15592418

[67] Perez-Losada J, Wu D, DelRosario R, Balmain A, Mao JH. Allele-specific deletions in mouse tumors identify Fbxw7 as germline modifier of tumor susceptibility. PLoS One. 2012;7(2):e31301 Epub 2012/02/22. 10.1371/journal.pone.0031301 22348067 PMC3278431

[68] Maser RS, Choudhury B, Campbell PJ, Feng B, Wong KK, Protopopov A, et al Chromosomally unstable mouse tumours have genomic alterations similar to diverse human cancers. Nature. 2007;447(7147):966–71. Epub 2007/05/23. 10.1038/nature05886 17515920 PMC2714968

[69] Roversi G, Picinelli C, Bestetti I, Crippa M, Perotti D, Ciceri S, et al Constitutional de novo deletion of the FBXW7 gene in a patient with focal segmental glomerulosclerosis and multiple primitive tumors. Sci Rep. 2015;5:15454 Epub 2015/10/21. 10.1038/srep15454 26482194 PMC4612309

[70] Mahamdallie S, Yost S, Poyastro-Pearson E, Holt E, Zachariou A, Seal S, et al Identification of new Wilms tumour predisposition genes: an exome sequencing study. Lancet Child Adolesc Health. 2019;3(5):322–31. Epub 2019/03/20. 10.1016/S2352-4642(19)30018-5 30885698 PMC6472290

[71] Havrilla JM, Pedersen BS, Layer RM, Quinlan AR. A map of constrained coding regions in the human genome. 10.1038/s41588-018-0294-6 PMC6589356 30531870

[72] Abyzov A, Urban AE, Snyder M, Gerstein M. CNVnator: an approach to discover, genotype, and characterize typical and atypical CNVs from family and population genome sequencing. Genome Res. 2011;21(6):974–84. Epub 2011/02/18. 10.1101/gr.114876.110 21324876 PMC3106330

[73] Klambauer G, Schwarzbauer K, Mayr A, Clevert DA, Mitterecker A, Bodenhofer U, et al cn.MOPS: mixture of Poissons for discovering copy number variations in next-generation sequencing data with a low false discovery rate. Nucleic Acids Res. 2012;40(9):e69 Epub 2012/02/04. 10.1093/nar/gks003 22302147 PMC3351174

[74] Rausch T, Zichner T, Schlattl A, Stutz AM, Benes V, Korbel JO. DELLY: structural variant discovery by integrated paired-end and split-read analysis. Bioinformatics. 2012;28(18):i333–i9. 10.1093/bioinformatics/bts378 22962449 PMC3436805

[75] Layer RM, Chiang C, Quinlan AR, Hall IM. LUMPY: a probabilistic framework for structural variant discovery. Genome Biol. 2014;15(6):R84 10.1186/gb-2014-15-6-r84 24970577 PMC4197822

[76] Kosugi S, Momozawa Y, Liu X, Terao C, Kubo M, Kamatani Y. Comprehensive evaluation of structural variation detection algorithms for whole genome sequencing. Genome Biol. 2019;20(1):117 Epub 2019/06/05. 10.1186/s13059-019-1720-5 31159850 PMC6547561

[77] Chaisson MJP, Sanders AD, Zhao X, Malhotra A, Porubsky D, Rausch T, et al Multi-platform discovery of haplotype-resolved structural variation in human genomes. Nat Commun. 2019;10(1):1784 Epub 2019/04/18. 10.1038/s41467-018-08148-z 30992455 PMC6467913

[78] Horton WA, Hall JG, Hecht JT. Achondroplasia. Lancet. 2007;370(9582):162–72. Epub 2007/07/17. 10.1016/S0140-6736(07)61090-3 . 17630040

[79] Foldynova-Trantirkova S, Wilcox WR, Krejci P. Sixteen years and counting: the current understanding of fibroblast growth factor receptor 3 (FGFR3) signaling in skeletal dysplasias. Hum Mutat. 2012;33(1):29–41. Epub 2011/11/03. 10.1002/humu.21636 22045636 PMC3240715

[80] Alhamoudi KM, Bhat J, Nashabat M, Alharbi M, Alyafee Y, Asiri A, et al A Missense Mutation in the UGDH Gene Is Associated With Developmental Delay and Axial Hypotonia. Front Pediatr. 2020;8:71 Epub 2020/03/17. 10.3389/fped.2020.00071 32175296 PMC7056728

[81] Hengel H, Bosso-Lefevre C, Grady G, Szenker-Ravi E, Li H, Pierce S, et al Loss-of-function mutations in UDP-Glucose 6-Dehydrogenase cause recessive developmental epileptic encephalopathy. Nat Commun. 2020;11(1):595 Epub 2020/02/01. 10.1038/s41467-020-14360-7 32001716 PMC6992768

[82] Garcia-Garcia MJ, Anderson KV. Essential role of glycosaminoglycans in Fgf signaling during mouse gastrulation. Cell. 2003;114(6):727–37. Epub 2003/09/25. 10.1016/s0092-8674(03)00715-3 . 14505572

[83] Choksi SP, Babu D, Lau D, Yu X, Roy S. Systematic discovery of novel ciliary genes through functional genomics in the zebrafish. Development. 2014;141(17):3410–9. Epub 2014/08/21. 10.1242/dev.108209 25139857 PMC4199137

[84] Pontius JU, Mullikin JC, Smith DR, Agencourt Sequencing T, Lindblad-Toh K, Gnerre S, et al Initial sequence and comparative analysis of the cat genome. Genome Res. 2007;17(11):1675–89. Epub 2007/11/03. 10.1101/gr.6380007 17975172 PMC2045150

[85] Li H. Minimap and miniasm: fast mapping and de novo assembly for noisy long sequences. Bioinformatics. 2016;32(14):2103–10. Epub 2016/05/07. 10.1093/bioinformatics/btw152 27153593 PMC4937194

[86] Walker BJ, Abeel T, Shea T, Priest M, Abouelliel A, Sakthikumar S, et al Pilon: an integrated tool for comprehensive microbial variant detection and genome assembly improvement. PLoS One. 2014;9(11):e112963 10.1371/journal.pone.0112963 25409509 PMC4237348

[87] Lam ET, Hastie A, Lin C, Ehrlich D, Das SK, Austin MD, et al Genome mapping on nanochannel arrays for structural variation analysis and sequence assembly. Nature biotechnology. 2012;30(8):771 10.1038/nbt.2303 22797562 PMC3817024

[88] Chen X, Schulz-Trieglaff O, Shaw R, Barnes B, Schlesinger F, Kallberg M, et al Manta: rapid detection of structural variants and indels for germline and cancer sequencing applications. Bioinformatics. 2016;32(8):1220–2. 10.1093/bioinformatics/btv710 . 26647377

[89] Kent WJ. BLAT—the BLAST-like alignment tool. Genome Res. 2002;12(4):656–64. Epub 2002/04/05. 10.1101/gr.229202 11932250 PMC187518

[90] Small C, Bassham S, Catchen J, Amores A, Fuiten A, Brown R, et al The genome of the Gulf pipefish enables understanding of evolutionary innovations. Genome biology. 2016;17(1):258 10.1186/s13059-016-1126-6 27993155 PMC5168715

[91] Pruitt KD, Tatusova T, Brown GR, Maglott DR. NCBI Reference Sequences (RefSeq): current status, new features and genome annotation policy. Nucleic Acids Res. 2012;40(Database issue):D130–5. Epub 2011/11/29. 10.1093/nar/gkr1079 22121212 PMC3245008

[92] Thibaud-Nissen F, Souvorov A, Murphy T, DiCuccio M, Kitts P. Eukaryotic genome annotation pipeline. The NCBI Handbook [Internet] 2nd edition: National Center for Biotechnology Information (US); 2013.

[93] Zerbino DR, Achuthan P, Akanni W, Amode MR, Barrell D, Bhai J, et al Ensembl 2018. Nucleic Acids Res. 2018;46(D1):D754–D61. Epub 2017/11/21. 10.1093/nar/gkx1098 29155950 PMC5753206

[94] Visser M, Weber KL, Lyons LA, Rincon G, Boothe DM, Merritt DA. Identification and quantification of domestic feline cytochrome P450 transcriptome across multiple tissues. J Vet Pharmacol Ther. 2019;42(1):7–15. Epub 2018/09/02. 10.1111/jvp.12708 30171610 PMC6322962

[95] McKenna A, Hanna M, Banks E, Sivachenko A, Cibulskis K, Kernytsky A, et al The Genome Analysis Toolkit: a MapReduce framework for analyzing next-generation DNA sequencing data. Genome Res. 2010;20(9):1297–303. Epub 2010/07/21. 10.1101/gr.107524.110 20644199 PMC2928508

[96] Van der Auwera GA, Carneiro MO, Hartl C, Poplin R, Del Angel G, Levy-Moonshine A, et al From FastQ data to high confidence variant calls: the Genome Analysis Toolkit best practices pipeline. Curr Protoc Bioinformatics. 2013;43:11 0 1–33. Epub 2014/11/29. 10.1002/0471250953.bi1110s43 25431634 PMC4243306

[97] Li H. Aligning sequence reads, clone sequences and assembly contigs with BWA-MEM. arXiv preprint arXiv:13033997. 2013.

[98] Li H, Handsaker B, Wysoker A, Fennell T, Ruan J, Homer N, et al The Sequence Alignment/Map format and SAMtools. Bioinformatics. 2009;25(16):2078–9. Epub 2009/06/10. 10.1093/bioinformatics/btp352 19505943 PMC2723002

[99] DePristo MA, Banks E, Poplin R, Garimella KV, Maguire JR, Hartl C, et al A framework for variation discovery and genotyping using next-generation DNA sequencing data. Nature genetics. 2011;43(5):491 10.1038/ng.806 21478889 PMC3083463

[100] Poplin R, Ruano-Rubio V, DePristo MA, Fennell TJ, Carneiro MO, Van der Auwera GA, et al Scaling accurate genetic variant discovery to tens of thousands of samples. BioRxiv. 2018:201178.

[101] Danecek P, Auton A, Abecasis G, Albers CA, Banks E, DePristo MA, et al The variant call format and VCFtools. Bioinformatics. 2011;27(15):2156–8. 10.1093/bioinformatics/btr330 21653522 PMC3137218

[102] Karczewski KJ, Francioli LC, Tiao G, Cummings BB, Alföldi J, Wang Q, et al Variation across 141,456 human exomes and genomes reveals the spectrum of loss-of-function intolerance across human protein-coding genes. BioRxiv. 2019:531210.

[103] Chiang C, Layer RM, Faust GG, Lindberg MR, Rose DB, Garrison EP, et al SpeedSeq: ultra-fast personal genome analysis and interpretation. Nat Methods. 2015;12(10):966–8. 10.1038/nmeth.3505 26258291 PMC4589466

[104] Cingolani P, Platts A, Wang le L, Coon M, Nguyen T, Wang L, et al A program for annotating and predicting the effects of single nucleotide polymorphisms, SnpEff: SNPs in the genome of Drosophila melanogaster strain w1118; iso-2; iso-3. Fly (Austin). 2012;6(2):80–92. Epub 2012/06/26. 10.4161/fly.19695 22728672 PMC3679285

[105] Robinson JT, Thorvaldsdottir H, Winckler W, Guttman M, Lander ES, Getz G, et al Integrative genomics viewer. Nat Biotechnol. 2011;29(1):24–6. Epub 2011/01/12. 10.1038/nbt.1754 21221095 PMC3346182

