# Supplementary material for: A new domestic cat genome assembly based on long sequence reads empowers feline genomic medicine and identifies a novel gene for dwarfism
Source: PLoS Genet. 2020 Oct 22;16(10):e1008926. doi: 10.1371/journal.pgen.1008926 (PMC7581003; doi:10.1371/journal.pgen.1008926)
Supplement: S1 Table — (DOCX) [file pgen.1008926.s001.docx]

**Supplemental Table S1**. Representative annotation measures for assembled carnivore genomes^1^.

| **Species genus** | **Assembled version** | **Assembly level** | **Protein coding genes** | **Total ncRNA** | **mRNAs** | **Repeat masked (%)** |
| --- | --- | --- | --- | --- | --- | --- |
| *Felis catus* | Felis catus 9.0 | Chromosome | 19,748 | 11,679 | 54,713 | 42.72 |
| *Felis catus* | Felis catus 8.0 | Chromosome | 20,176 | 11,868 | 53,014 | 44.09 |
| *Canis lupus familiaris* | CanFam3.1 | Chromosome | 20,039 | 11,743 | 58,761 | 42.96 |
| *Canis lupus dingo* | ASM325472v1 | Scaffold | 20,248 | 12,981 | 62,946 | 42.86 |
| *Acinonyx jubatus* | Aci_jub_2 | Scaffold | 19,529 | 11,000 | 56,248 | 42.66 |
| *Ursus arctos horribilis* | ASM358476v1 | Scaffold | 19,848 | 7,061 | 43,155 | 41.01 |
| *Lynx canadensis* | mLynCan4_v1.p | Chromosome | 19,417 | 6,824 | 44,864 | 43.70 |

^1^All species-specific gene annotation metrics derived from the NCBI database (https://www.ncbi.nlm.nih.gov/genome/annotation_euk/all/).
