# Supplementary material for: A new domestic cat genome assembly based on long sequence reads empowers feline genomic medicine and identifies a novel gene for dwarfism
Source: PLoS Genet. 2020 Oct 22;16(10):e1008926. doi: 10.1371/journal.pgen.1008926 (PMC7581003; doi:10.1371/journal.pgen.1008926)
Supplement: S2 Table — (DOCX) [file pgen.1008926.s002.docx]

**Supplemental Table S2.** Repeat masking of 10 largest repeat classes in Felis_catus_9.0

|  | **Felis_catus_9.0** | | | | **Felis_catus_8.0** | | | |
| --- | --- | --- | --- | --- | --- | --- | --- | --- |
| Repeat class | Elements | Fragments | Genome coverage (Mb) | Genome coverage (%) | Elements | Fragments | Genome coverage (Mb) | Genome coverage (%) |
| LINE/L1 | 522993 | 893643 | 417.34 | 16.85 | 656995 | 1039054 | 465.04 | 17.89 |
| LINE/L2 | 343890 | 443837 | 93.83 | 3.79 | 360083 | 460786 | 96.60 | 3.72 |
| SINE/tRNA | 1085856 | 1165371 | 208.70 | 8.43 | 1166314 | 1242670 | 219.59 | 8.45 |
| SINE/MIR | 496362 | 521524 | 73.45 | 2.97 | 516284 | 541714 | 75.98 | 2.92 |
| LTR/ERV1 | 51793 | 74303 | 25.46 | 1.03 | 58644 | 81468 | 26.99 | 1.04 |
| LTR/ERVL | 91920 | 125049 | 42.22 | 1.71 | 99361 | 133227 | 44.11 | 1.70 |
| LTR/ERVL-MaLR | 154607 | 196428 | 54.06 | 2.18 | 165881 | 208590 | 56.57 | 2.18 |
| DNA/hAT-Charlie | 204111 | 225952 | 39.02 | 1.58 | 216443 | 238595 | 40.93 | 1.57 |
| DNA/TcMar-Tigger | 54753 | 63224 | 15.78 | 0.64 | 58397 | 67051 | 16.54 | 0.64 |
| Simple_repeat | 906674 | 911134 | 48.71 | 1.97 | 918939 | 923362 | 42.13 | 1.62 |
