# Supplementary material for: A new domestic cat genome assembly based on long sequence reads empowers feline genomic medicine and identifies a novel gene for dwarfism
Source: PLoS Genet. 2020 Oct 22;16(10):e1008926. doi: 10.1371/journal.pgen.1008926 (PMC7581003; doi:10.1371/journal.pgen.1008926)
Supplement: S3 Table — (DOCX) [file pgen.1008926.s003.docx]

**Supplemental Table S3:** Variant calling summary statistics

|  | **All variants** | **SNVs^a^** | **Indels** |
| --- | --- | --- | --- |
| Total | 46,600,527 | 39,043,080 (2.09305) | 13,304,140 |
| Biallelic | 43,213,841 | 36,606,107 (2.44973) | 6,607,734 |
| Multiallelic | 3,386,686 | 2,436,973 | 949,713 |

^a^ Numbers in braces represent ts/tv ratio
