# Supplementary material for: A new domestic cat genome assembly based on long sequence reads empowers feline genomic medicine and identifies a novel gene for dwarfism
Source: PLoS Genet. 2020 Oct 22;16(10):e1008926. doi: 10.1371/journal.pgen.1008926 (PMC7581003; doi:10.1371/journal.pgen.1008926)
Supplement: S4 Table — (DOCX) [file pgen.1008926.s004.docx]

**Supplemental Table S4**. Truth sensitivity of SNV call set.

| **Probes** | **Successfully remapped probes** | **Array SNVs found in call set** |
| --- | --- | --- |
| 62,897 | 61,258 (97.39%)^a^ | 59,246 (96.72 %)^b^ |

^a^ Percentage of all probes

^b^ Percentage of successfully remapped probes
