# Supplementary material for: A new domestic cat genome assembly based on long sequence reads empowers feline genomic medicine and identifies a novel gene for dwarfism
Source: PLoS Genet. 2020 Oct 22;16(10):e1008926. doi: 10.1371/journal.pgen.1008926 (PMC7581003; doi:10.1371/journal.pgen.1008926)
Supplement: S5 Table — (DOCX) [file pgen.1008926.s005.docx]

**Supplemental Table S5.** SNV classification by minor allele frequency in domestic cats.

| **Consequence** | **Impact** | **MAF < 1%** | **1% < MAF <10%*** | **MAF >10%** | **Total** |
| --- | --- | --- | --- | --- | --- |
| Stop gained | LoF | 212 | 334 | 292 | 838 |
| Start lost | LoF | 40 | 76 | 119 | 235 |
| Stop lost | LoF | 9 | 32 | 63 | 104 |
| Missense variant | Missense | 16476 | 29250 | 31936 | 77662 |
| Synonymous variant | Synonymous | 21812 | 47200 | 59734 | 128746 |
| Stop retained variant | Synonymous | 12 | 38 | 49 | 99 |
| Combined |  | 38562 | 76928 | 92195 | 207685 |

* MAF < 10% refers to an allele count equal to or less than 10 alleles.
