# Supplementary material for: A new domestic cat genome assembly based on long sequence reads empowers feline genomic medicine and identifies a novel gene for dwarfism
Source: PLoS Genet. 2020 Oct 22;16(10):e1008926. doi: 10.1371/journal.pgen.1008926 (PMC7581003; doi:10.1371/journal.pgen.1008926)
Supplement: S6 Table — (DOCX) [file pgen.1008926.s006.docx]

**Supplemental** **Table S6**. Values used for pairwise fisher tests of association with gene constraint.

| **SNV gene constraint association tables** | | | | | | |
| --- | --- | --- | --- | --- | --- | --- |
|  | **Synonymous** | | **Missense** | | **LoF** | |
| Constraint level | CDS SNVs | CDS non-SNVs | CDS SNVs | CDS non-SNVs | CDS SNVs | CDS non-SNVs |
| Strong | 22371 | 7218246 | 6439 | 7234178 | 39 | 7240578 |
| Medium | 13319 | 3708632 | 5290 | 3716661 | 68 | 3721883 |
| Low | 66149 | 15731156 | 41510 | 15755795 | 376 | 15796929 |
| **Singleton gene constraint association tables** | | | | | | |
|  | **Synonymous** | | **Missense** | | **LoF** | |
| Constraint level | Singleton SNVS | Non-singleton SNVS | Singleton SNVS | Non-singleton SNVS | Singleton SNVS | Non-singleton SNVS |
| Strong | 4036 | 18335 | 1766 | 4673 | 14 | 25 |
| Medium | 2378 | 10941 | 1342 | 3948 | 19 | 49 |
| Low | 11256 | 54893 | 9148 | 32362 | 96 | 280 |
