# Supplementary material for: A new domestic cat genome assembly based on long sequence reads empowers feline genomic medicine and identifies a novel gene for dwarfism
Source: PLoS Genet. 2020 Oct 22;16(10):e1008926. doi: 10.1371/journal.pgen.1008926 (PMC7581003; doi:10.1371/journal.pgen.1008926)
Supplement: S7 Table — (DOCX) [file pgen.1008926.s007.docx]

**Supplemental** **Table S7**. Feline LoF singletons in human genes under strong constraint.

| **SNV location** | **Consequence** | **SYMBOL** | **pLI** | **Individual ID** | **Supported by NCBI CDS annotation** | **Disease** | **Status** |
| --- | --- | --- | --- | --- | --- | --- | --- |
| chrA1:108024733 | stop_gained | *FBN2* | 0.99999 | felCat.Fcat19725.Fenrisulfr | No | Hypotrichia | affected |
| chrA1:116653102 | stop_gained | *FAM13B* | 0.92003 | felCat.Fcat19194.Pudge | Yes | Ectodermal dysplasia | affected |
| chrA1:192824838 | stop_gained | *CYFIP2* | 1 | felCat.Fcat20406.Gannon | Yes | stones | affected |
| chrA1:195100556 | stop_gained | *LARP1* | 1 | felCat.SPF19984.SPFHarlem | No |  |  |
| chrA1:216234193 | stop_gained | *PDZD2* | 0.99156 | felCat.Fcat18579.Madagascar | No |  |  |
| chrA1:216236592 | stop_gained | *PDZD2* | 0.99156 | felCat.Fcat11849.Iraq | No |  |  |
| chrB1:77305060 | stop_gained | *FBXW7* | 0.99984 | felCat.Fcat5012.Colorado | Yes | Lymphoma | carrier |
| chrB4:41749665 | stop_gained | *ZNF384* | 0.99872 | felCat.S792.Haku | No |  |  |
| chrB4:86010265 | stop_gained | *KIF5A* | 0.9999 | felCat.SPF19984.SPFHarlem | Yes |  |  |
| chrC1:14006692 | stop_gained | *UBR4* | 1 | felCat.Fcat17994.Camila | No | Hydrocephalus | carrier |
| chrC1:14006828 | start_lost | *UBR4* | 1 | felCat.Fcat18849.Iowa | No |  |  |
| chrC2:69450306 | stop_gained | *MYLK* | 0.96358 | felCat.Fcat20425.Rocket | No | Hypokalemia | affected |
| chrD1:108097571 | stop_gained | *INCENP* | 0.98884 | felCat.Fcat18801.Italy | No |  |  |
| chrD2:63980875 | stop_gained | *SH3PXD2A* | 0.99821 | felCat.CR1397.Isabella | Yes | Infectious peritonitis | affected |
| chrD4:16547403 | stop_gained | *RORB* | 0.99959 | felCat.Fcat18528.Denmark | Yes |  |  |
| chrD4:75416950 | stop_gained | *PRPF4* | 0.99926 | felCat.Fcat18528.Denmark | Yes |  |  |
