# Supplementary material for: A new domestic cat genome assembly based on long sequence reads empowers feline genomic medicine and identifies a novel gene for dwarfism
Source: PLoS Genet. 2020 Oct 22;16(10):e1008926. doi: 10.1371/journal.pgen.1008926 (PMC7581003; doi:10.1371/journal.pgen.1008926)
Supplement: S8 Table — (DOCX) [file pgen.1008926.s008.docx]

**Supplemental Table S8.** GATK variant filtering criteria.

| **Filter name** | **SNV GATK** | **SNV Strict** | **Indel GATK** | **Indel Strict** |
| --- | --- | --- | --- | --- |
| QD | X < 2.0 | X < 8.0 | X < 2.0 | X < 8.0 |
| FS | X > 60.0 | X > 20.0 | X > 200.0 | X > 20.0 |
| SOR | X > 3.0 | X > 2.0 | X > 10.0 | X > 2.0 |
| ReadPosRankSum | X < -8.0 | X < -2.0 \|\| X > 2.0 | X < -20.0 | X < -2.0 \|\| X > 2.0 |
| MQ | X < 40.0 | X < 55.0 | NA | X < 55.0 |
| MQRankSum | X < -12.5 | X < -0.2 \|\| X > 0.2 | NA | X < -0.2 \|\| X > 0.2 |

Filters are as follows: variant quality by read depth (QD), fisher test for strand (FS), strand odds ratio (SOR), read position rank sum test (ReadPosRankSum), mapping quality (MQ), and mapping quality rank sum (MQRankSum).
