# Supplementary material for: A new domestic cat genome assembly based on long sequence reads empowers feline genomic medicine and identifies a novel gene for dwarfism
Source: PLoS Genet. 2020 Oct 22;16(10):e1008926. doi: 10.1371/journal.pgen.1008926 (PMC7581003; doi:10.1371/journal.pgen.1008926)
Supplement: S9 Table — (DOCX) [file pgen.1008926.s009.docx]

**Supplemental** **Table S9**. PCR Primers for the genotyping of feline disproportionate dwarfism.

| **Number on Fig S1** | **Primer** | **Sequence 5’ 🡪 3’** |
| --- | --- | --- |
| 1 | UGDH_mid_R | TGGAGATGTGCACCTTCATC |
| 2 | UGDH_mid_F | GGCGTAAACACATTTTCTTGC |
| 3 | UGDH_del_R | CGGCATACAAGTCAGCCTTC |
| 4 | UGDH_down_R | GGGCAAAATTGGGGACTAAC |
| 5 | UGDH_up_F | CAGTGTGTGGCATAGGCTTC |
