# Supplementary figures and images for: A new domestic cat genome assembly based on long sequence reads empowers feline genomic medicine and identifies a novel gene for dwarfism
Source: PLoS Genet. 2020 Oct 22;16(10):e1008926. doi: 10.1371/journal.pgen.1008926 (PMC7581003; doi:10.1371/journal.pgen.1008926)

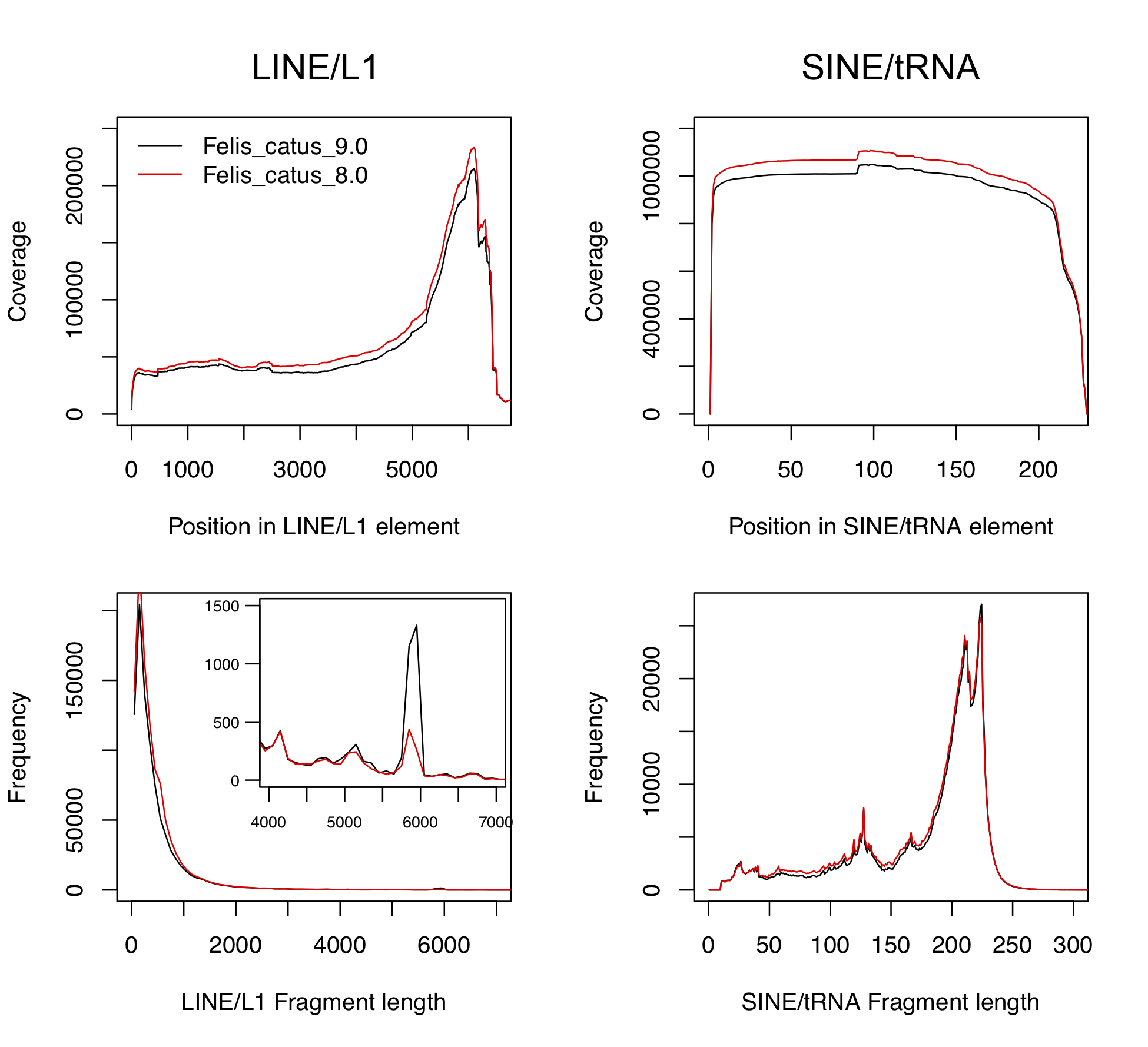

Supplement: S1 Fig — Inset focusses on full length L1 fragments, which only make up a small fraction of all L1s. (TIFF) [file pgen.1008926.s010.tiff]

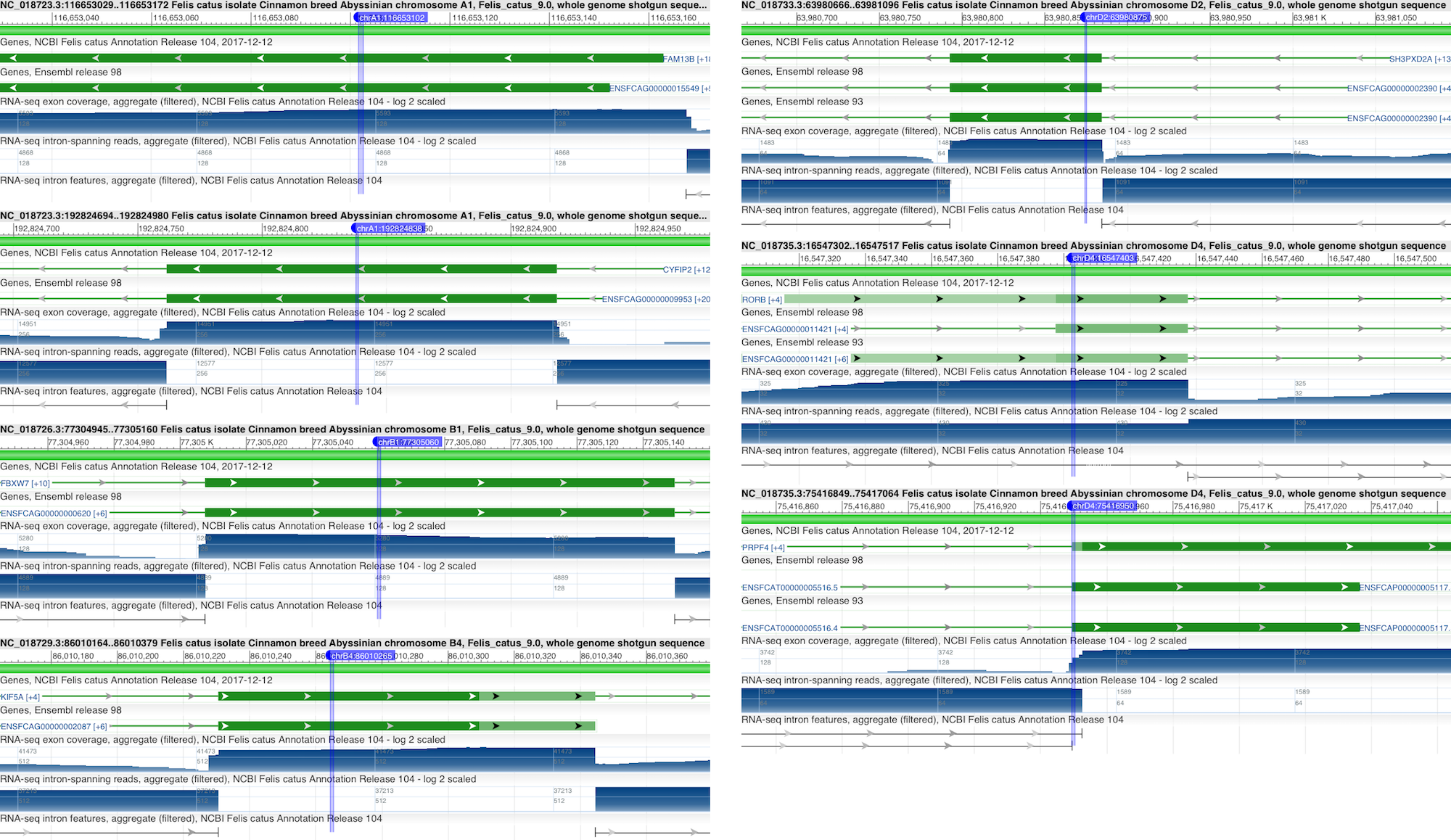

Supplement: S2 Fig — The SNV position is highlighted in each figure panel in blue. Images were generated using NCBI’s graphics option through the Nucleotide database. (TIFF) [file pgen.1008926.s011.tiff]

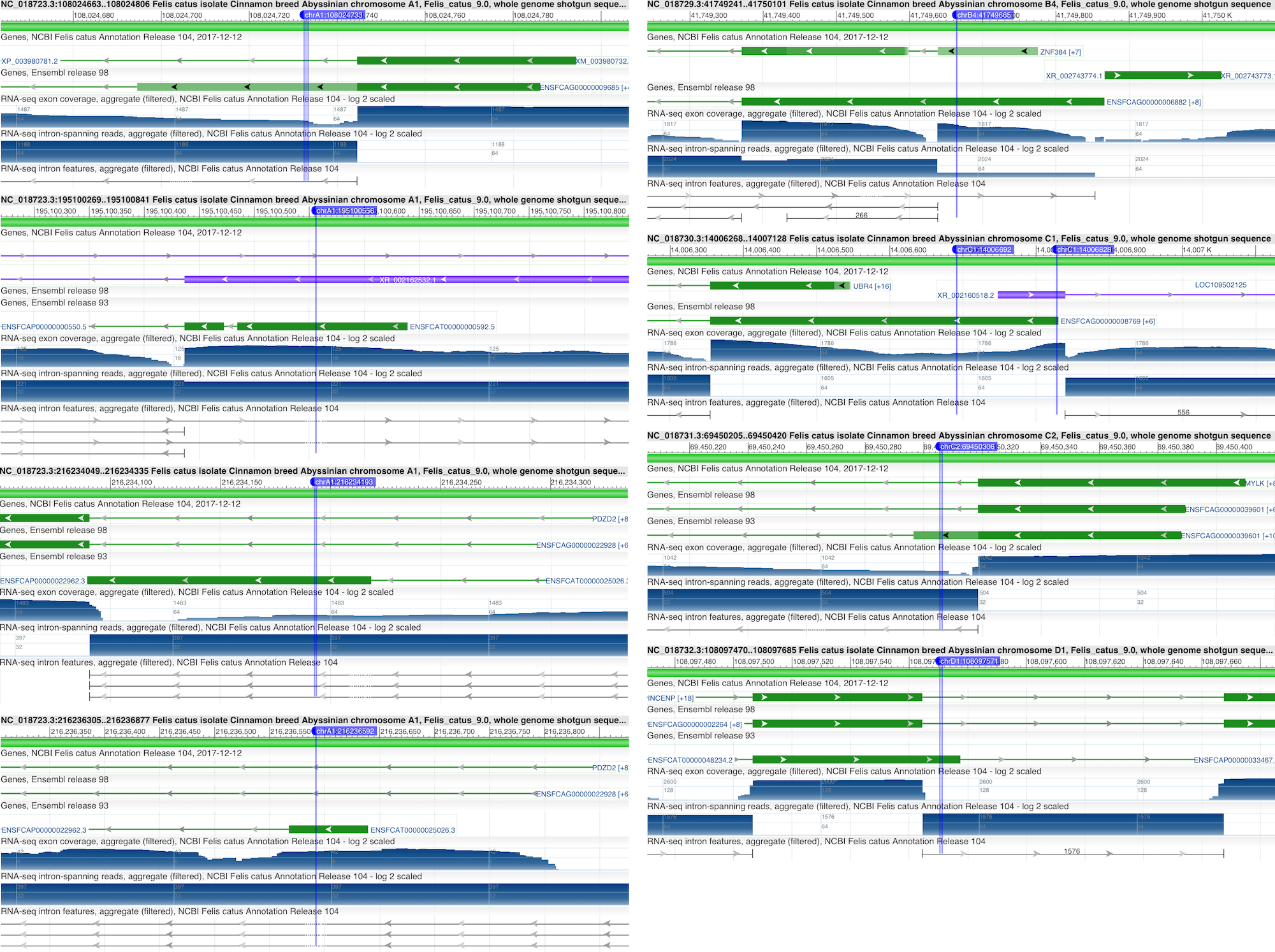

Supplement: S3 Fig — The SNV position is highlighted in each figure panel in blue. Images were generated using NCBI’s graphics option through the Nucleotide database. (TIFF) [file pgen.1008926.s012.tiff]

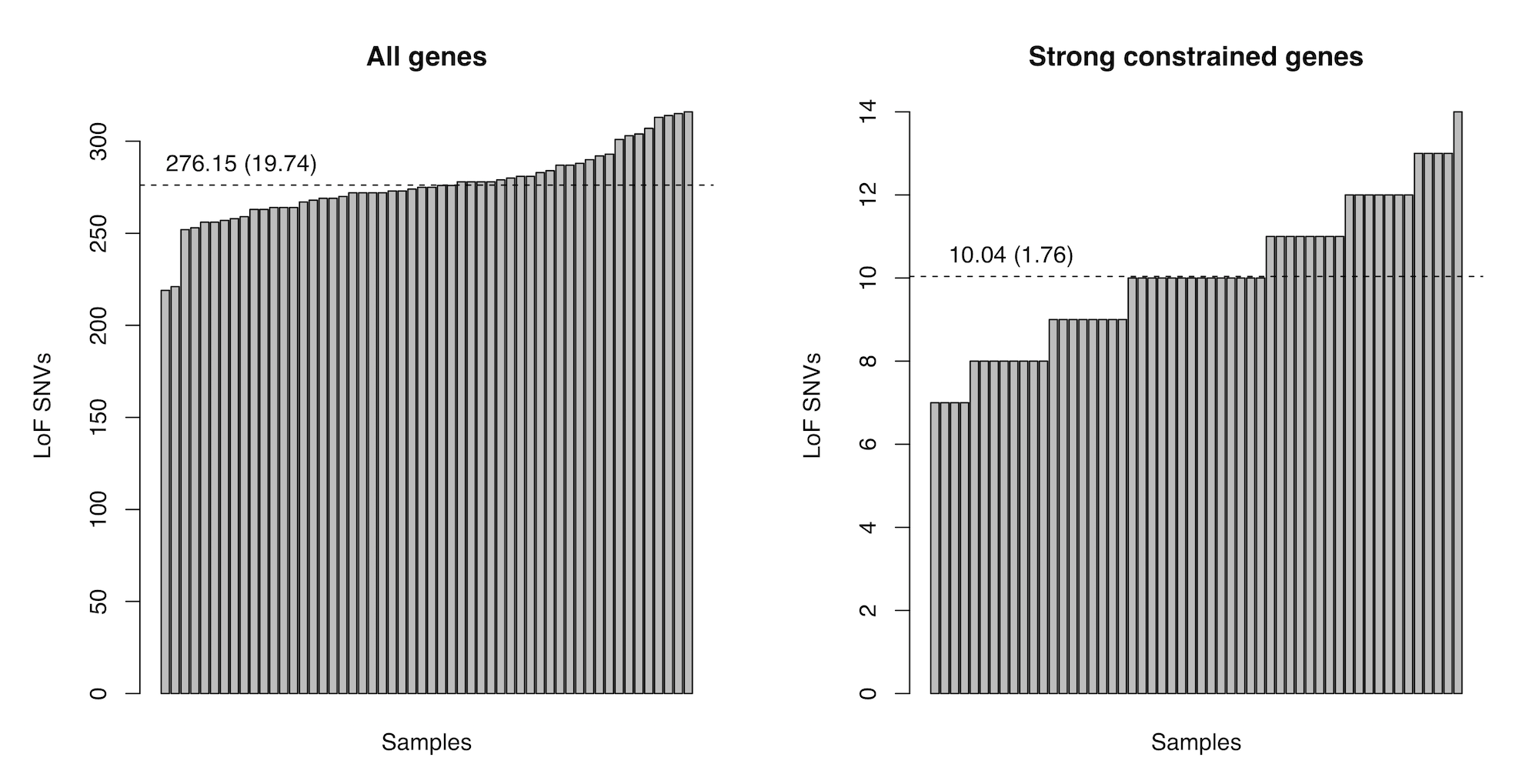

Supplement: S4 Fig — Dotted line shows the mean value, which is also stated above along with standard deviation in braces. (TIFF) [file pgen.1008926.s013.tiff]

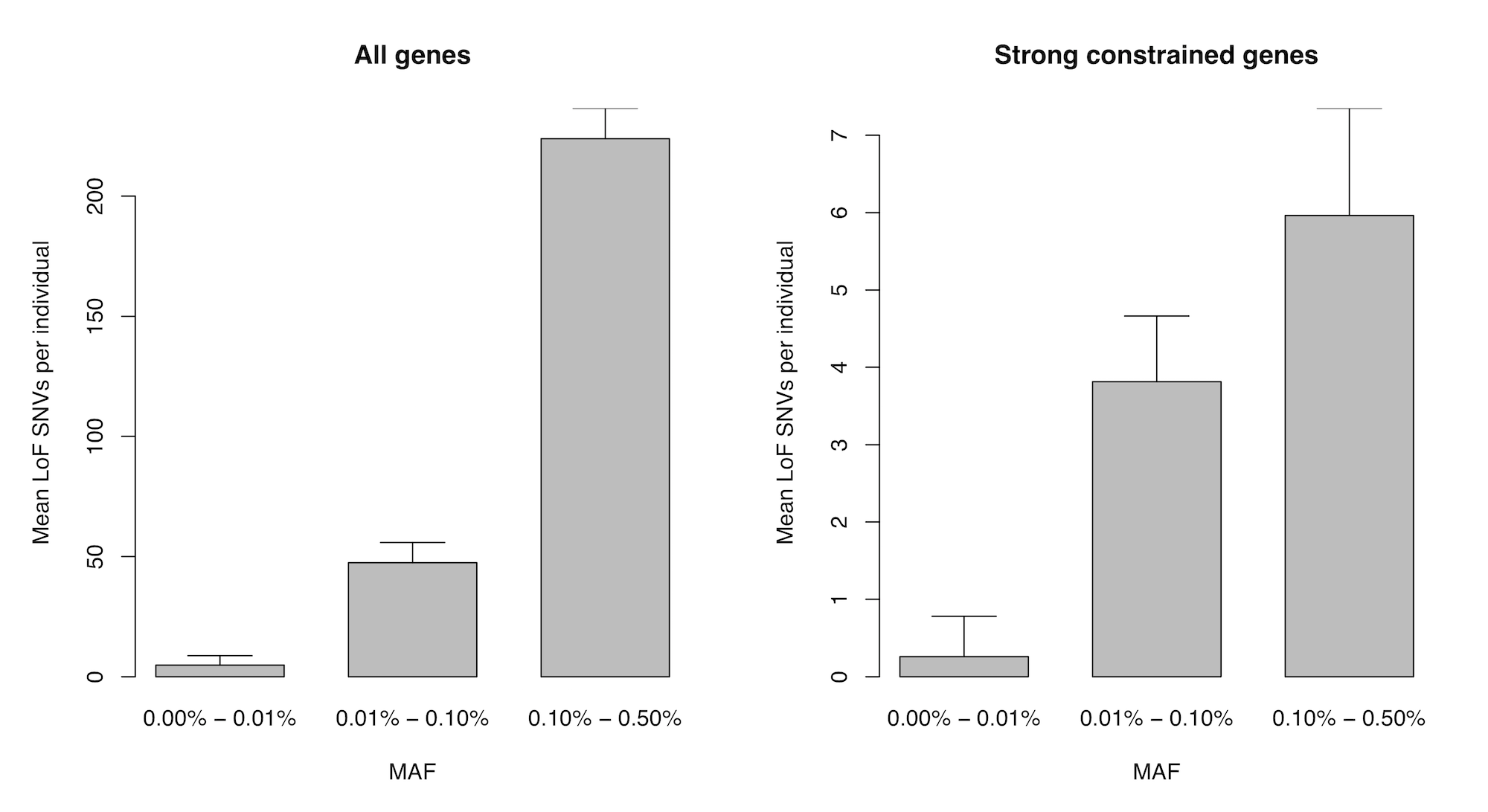

Supplement: S5 Fig — Error bars represent 1 standard deviation. (TIFF) [file pgen.1008926.s014.tiff]

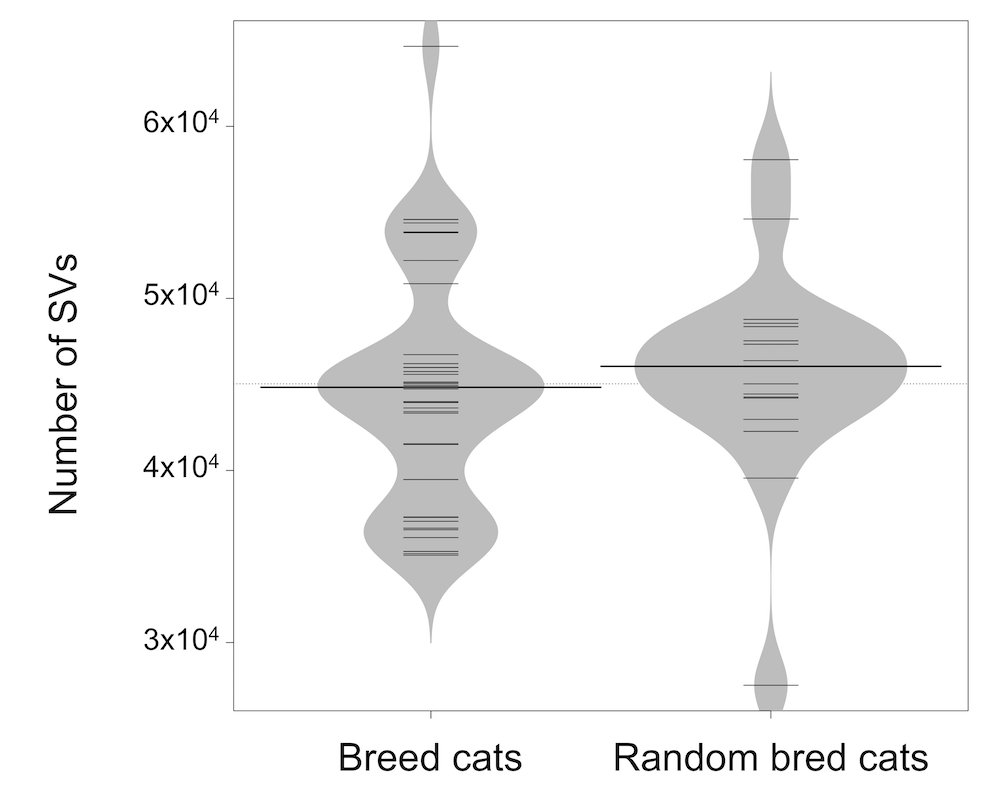

Supplement: S6 Fig — (TIFF) [file pgen.1008926.s015.tiff]

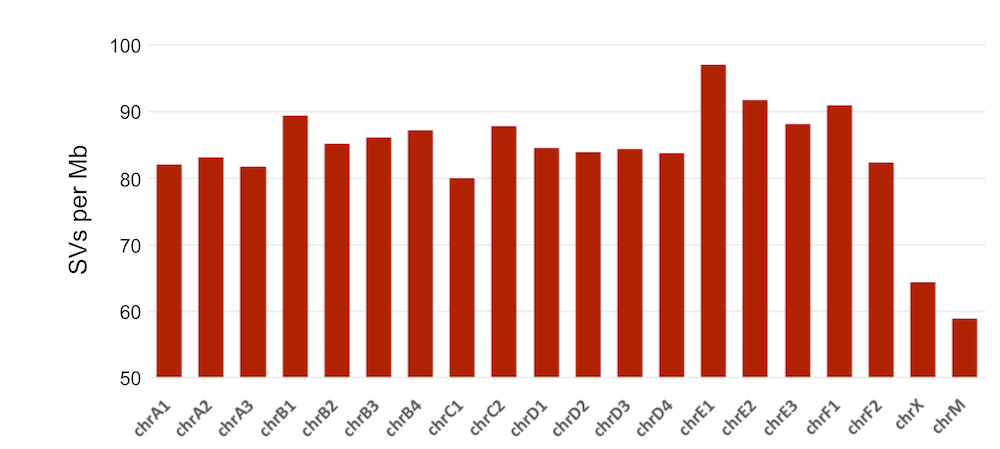

Supplement: S7 Fig — (TIFF) [file pgen.1008926.s016.tiff]

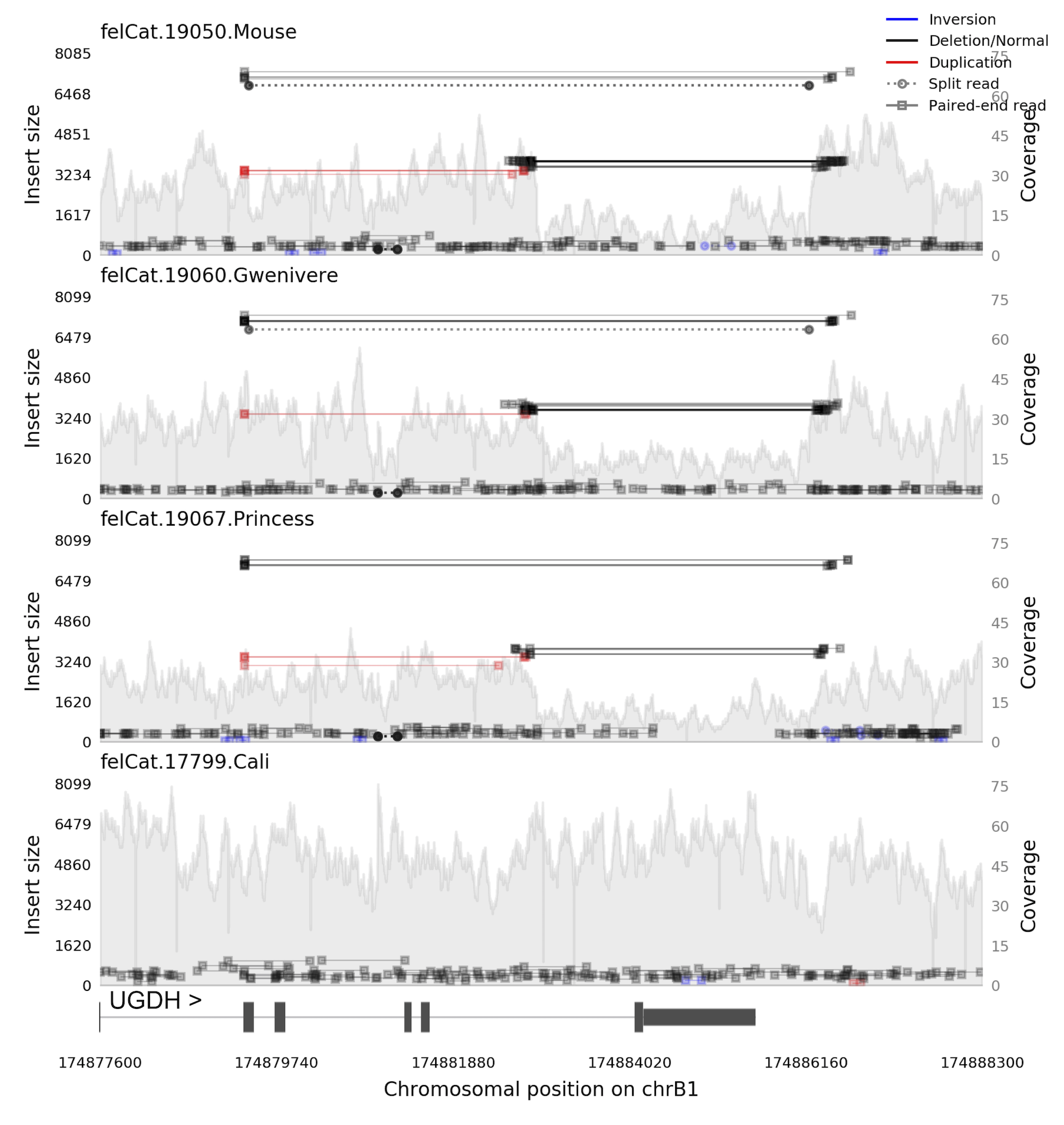

Supplement: S8 Fig — Unrelated affected cats are felCat.19050.Mouse, felCat.19060.Gwenivere, and felCat.19067.Princess. felCat.17799.Cali is an unaffected normal control cat. Coverage across the control cat is relatively uniform, while the affected cats show decreased coverage over the final exon of UGDH marking a heterozygous deletion. Discordant reads that span beyond the deletion show sequence into the deleted region shares homology with UGDH exon 8. (TIFF) [file pgen.1008926.s017.tiff]

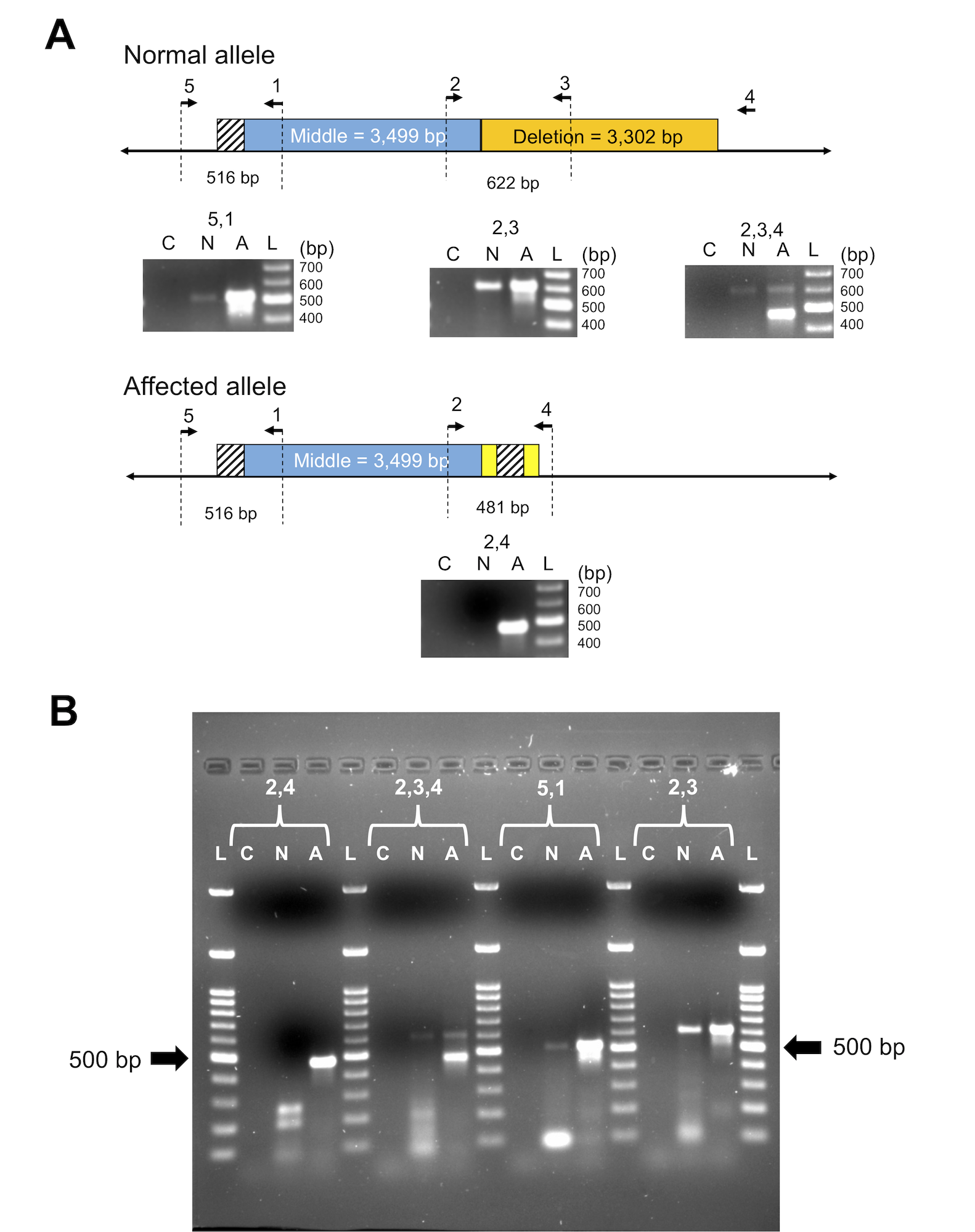

Supplement: S9 Fig — (a) Arrows represent individual primers and predicted band sizes. Gel photos show no template control (C), normal sample (N), affected sample (A), and 100 bp PLUS™ DNA Ladder (Gold Biotechnology, Inc., St. Louis, MO) (L). Ladder sizes are shown to the right of each gel image in bp. Above each gel image is the primers that were used to generate the band in each sample. Band sizes were consistent with predicted breakpoint lengths. Hatched square is 49 bp segment that shares homology with exon 8, it is consistent with a duplication and insertion into deleted region. Yellow boxes represent sequence of unknown origin found in affected allele. The deletion is absent from the affected allele, allowing primer four to produce an amplicon with primer 2. All dwarf samples analyzed were heterozygous for the affected allele. Primers 1 –UDGH_mid_R, 2 –UDGH_mid_F, 3 –UDGH_del_R, 4 –UDGH_down_R, 5 –UDGH_up_F. (b) Full gel image for primer combinations described in A. (TIFF) [file pgen.1008926.s018.tiff]

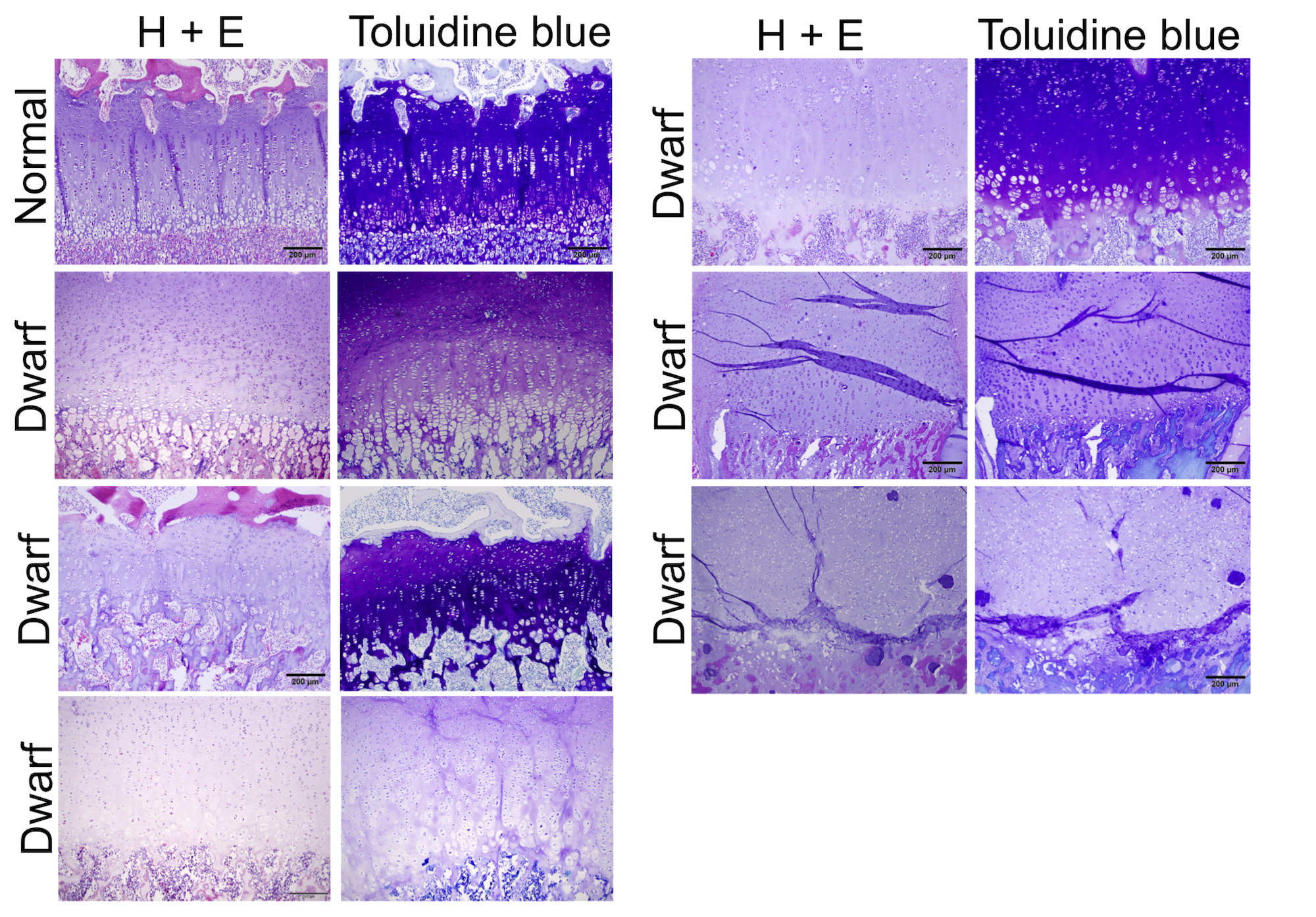

Supplement: S10 Fig — H&E and toluidine blue histologic samples of the distal radius epiphyseal cartilage plate from a normal neonatal kitten and age-matched dwarf kittens. For normal control kitten, H&E shows chondrocytes in the growth plate exhibit a regular columnar arrangement and are organized into a zone of reserve cells, a zone of proliferation and a zone of hypertrophy and a zone of provisional calcification. For the same kitten toluidine blue stain shows physeal cartilage contains abundant proteoglycan as shown by its metachromasia. In dwarf samples H&E staining consistently shows chondrocytes in the growth plate exhibit an irregular columnar arrangement. For four of the six dwarf samples, toluidine blue staining shows by its metachromasia that dwarf cat physeal cartilage contains lessor amounts of proteoglycans. (TIFF) [file pgen.1008926.s019.tiff]
