# Supplementary material for: A new domestic cat genome assembly based on long sequence reads empowers feline genomic medicine and identifies a novel gene for dwarfism
Source: PLoS Genet. 2020 Oct 22;16(10):e1008926. doi: 10.1371/journal.pgen.1008926 (PMC7581003; doi:10.1371/journal.pgen.1008926)
Supplement: S1 Data — (PDF) [file pgen.1008926.s020.pdf]

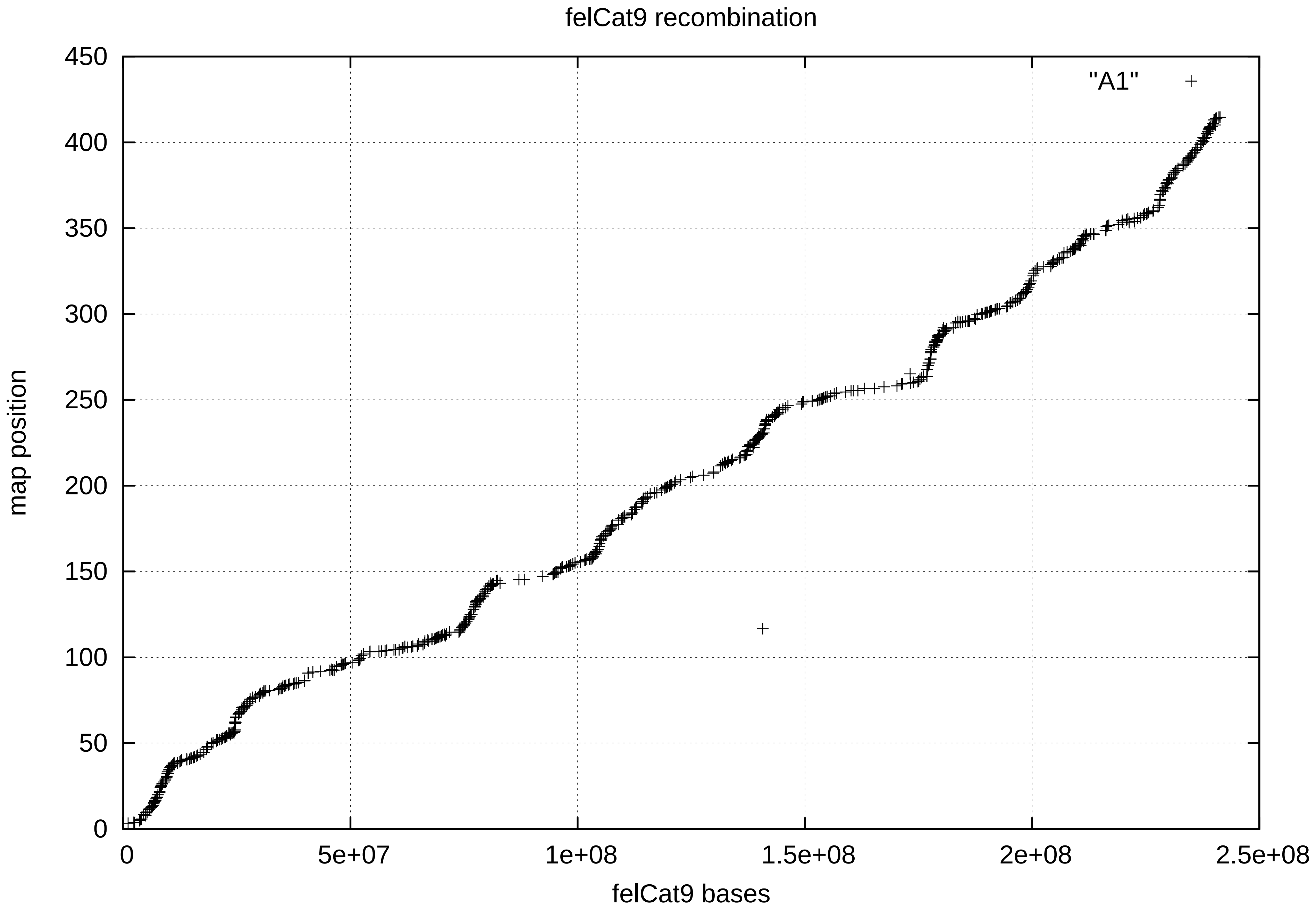
felCat9 recombination
"A1"
map position
felCat9 bases
0
50
100
150
200
250
300
350
400
450
0
5e+07
1e+08
1.5e+08
2e+08
2.5e+08

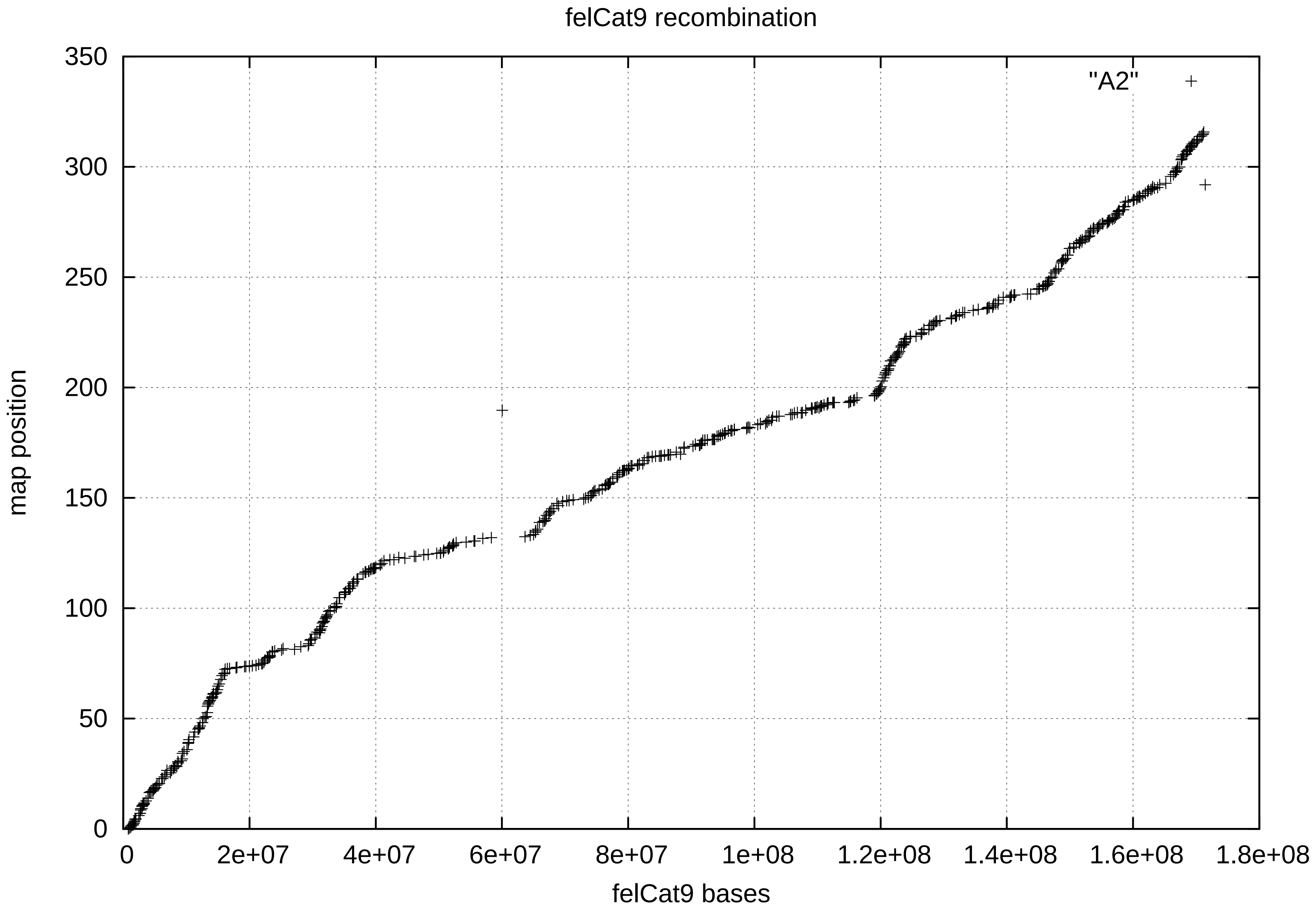

felCat9 recombination
"A2"
map position
felCat9 bases
0
50
100
150
200
250
300
350
0
2e+07
4e+07
6e+07
8e+07
1e+08
1.2e+08
1.4e+08
1.6e+08
1.8e+08

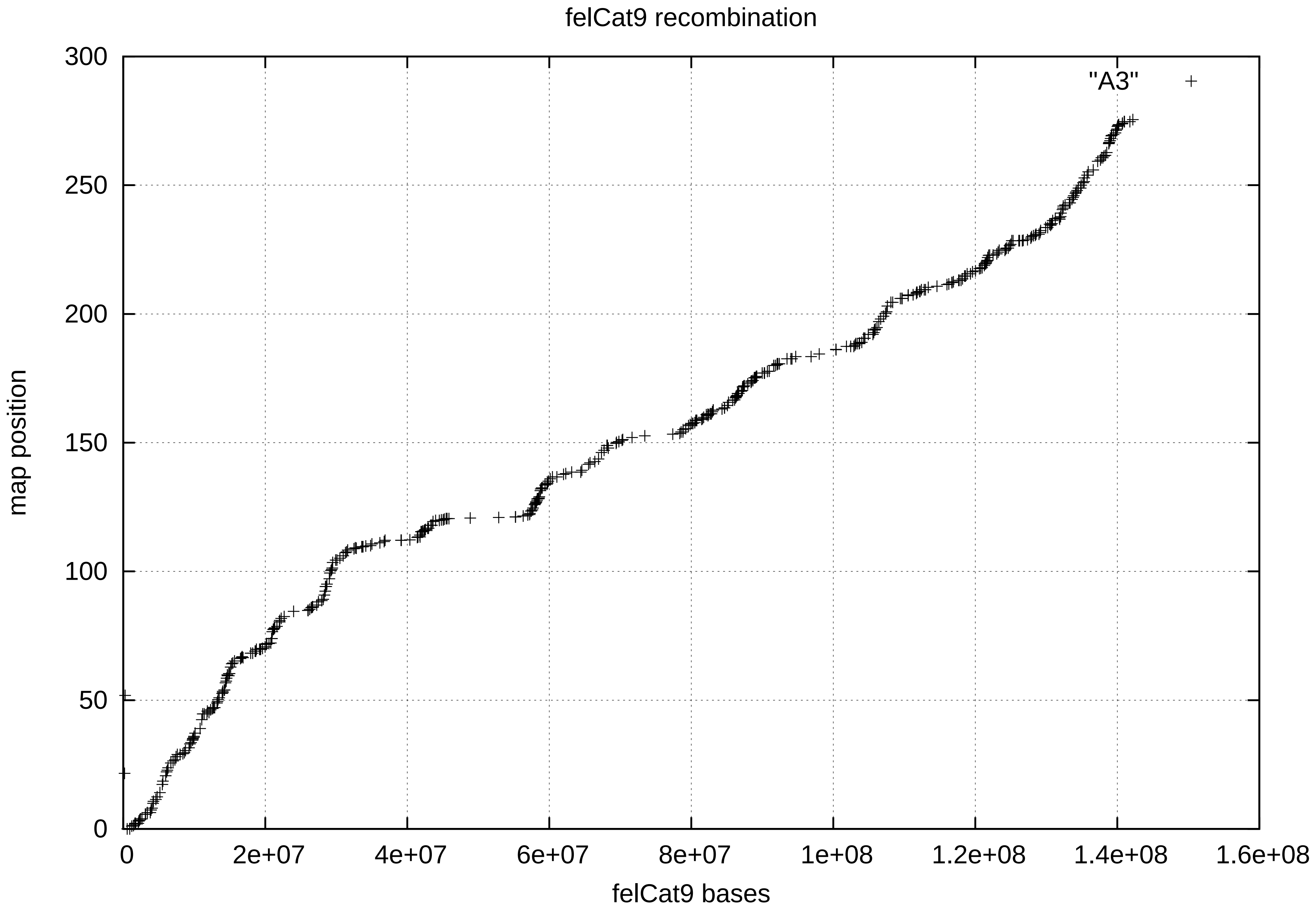

felCat9 recombination
"A3"
map position
felCat9 bases
0
50
100
150
200
250
300
0
2e+07
4e+07
6e+07
8e+07
1e+08
1.2e+08
1.4e+08
1.6e+08

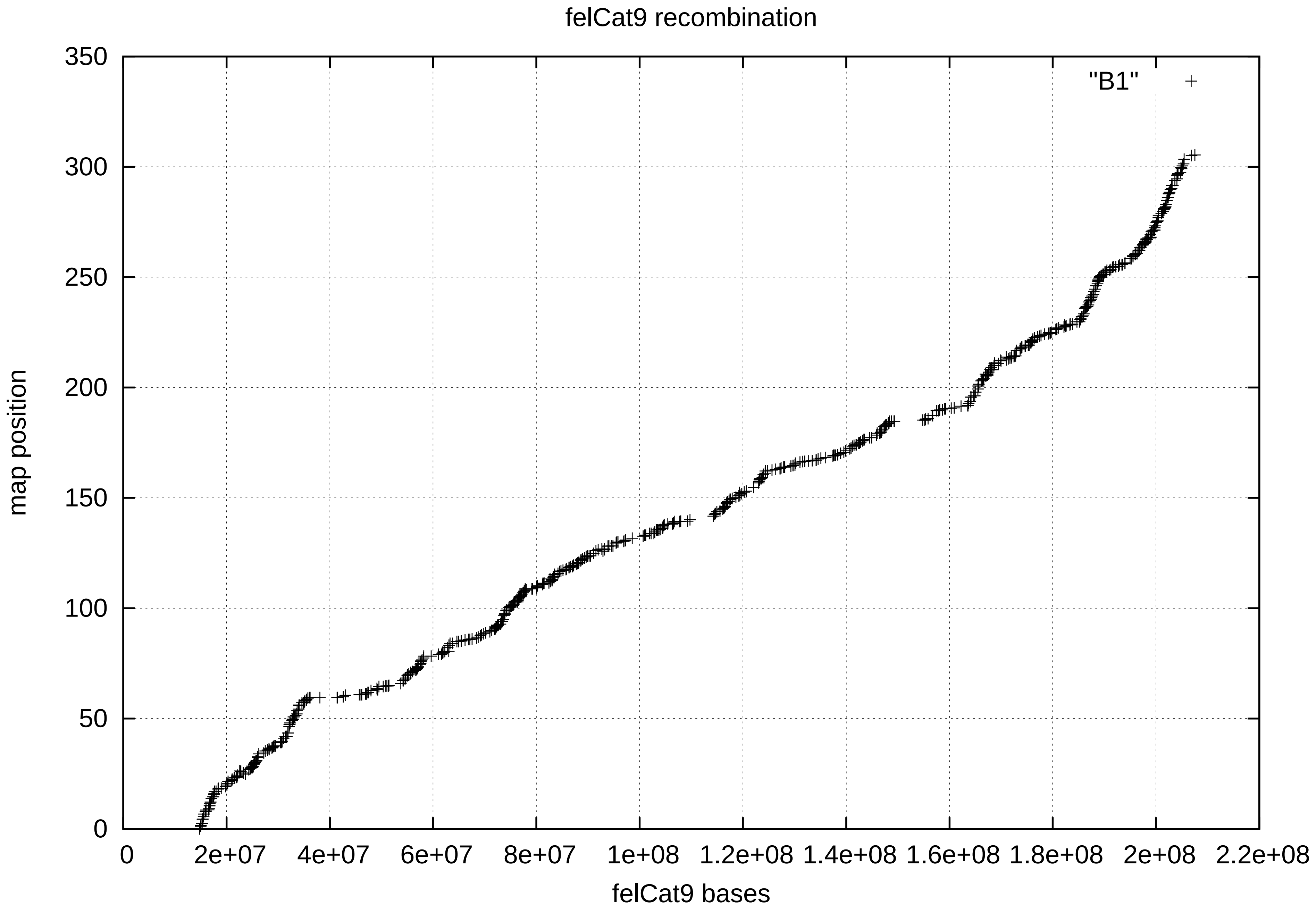
felCat9 recombination
"B1"
map position
felCat9 bases
0
50
100
150
200
250
300
350
0
2e+07
4e+07
6e+07
8e+07
1e+08
1.2e+08
1.4e+08
1.6e+08
1.8e+08
2e+08
2.2e+08

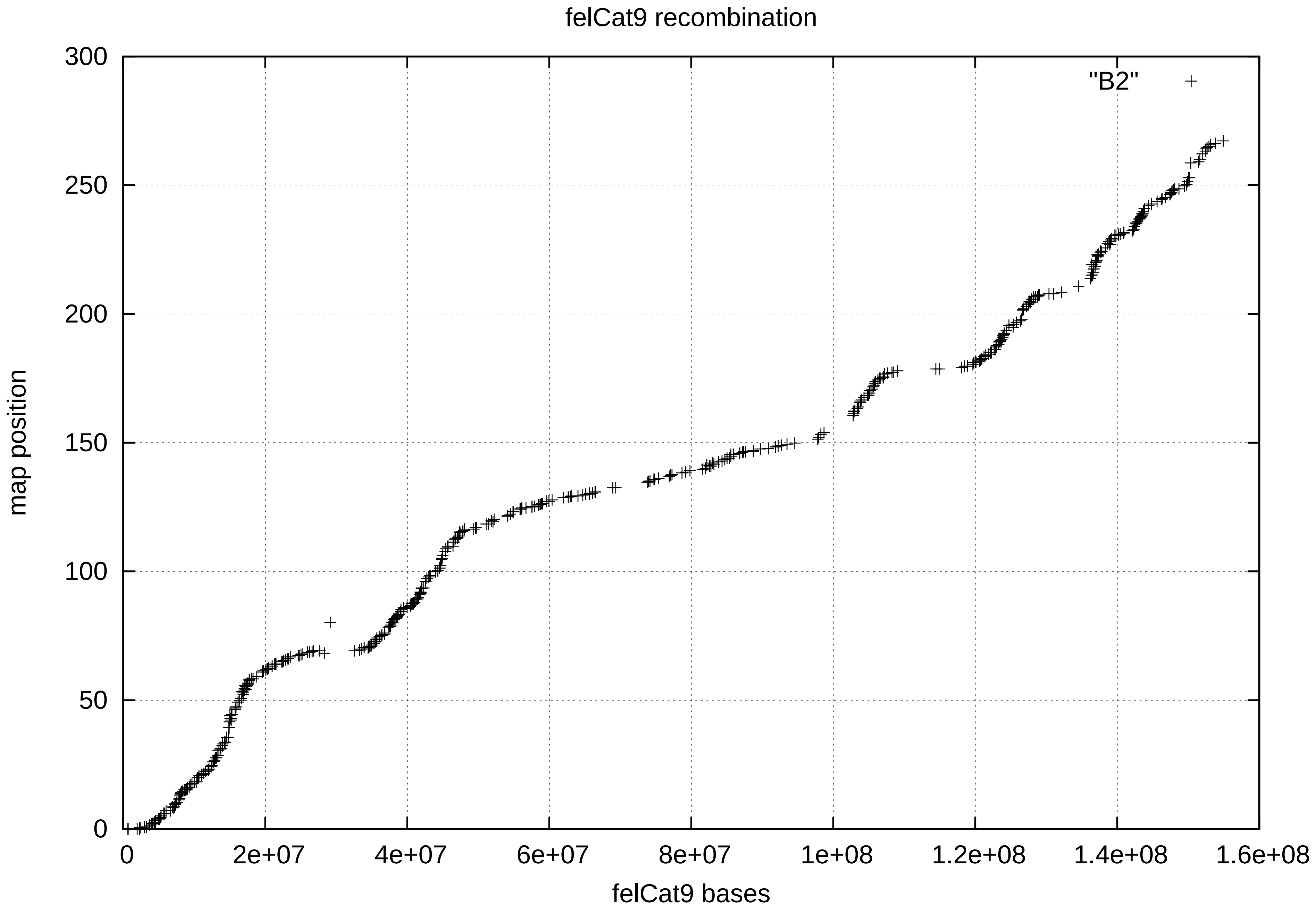

felCat9 recombination
"B2"
map position
felCat9 bases
0
50
100
150
200
250
300
0
2e+07
4e+07
6e+07
8e+07
1e+08
1.2e+08
1.4e+08
1.6e+08

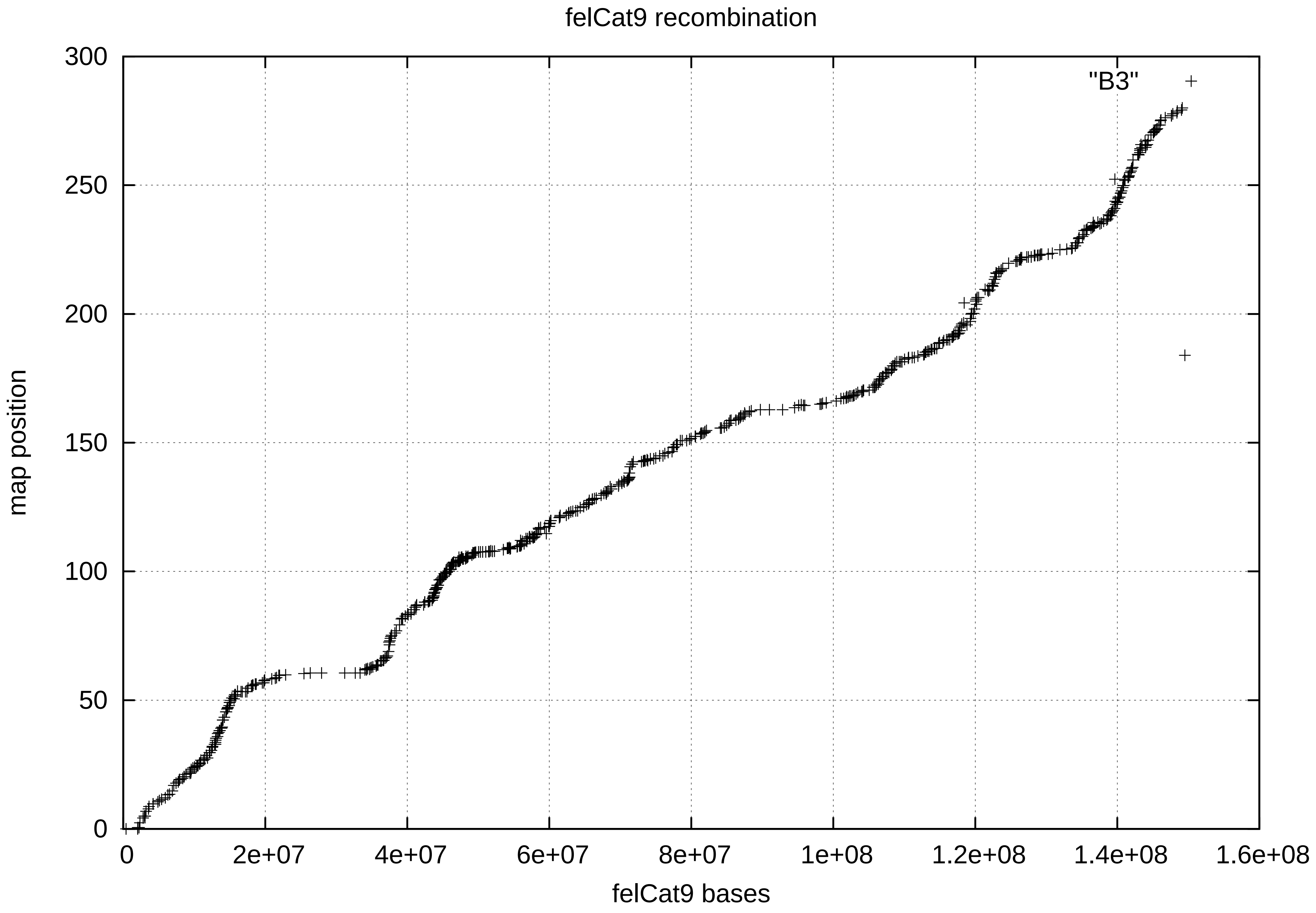
felCat9 recombination
"B3"
map position
felCat9 bases
0
50
100
150
200
250
300
0
2e+07
4e+07
6e+07
8e+07
1e+08
1.2e+08
1.4e+08
1.6e+08

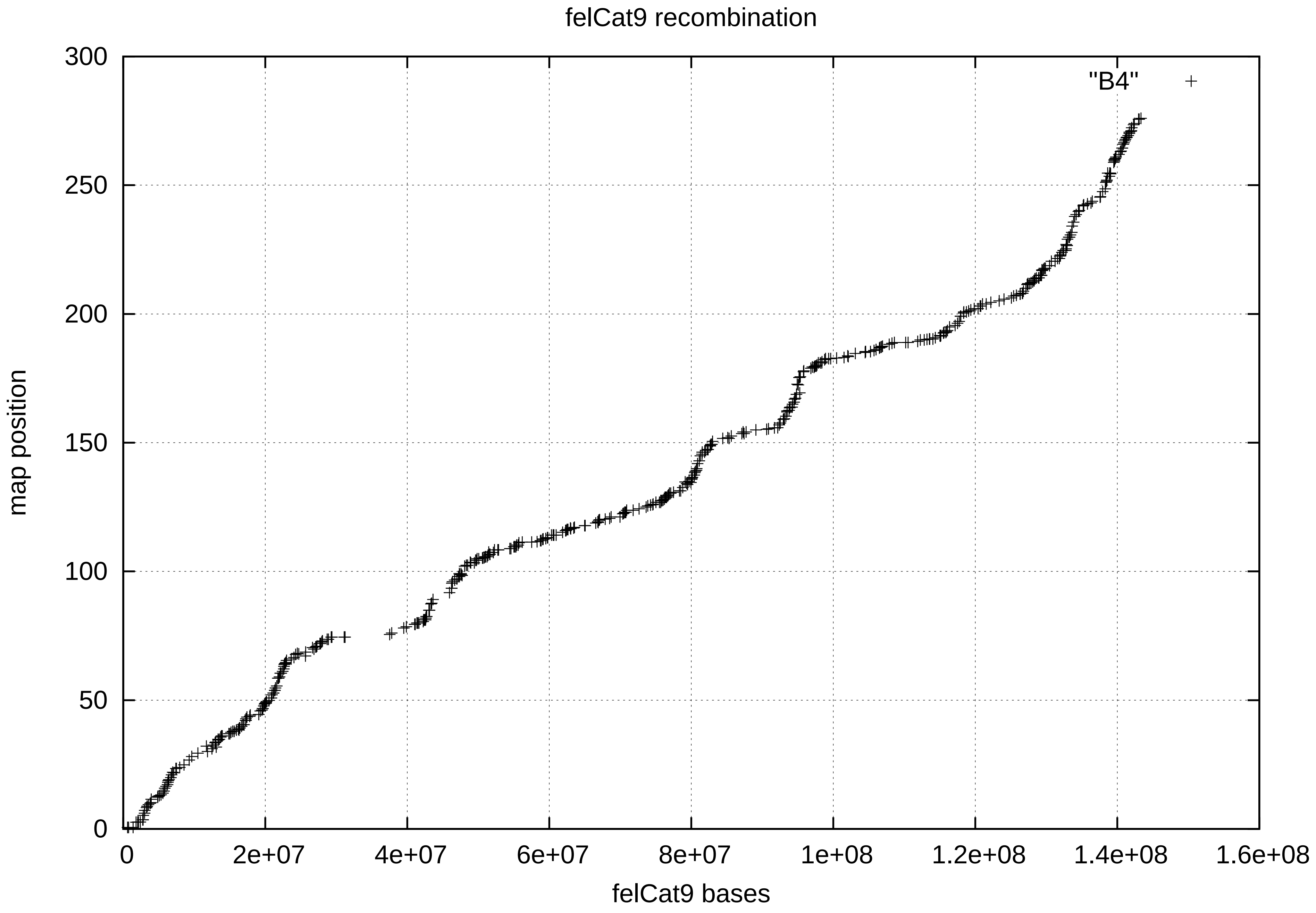
felCat9 recombination
"B4"
map position
felCat9 bases
0
50
100
150
200
250
300
0
2e+07
4e+07
6e+07
8e+07
1e+08
1.2e+08
1.4e+08
1.6e+08

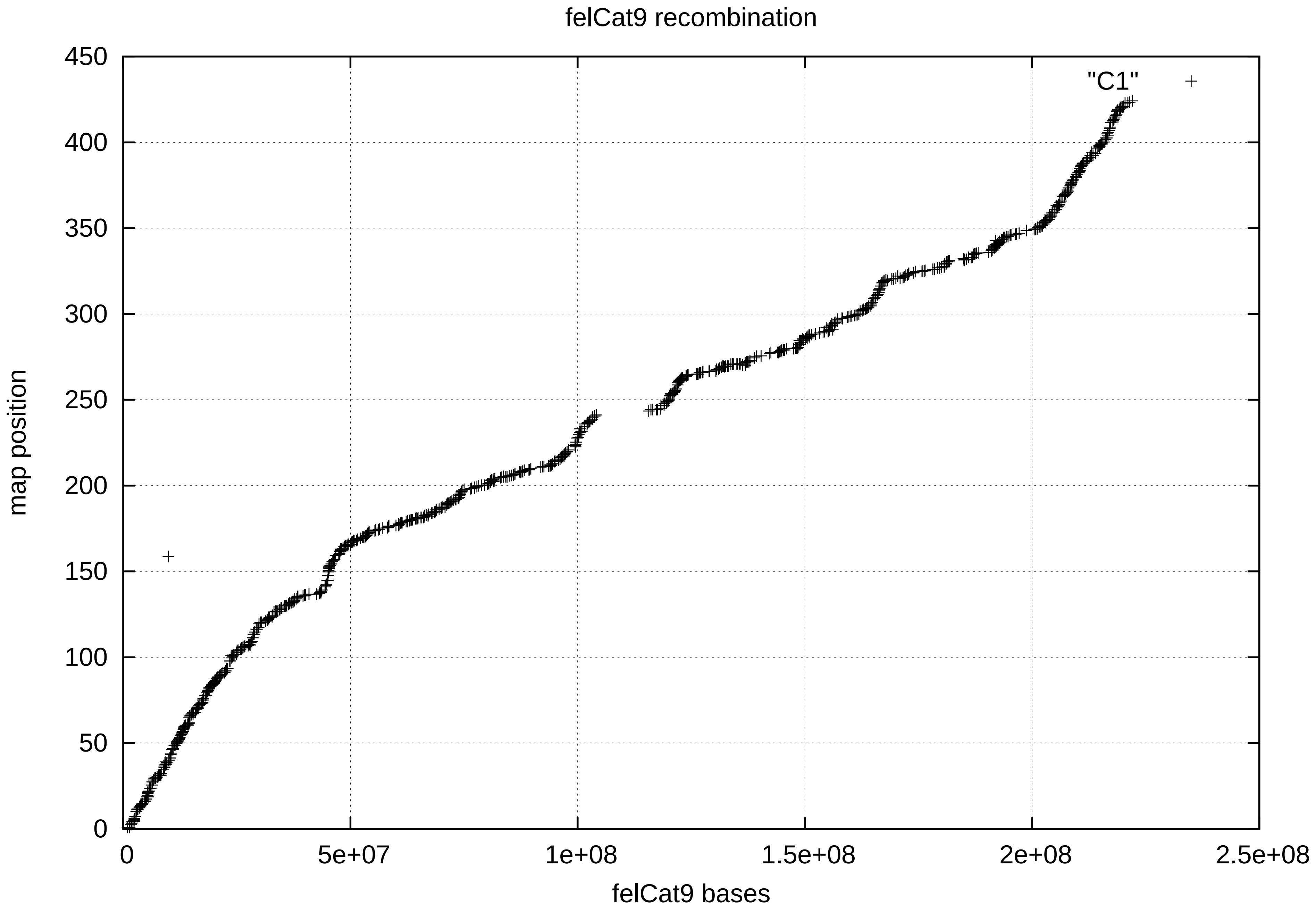
felCat9 recombination
map position
felCat9 bases
"C1"
0
50
100
150
200
250
300
350
400
450
0
5e+07
1e+08
1.5e+08
2e+08
2.5e+08

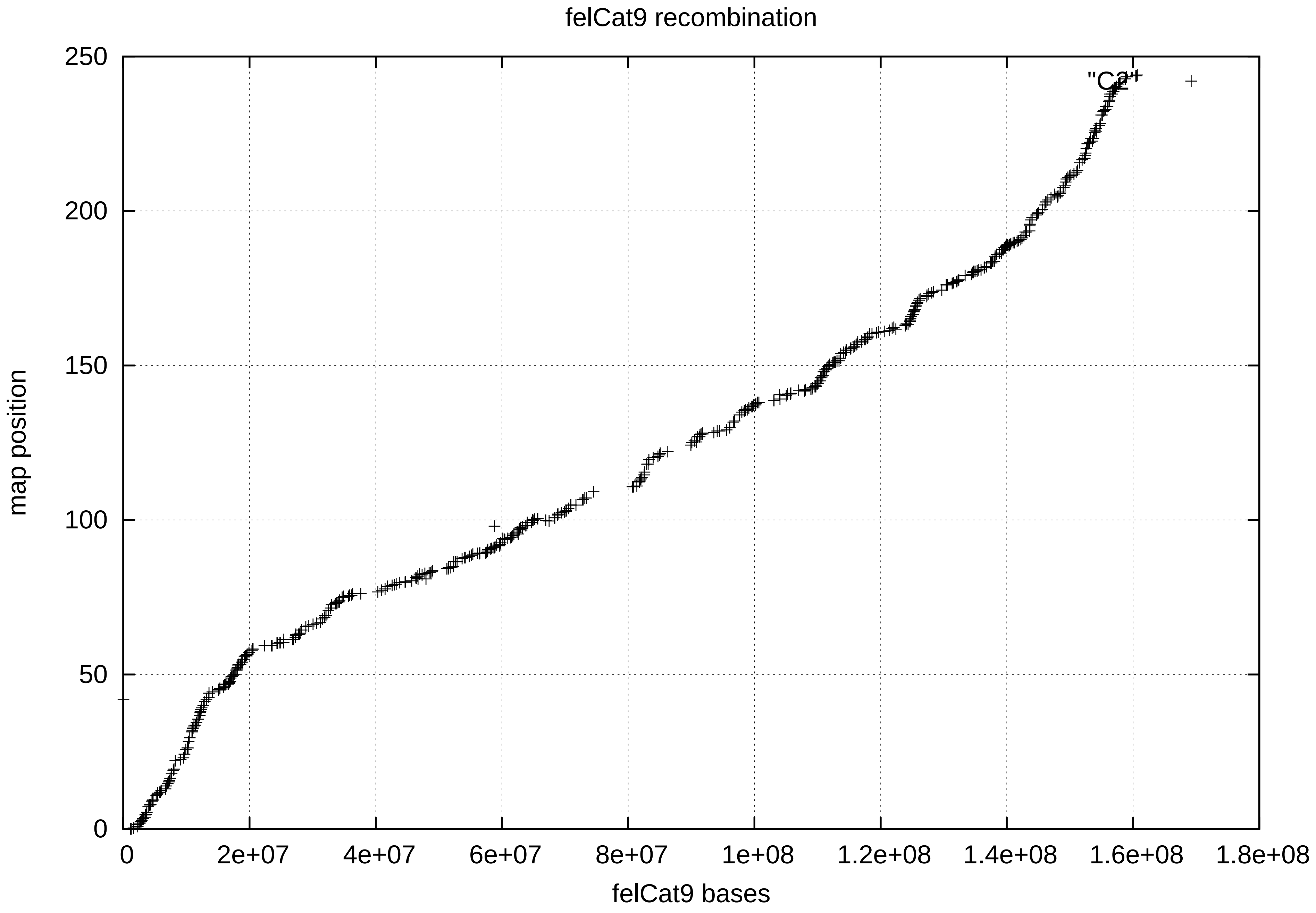

felCat9 recombination
"C2"
map position
felCat9 bases
250
200
150
100
50
0
0
2e+07
4e+07
6e+07
8e+07
1e+08
1.2e+08
1.4e+08
1.6e+08
1.8e+08

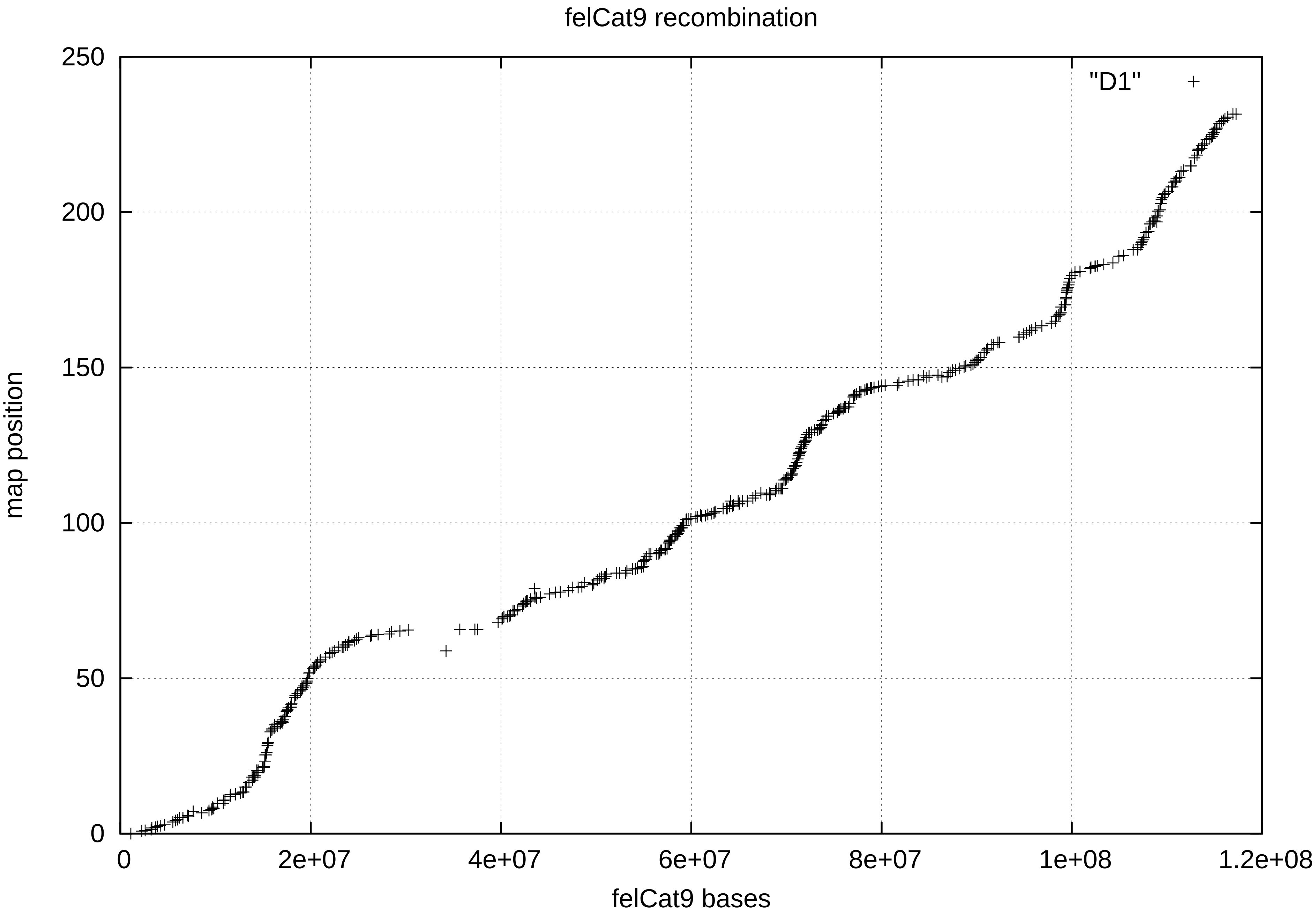

felCat9 recombination
"D1"
map position
felCat9 bases
0
50
100
150
200
250
0
2e+07
4e+07
6e+07
8e+07
1e+08
1.2e+08

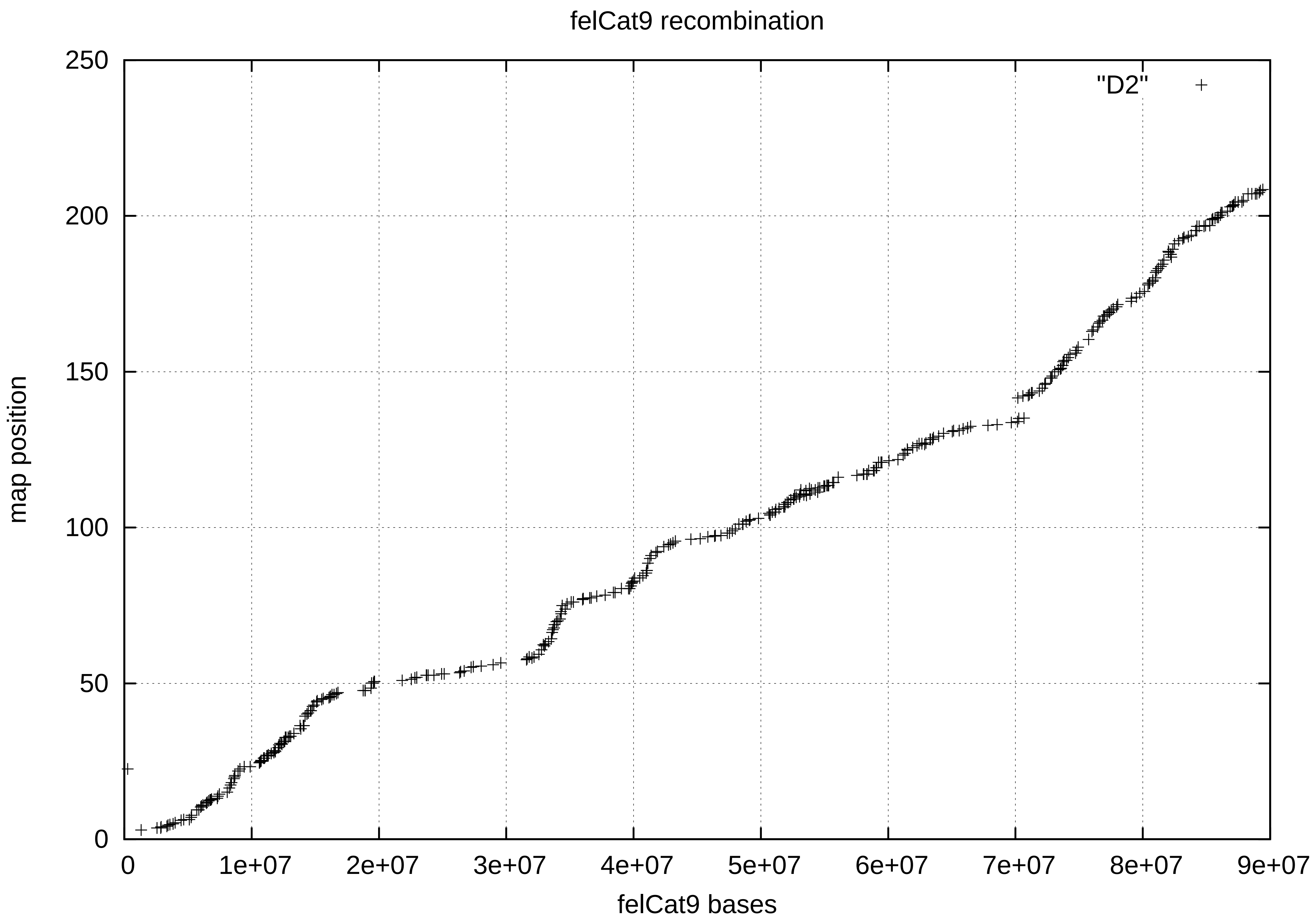
felCat9 recombination
"D2"
map position
felCat9 bases
0
50
100
150
200
250
0
1e+07
2e+07
3e+07
4e+07
5e+07
6e+07
7e+07
8e+07
9e+07

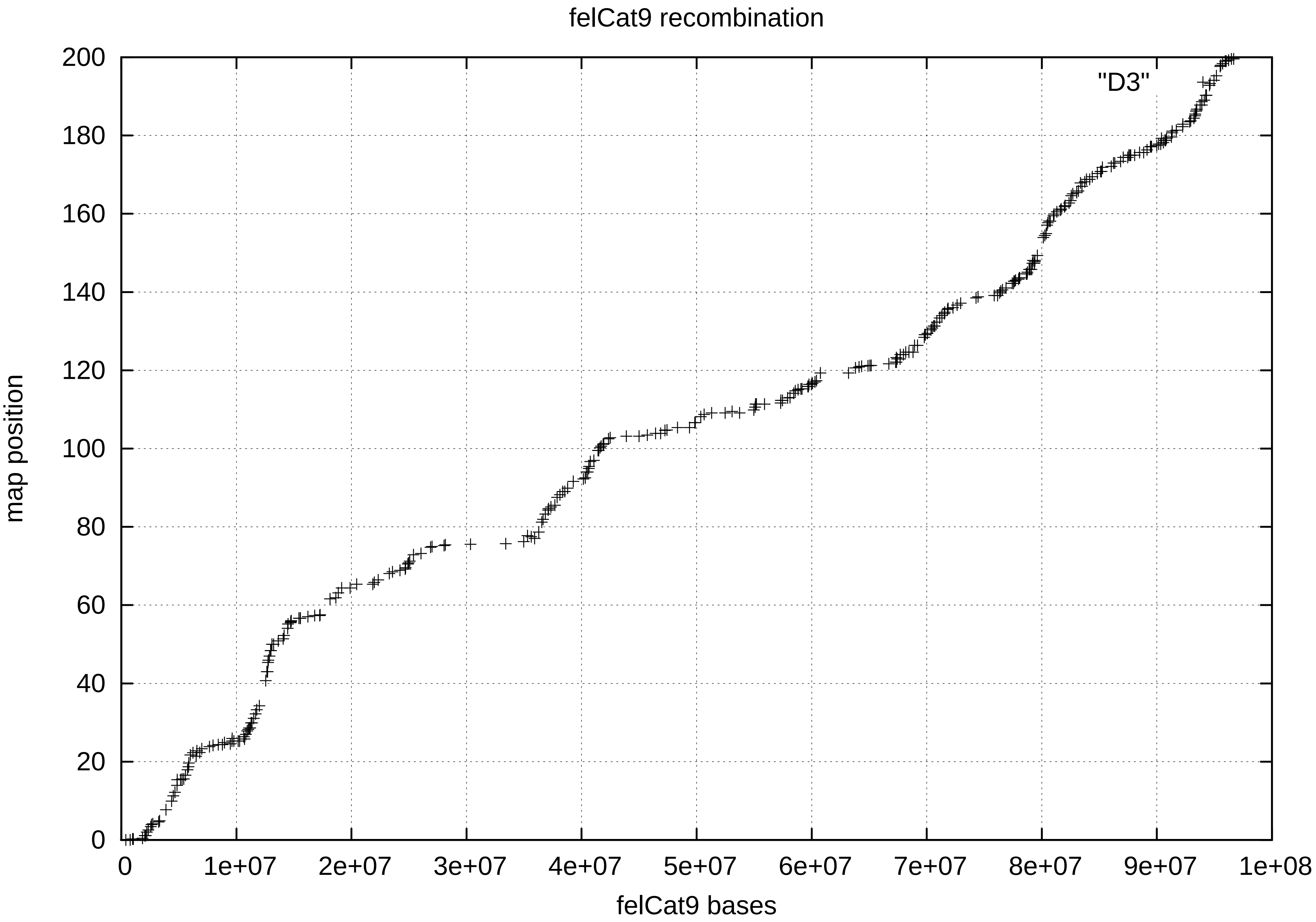
felCat9 recombination
"D3"
map position
0
20
40
60
80
100
120
140
160
180
200
0
1e+07
2e+07
3e+07
4e+07
5e+07
6e+07
7e+07
8e+07
9e+07
1e+08
felCat9 bases

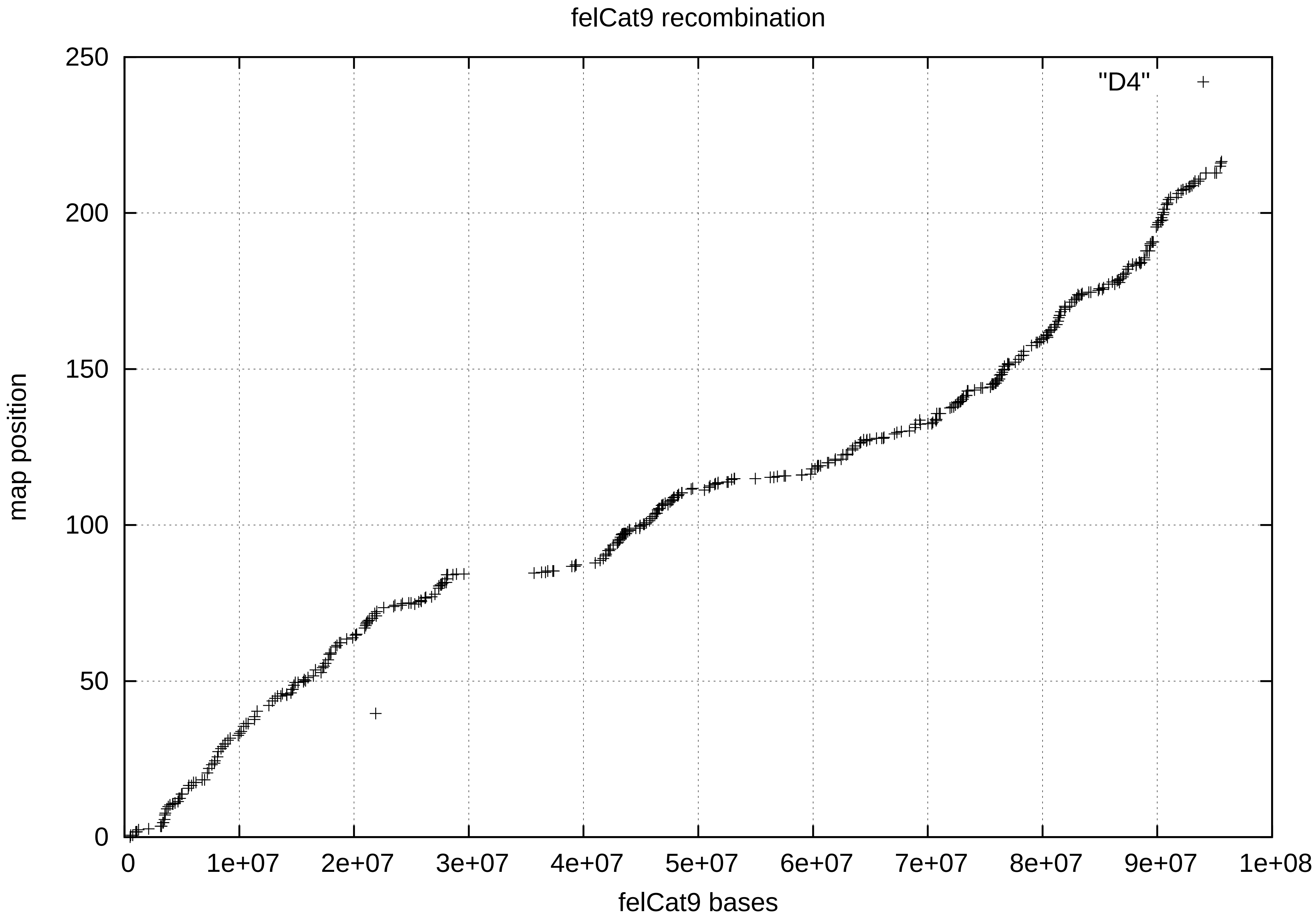

felCat9 recombination
"D4"
map position
felCat9 bases
0
50
100
150
200
250
0
1e+07
2e+07
3e+07
4e+07
5e+07
6e+07
7e+07
8e+07
9e+07
1e+08

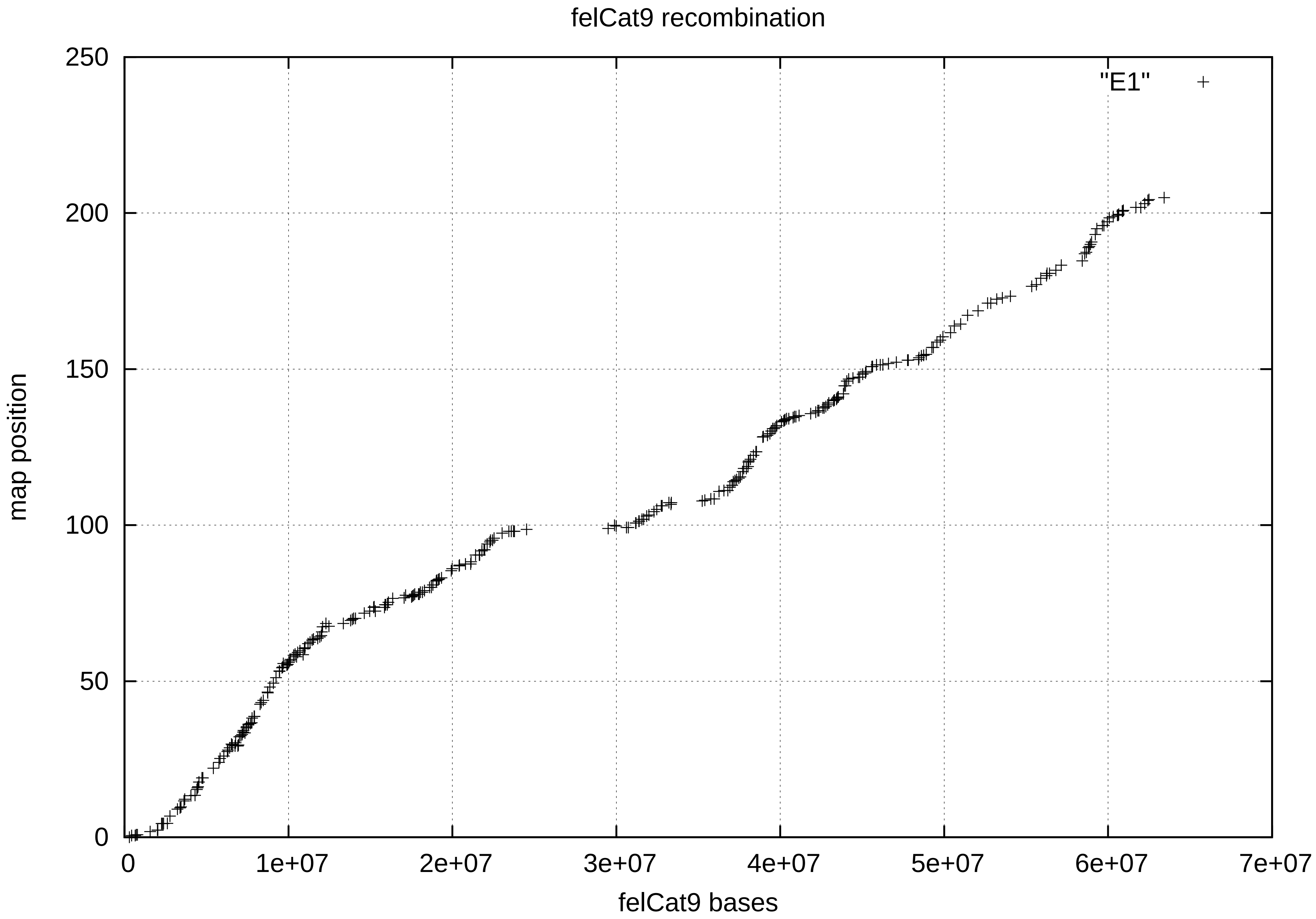
felCat9 recombination
"E1"
map position
felCat9 bases
0
50
100
150
200
250
0
1e+07
2e+07
3e+07
4e+07
5e+07
6e+07
7e+07

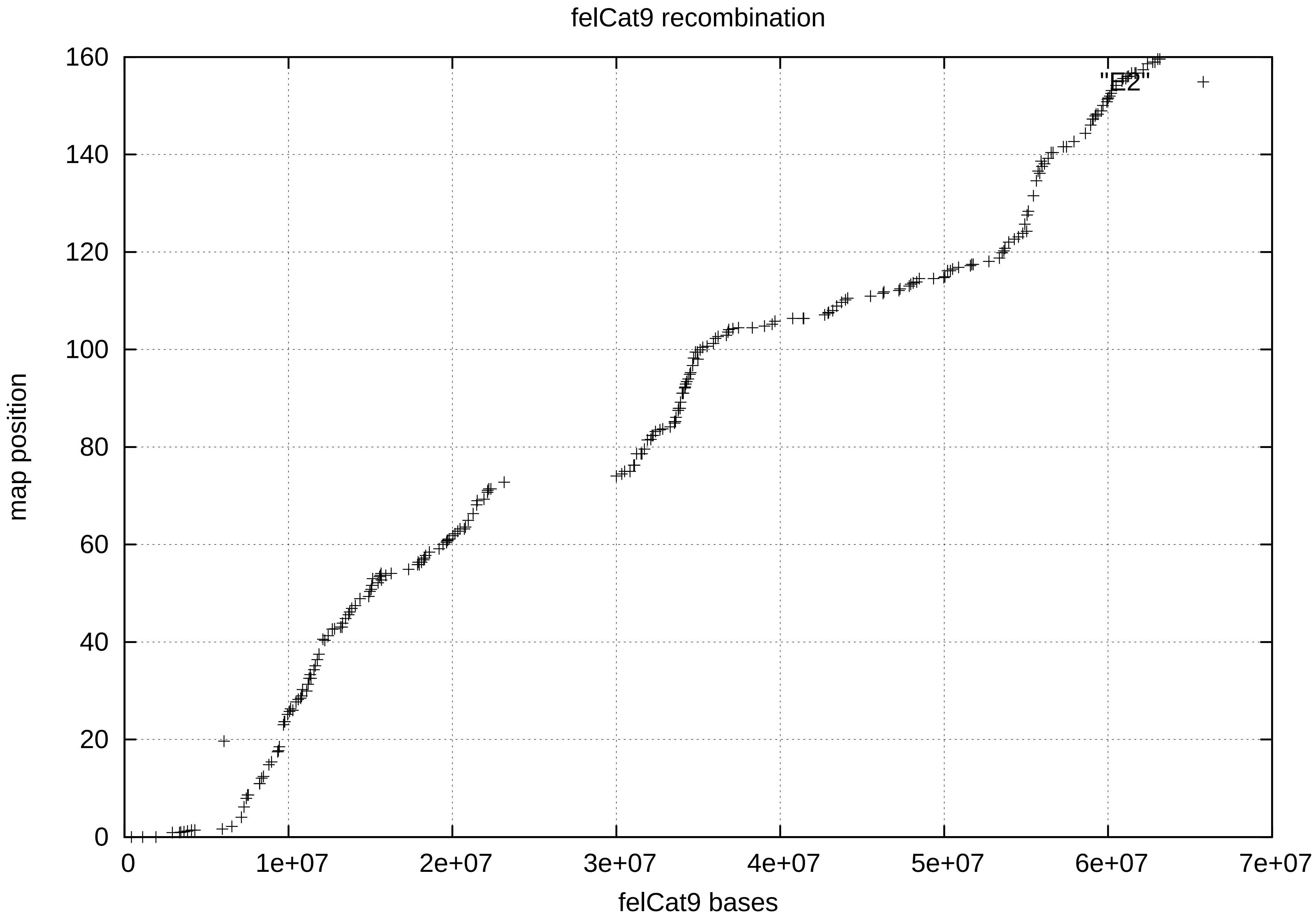
felCat9 recombination
map position
felCat9 bases
"E2"
0
20
40
60
80
100
120
140
160
0
1e+07
2e+07
3e+07
4e+07
5e+07
6e+07
7e+07

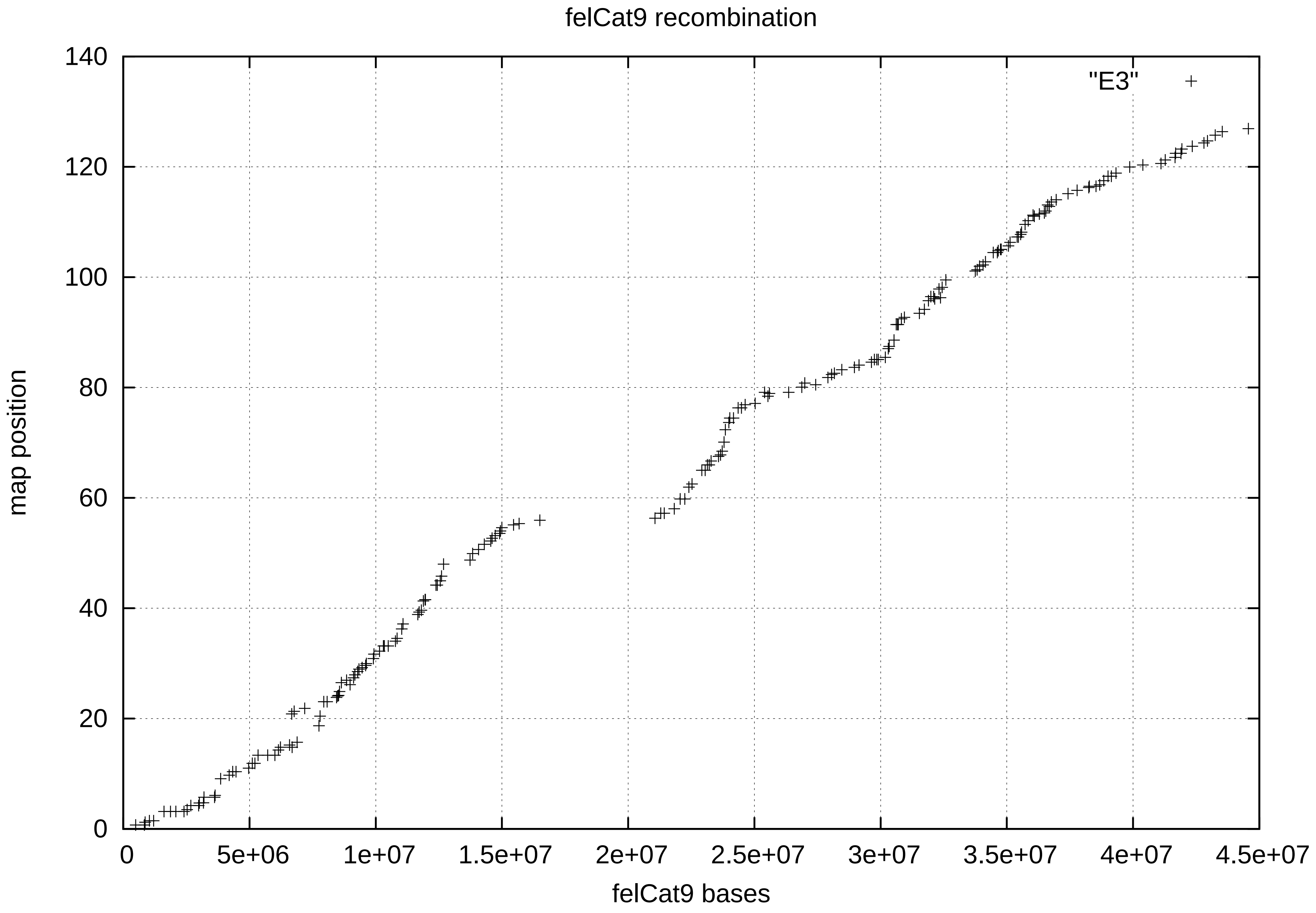
felCat9 recombination
"E3"
map position
felCat9 bases
0
20
40
60
80
100
120
140
0
5e+06
1e+07
1.5e+07
2e+07
2.5e+07
3e+07
3.5e+07
4e+07
4.5e+07

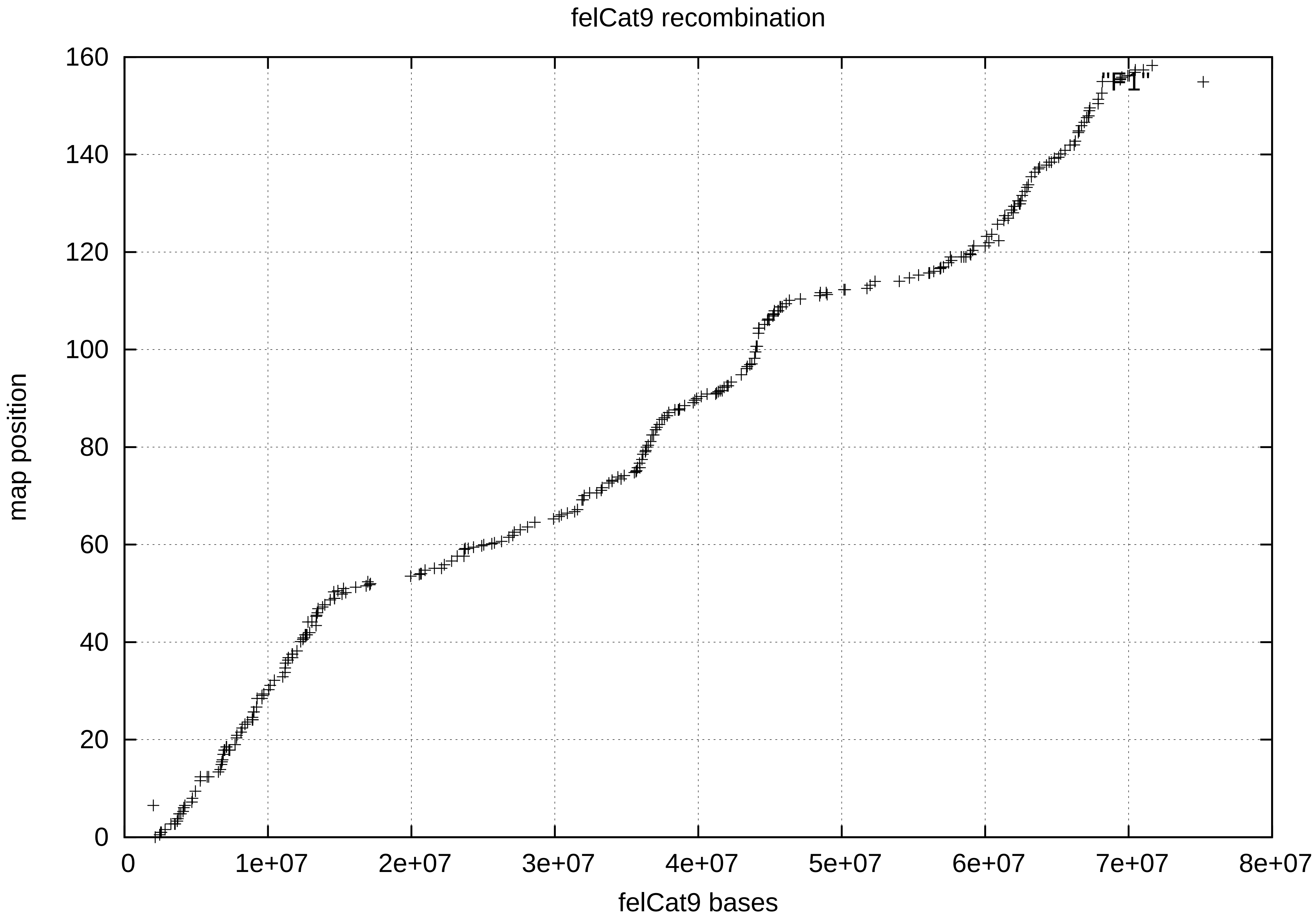
felCat9 recombination
"F1"
map position
felCat9 bases
0
20
40
60
80
100
120
140
160
0
1e+07
2e+07
3e+07
4e+07
5e+07
6e+07
7e+07
8e+07

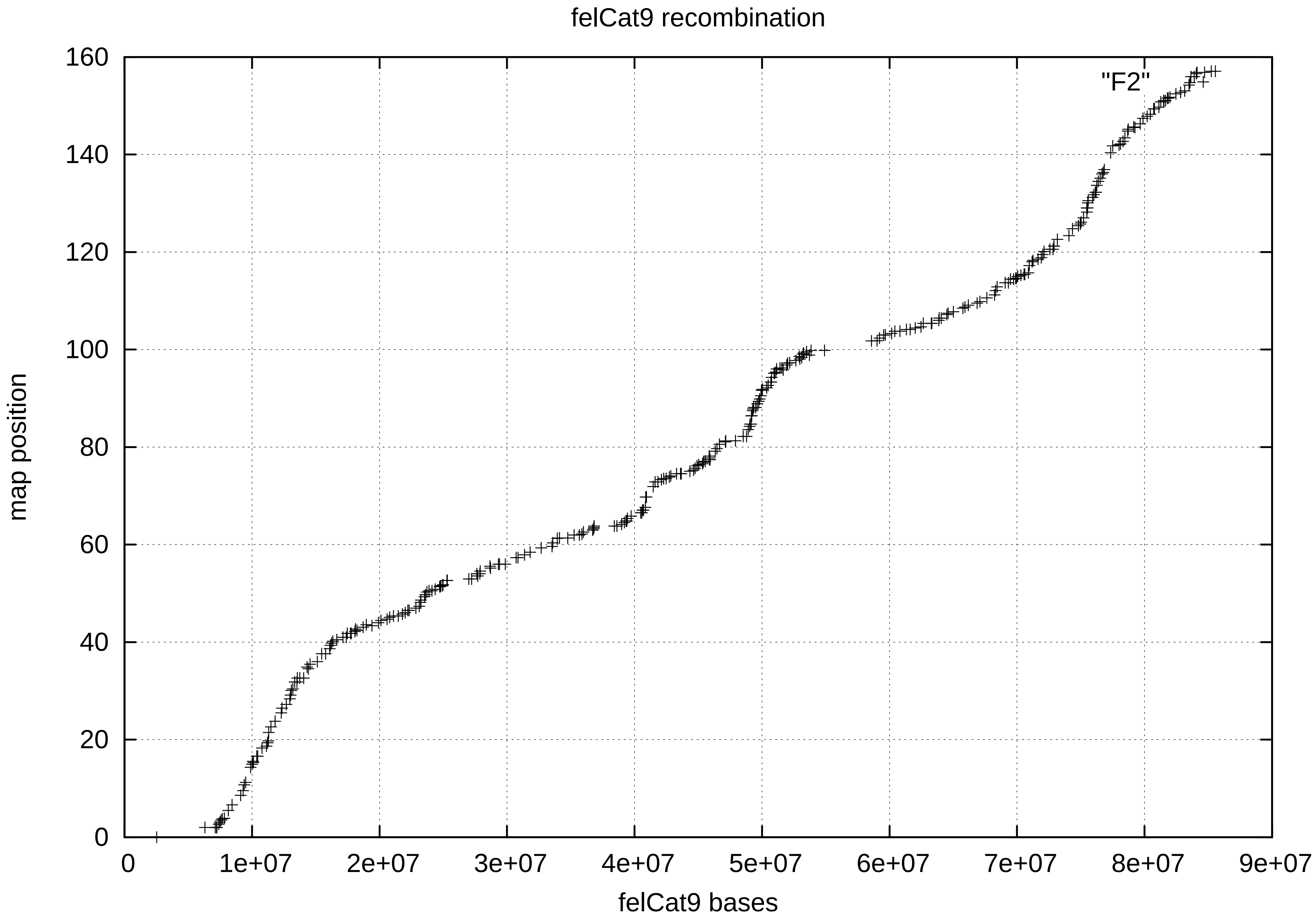

felCat9 recombination
"F2"
map position
felCat9 bases
0
20
40
60
80
100
120
140
160
1e+07
2e+07
3e+07
4e+07
5e+07
6e+07
7e+07
8e+07
9e+07

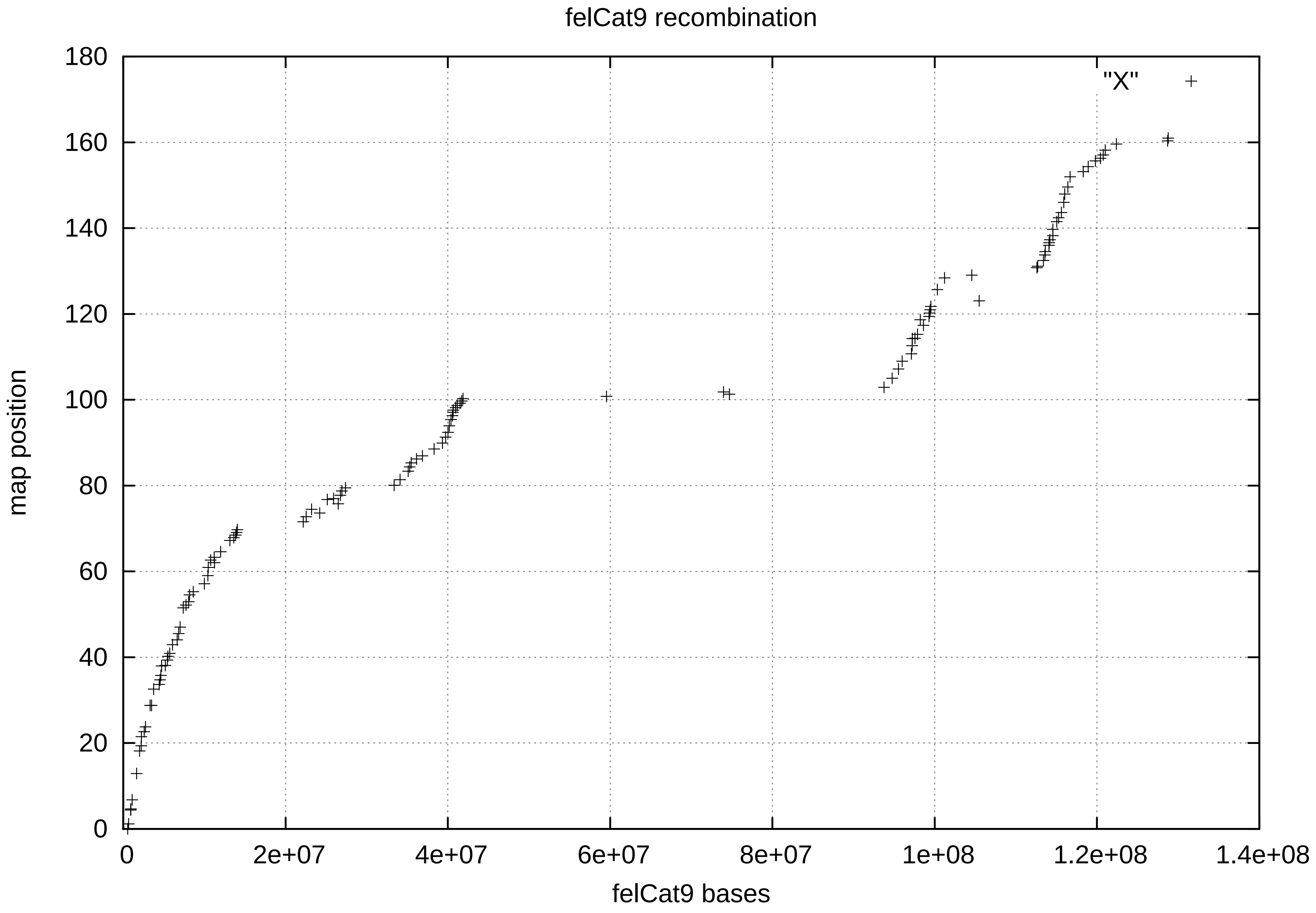
felCat9 recombination
map position
felCat9 bases
"X"
0
20
40
60
80
100
120
140
160
180
0
2e+07
4e+07
6e+07
8e+07
1e+08
1.2e+08
1.4e+08
